# Comprehensive multiomics analysis of cuproptosis-related gene characteristics in hepatocellular carcinoma

**DOI:** 10.3389/fgene.2022.942387

**Published:** 2022-09-06

**Authors:** Jie Fu, Sixue Wang, Zhenghao Li, Wei Qin, Qing Tong, Chun Liu, Zicheng Wang, Zhiqiang Liu, Xundi Xu

**Affiliations:** ^1^ Department of General Surgery, The Second Xiangya Hospital of Central South University, Changsha, China; ^2^ Department of Obstetrics and Gynecology, The Second Xiangya Hospital of Central South University, Changsha, China; ^3^ Department of General Surgery, South China Hospital of Shenzhen University, Shenzhen, China

**Keywords:** cuproptosis-related genes, multiomics data, risk score, immune characteristics, single-cell, hepatocellular carcinoma

## Abstract

**Background:** The mechanism of copper-induced cell death, which is called cuproptosis, has recently been clarified. However, the integrated role of cuproptosis-related genes in hepatocellular carcinoma (HCC) and its relationship with immune characteristics are still completely unknown.

**Methods:** In this study, the expression, genetic, and transcriptional regulation states of 16 cuproptosis-related genes in HCC were systematically investigated. An unsupervised clustering method was used to identify distinct expression patterns in 370 HCC patients from the TCGA-HCC cohort. Differences in functional characteristics among different expression clusters were clarified by gene set variation analysis (GSVA). The abundances of immune cells in each HCC sample were calculated by the CIBERSORT algorithm. Next, a cuproptosis-related risk score was established based on the significant differentially expressed genes (DEGs) among different expression clusters.

**Results:** A specific cluster of HCC patients with poor prognosis, an inhibitory immune microenvironment, and high expression levels of immune checkpoint molecules was identified based on the expression of the 16 cuproptosis-related genes. This cluster of patients could be well-identified by a cuproptosis-related risk score system. The prognostic value of this risk score was validated in the training and two validation cohorts (TCGA-HCC, China-HCC, and Japan-HCC cohorts). Moreover, the overall expression status of the cuproptosis-related genes and the genes used to establish the cuproptosis-related risk score in specific cell types of the tumor microenvironment were preliminarily clarified by single-cell RNA (scRNA) sequencing data.

**Conclusion:** These results indicated that cuproptosis-related genes play an important role in HCC, and targeting these genes may ameliorate the inhibitory immune microenvironment to improve the efficacy of immunotherapy with immune checkpoint inhibitors (ICIs).

## Introduction

Liver cancer is one of the most life-threatening cancer types worldwide, and its main pathological type is hepatocellular carcinoma (HCC) (Sung et al., 2021). At present, the best treatment for HCC is still radical resection. However, many patients have lost the chance of radical surgery at the time of diagnosis (Su et al., 2022). Although there are some treatments for advanced unresectable HCC, such as transcatheter arterial chemoembolization (TACE), radiofrequency thermal ablation (RFTA), and molecular targeted drugs, including tyrosine kinase inhibitors (TKIs) such as sorafenib and lenvatinib, the curative effects are still unsatisfactory (Forner et al., 2018; Kim et al., 2021; Chen et al., 2022; Yau et al., 2022). Therefore, it is urgent to find more effective therapeutic targets or methods for combining existing treatments to improve the therapeutic effects of HCC treatment and prognosis of HCC patients.

Trace metals, such as iron, copper, and zinc, are essential for life activities (Liu et al., 2021; Dou et al., 2022; Yang et al., 2022). It is important that the content of these metals in cells be appropriate. Low intracellular metal concentrations may inhibit the function of important metal-binding enzymes, thus impairing normal biological activities, while exorbitant intracellular metal concentrations can lead to cell death and disease, such as Wilson’s disease (Collins et al., 2021; Dou et al., 2022). Metal ion-related cell death patterns, such as ferroptosis, have been investigated in various cancers (Gan, 2022; Lei et al., 2022). Recently, the mechanism of copper-induced cell death, called cuproptosis, was also clarified (Tsvetkov et al., 2022). Some cuproptosis-related genes have also been studied in HCC in recent years. For example, copper transporter genes (ATP7A, ATP7B, SLC31A1, and SLC31A2) have been reported to participate in HCC progression by regulating intracellular copper homeostasis (Davis et al., 2020). Furthermore, ATP7B has been shown to be associated with platinum resistance in HCC cell lines (Guttmann et al., 2018). However, these studies have only studied the role of one or several copper transporter genes, and the integrated role of cuproptosis-related genes in HCC remains largely unknown.

Immunotherapy with immune checkpoint inhibitors (ICIs) has shown good efficacy in treatment of a variety of cancers (Petroni et al., 2022; Strickland et al., 2022). Monoclonal antibodies targeting PD-1 or PD-L1 combined with angiogenesis inhibitors or TKIs have also enabled considerable progress in the treatment of advanced HCC (Finn et al., 2020; Llovet et al., 2021). However, there are only a few patients with good treatment response. Thus, elucidating the immune heterogeneity of HCC will help clinicians judge which patients are likely to benefit from immunotherapy and will facilitate screening of synergistic therapeutic targets to enhance therapeutic efficacy. Numerous studies have clarified the connection between ferroptosis and immune characteristics (Friedmann Angeli et al., 2022; Gan, 2022). In addition, several studies have reported that copper is correlated with immune activity (Fu et al., 2014; Portelinha et al., 2021). However, the combined roles of cuproptosis-related genes in immune characteristics remain unclear.

In this study, genetic and transcriptional regulation features of cuproptosis-related genes were systematically investigated. Next, three distinct cuproptosis expression patterns were identified by an unsupervised clustering method based on a cuproptosis-related gene list. Differences in prognosis, function, and immune characteristics among these three clusters were clarified. In addition, a cuproptosis-related risk score was established to quantify cuproptosis-related characteristics. Independent prognostic values of the risk score were validated in a training cohort and two validation cohorts. High-risk scores predict poor prognosis, an inhibitory immune microenvironment, and high expression levels of immune checkpoint molecules in HCC patients. These results suggest that cuproptosis-related genes play an important role in HCC, which will help us assess the prognosis of HCC patients and their response to immunotherapy, and these genes may become potential synergistic targets to improve the efficacy of immunotherapy for HCC.

## Materials and methods

### Data acquisition and preprocessing

Cuproptosis-related gene lists were acquired from recently published literature (Tsvetkov et al., 2022). In detail, a total of 16 cuproptosis-related genes, including three copper transporters (SLC32A1, ATP7A, and ATP7B), ferredoxin 1 (FDX1), four lipoic acid (LA) pathway molecules (LIAS, DLD, LIPT1, and LIPT2), seven pyruvate dehydrogenase complex (PDHC) molecules (DLAT, PDHA1, PDHB, MTF1, GLS, CDKN2A, and GCSH), and an elesclomol-induced cuproptosis-related molecule (MPC1), were analyzed in the present study. Gene expression data in various normal tissues and organs of the human body were downloaded from the Genotype-Tissue Expression (GTEx) database (https://gtexportal.org/home/) (GTEx Consortium, 2013). Gene expression data in fragments per kilobase million (FPKM) or count forms, as well as DNA methylation (Illumina Infinium Human Methylation450 platform), tumor mutation burden (TMB), copy number variation (CNV) data, and their corresponding clinicopathological characteristics of HCC patients, were downloaded from The Cancer Genome Atlas (TCGA) database (https://portal.gdc.cancer.gov/) as a training cohort (TCGA-HCC). In addition, gene expression data and corresponding clinical information from the Zhongshan Hospital of Fudan University (China-HCC) and LIRI-JP cohorts (Japan-HCC) were downloaded from the NODE (https://www.biosino.org/node) and the International Cancer Genome Consortium (ICGC) databases (https://dcc.icgc.org/) as validation cohort 1 and validation cohort 2, respectively (Fujimoto et al., 2016; Gao et al., 2019). TMB data were analyzed by the “maftools” package in R software, and CNV data were analyzed by the R software and online tool GISTIC 2.0 (https://cloud.genepattern.org/gp/pages/login.jsf) (Mermel et al., 2011). FPKM data were transformed into transcripts per million (TPM) data before analysis. The transcription factors and miRNA regulatory networks of these cuproptosis-related genes were analyzed by the online tool NetworkAnalyst (https://www.networkanalyst.ca/) (Zhou et al., 2019). Except for this online network analysis, all of the data were analyzed by R x64 4.1.0 in this study.

### Clustering, survival, and immune analyses and gene set variation analysis

Unsupervised clustering analysis was used to identify the distinct expression patterns of the HCC patients in the training cohort based on the expression data of these 16 cuproptosis-related genes using the “ConsensusClusterPlus” package (Wilkerson and Hayes, 2010). Prognosis differences among the different clusters were analyzed by the “survMisc” and “survminer” packages. The abundances of immune cells in each HCC sample were calculated by the CIBERSORT algorithm in R software based on the gene expression data and LM22 file provided by the function developer (Newman et al., 2015). In addition, the overall difference in gene function among the different clusters was investigated by the “GSVA” package using the “c2. cp.kegg.v7.2. symbols.gmt” gene set (Hänzelmann et al., 2013). Correlations between levels of cuproptosis-related genes and immune cells were analyzed by the Spearman method.

### Differential analysis and functional annotation

To further explore the gene expression characteristics related to cuproptosis, differentially expressed genes (DEGs) between the cluster-1 and cluster-2 or cluster-3 were identified respectively by the “DESeq2” package according to the count data (Love et al., 2014), considering that cluster-1 has significantly different characteristics (Figure 3C). Significant DEGs were screened by the criteria of an adjusted *p* value <0.05 and |log2FoldChange| > 1. Next, DEGs were further identified by taking the intersection of the DEGs of cluster-1 and cluster-2 and the DEGs of cluster-1 and cluster-3. Subsequently, Gene Ontology (GO) and Kyoto Encyclopedia of Genes and Genomes (KEGG) analyses were performed by the “clusterProfiler” package using significant DEGs (Yu et al., 2012).

### Establishment and evaluation of the cuproptosis-related risk score

To quantify the cuproptosis expression patterns of individual HCC patients, a cuproptosis-related risk score system was established. The detailed information for the generation of the cuproptosis-related risk score is as follows. First, prognostic DEGs were identified by the random forest model (ntree = 100) using the “survivalsvm” and “randomForestSRC” packages, which were further screened by univariate and multivariate Cox analyses. As a result, a cuproptosis-related risk score was established based on the remaining 19 most significant prognostic DEGs (*p* value <0.05) and their risk coefficients from multivariate Cox analysis. The calculation formula is as follows: ∑ (Exp * Coef), where Exp = expression levels of the prognostic DEGs and Coef = risk coefficients of the prognostic DEGs. The HCC patients in each cohort were divided into two groups (high- and low-score groups) according to the optimal cutoff value of the risk score automatically calculated by the “roc” method in the “ggrisk” package, and their prognoses were identified by Kaplan–Meier plotter curves. In addition, potential drugs targeting these 19 prognostic genes were screened by CellMiner, a web tool based on the NCI-60 cell line set (Reinhold et al., 2019).

### Processing and analysis of clinicopathological characteristics

Clinicopathological characteristics with a missing rate of less than 20% from the TCGA-HCC cohort were analyzed in our study, including age (“≤60y” and “>60y”), stage (“Stage I–II” and “Stage III–IV”), T-stage (“T1–T2” and “T3–T4”), histological grade (“G1–G2” and “G3–G4”), hepatitis [“Hepatitis (-)” and “Hepatitis (+)”], surgical margin (“R0” and “R1–R2”), gender (“female” and “male”), and vascular invasion (“None” and “Macro–Micro”). The missing values were interpolated by the “mice” package. Independent prognostic values of these clinicopathological characteristics and the risk score were investigated by univariate and multivariate Cox regression analyses in the training and validation cohorts (TCGA-HCC, China-HCC, and Japan-HCC cohorts). Finally, subgroup analysis was performed using the “forestplot” package.

### Analysis of single-cell RNA sequencing data

ScRNA sequencing data of HCC and adjacent liver tissues from 12 primary HCC patients and six relapsed HCC patients were downloaded from the China National GeneBank DataBase (CNSA: CNP0000650) (Sun et al., 2021). After screening, all 16,498 effective cells were identified as 24 clusters (from cluster-0 to cluster-23) and annotated as 10 cell types [T cells, myeloid cells, malignant cells, NK cells, B cells, endothelial cells, epithelial cells, plasmacytoid dendritic cells (pDCs), hepatic stellate cells (HSCs), and plasma cells] by the data provider. The proportions of cuproptosis-related genes and prognostic DEGs in each cell cluster were calculated by the “PercentageFeatureSet” function using the “Seurat” package in R (Satija et al., 2015). In addition, DEGs between tumor cell cluster-C12 and other cell clusters were identified by the “FindMarkers” function. Significant DEGs in C12 were screened by |average log2FC| > 2 and adjusted *p* value <0.05.

### Statistical analysis

All of the data were analyzed and visualized by R 4.1.0 in this study. The survival data were statistically analyzed by the log-rank test. The continuous variables between the two groups were compared by Student’s t-test and Wilcoxon test. Difference comparisons of three groups were conducted by one-way ANOVA and Kruskal–Wallis tests. *p* value <0.05 was considered statistically significant.

## Results

### Gene expression, genetic alteration, and transcription regulation status of the 16 cuproptosis-related genes

**FIGURE 1:**
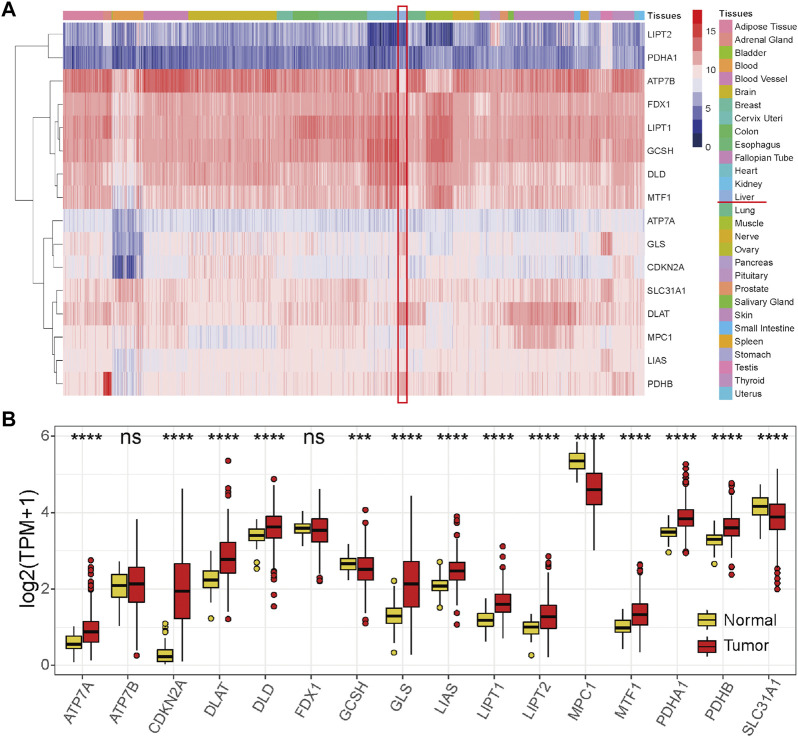
Expression profiles of the 16 cuproptosis-related genes. **(A)** Expression profile of the 16 cuproptosis-related genes in normal tissues and organs of the human body from the GTEx database. **(B)** Expression profile of the 16 cuproptosis-related genes in the HCC tumor tissues and normal tissues in the TCGA-HCC cohort. Red represents tumor tissues, and yellow represents normal liver tissues. *p < 0.05, **p < 0.01, ***p < 0.001, and ****p < 0.0001, ns: not significant.

We first explored the expression levels of the 16 cuproptosis-related genes in various normal tissues and organs of the human body. The results showed that the expression profiles of these genes in tissues and organs of the whole body were similar, except that a few genes were expressed at low or high levels in specific tissues and organs, such as low expression levels of ATP7A and CDKN2A in blood and high expression levels of GLS and DLAT in the liver (Figure 1A). Next, the expression differences between HCC and normal liver samples were compared in the TCGA-HCC cohort. The results showed that the levels of most cuproptosis-related genes were significantly highly expressed in tumor samples, except for GCSH, MPC1, and SLC31A1, while levels of ATP7B and FDX1 showed no significant differences between the two groups (Figure 1B). These results revealed that cuproptosis-related genes are significantly differentially expressed in HCC.

**FIGURE 2:**
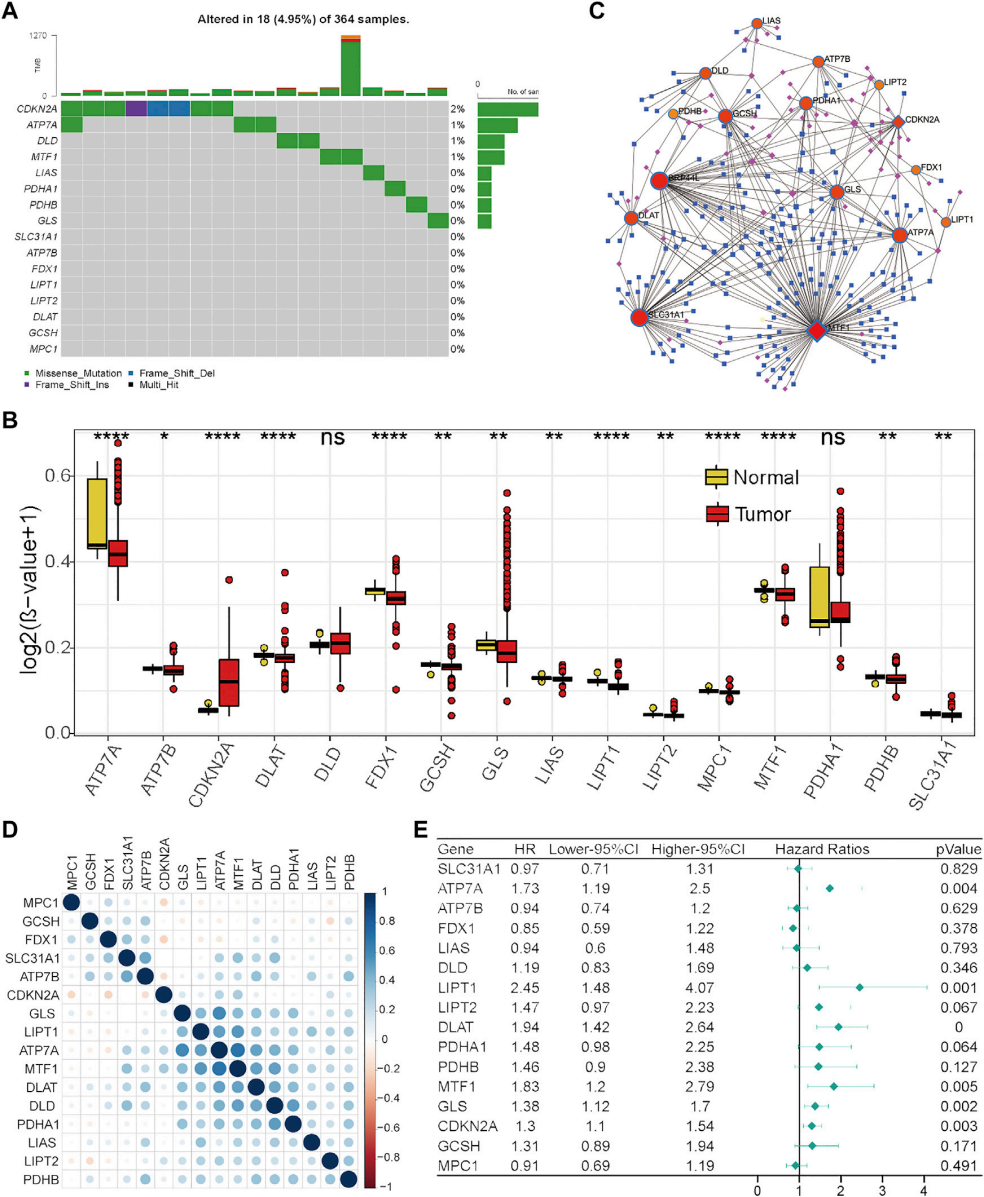
Regulation, interaction, and prognostic characteristics of the 16 cuproptosis-related genes in the TCGA-HCC cohort. **(A)** TMB status of the 16 cuproptosis-related genes in 364 HCC patients. **(B)** Differential DNA methylation levels between HCC tumor tissues and normal tissues. **(C)** Transcription factor and miRNA regulation network of cuproptosis-related genes. Red represents cuproptosis-related genes, pink represents transcription factors, and blue represents miRNAs. **(D)** Correlations among cuproptosis-related genes. **(E)** Forest plot results of univariate analysis of cuproptosis-related genes.

To further explore the reasons for the change in cuproptosis-related gene expression in HCC, genetic alterations were analyzed. However, TMB (Figure 2A) or CNV (Supplementary Table S1) only occurred in a few genes, suggesting that genetic factors may not be the main reason for the change in cuproptosis-related gene expression. Next, the main type of epigenetic factor, DNA methylation status, was investigated. The results showed that the DNA methylation status of these cuproptosis-related genes was generally different between tumor and normal liver tissues (Figure 2B). We noticed that eight genes (ATP7A, DLAT, GLS, LIAS, LIPT1, LIPT2, MTF1, and PDHB) that were significantly highly expressed in tumors exhibited DNA hypomethylation. These results indicated that the change in cuproptosis-related gene expression in HCC may be caused partially by the DNA methylation status. Under this condition, the potential miRNA and transcription factor regulatory network was preliminarily explored (Figure 2C), which was also one of the factors underlying differential gene expression.

Next, Pearson’s correlation analysis was performed to assess the correlations between the levels of these cuproptosis-related genes to investigate their integrated role. We found that positive correlations were more frequent, and there was a strong correlation between the levels of seven genes (GLS, LIPT1, ATP7A, MTF1, DLAT, DLD, and PDHA1) (Figure 2D). Furthermore, the prognostic values of these cuproptosis-related genes were investigated by univariate Cox analysis. As revealed by Figure 2E, ATP7A, LIPT1, DLAT, MTF1, GLS, and CDKN2A were adverse prognostic factors in HCC. Taken together, these results indicate that cuproptosis-related genes might play an important role in HCC.

### Distinct expression patterns of cuproptosis-related genes were associated with genetic alterations

**FIGURE 3:**
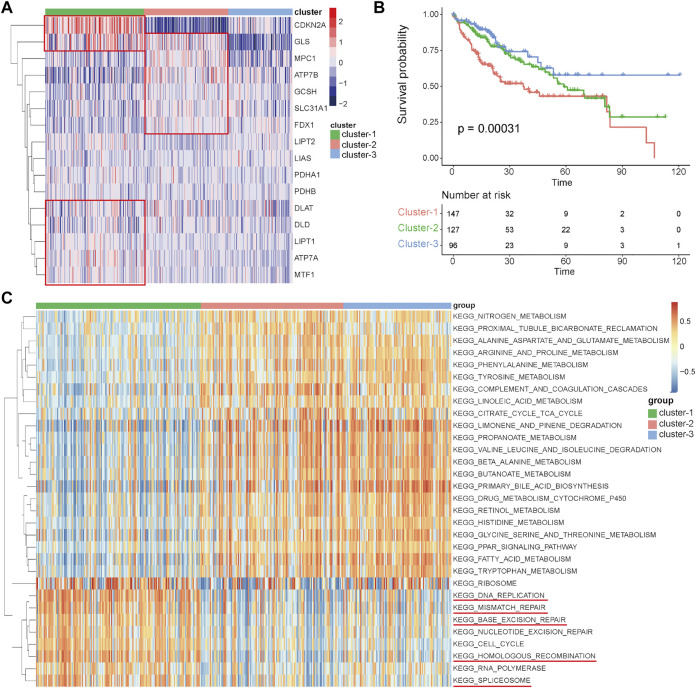
Identification of distinct expression patterns and analysis of their prognostic and functional characteristics in the TCGA-HCC cohort. **(A)** Unsupervised clustering of HCC patients based on the 16 cuproptosis-related genes. **(B)** Survival analyses for the three distinct expression patterns. **(C)** GSVA results showing the functional characteristics in the three clusters.

To comprehensively clarify the influence of these cuproptosis-related genes in HCC, distinct expression patterns were discriminated by an unsupervised clustering method based on the expression levels of these 16 cuproptosis-related genes in the TCGA-HCC cohort. According to the optimal number of clusters automatically calculated by the “ConsensusClusterPlus” package (Supplementary Figures S1A–F), three clusters were identified across 370 HCC samples (Figure 3A), and most of the cuproptosis-related genes were differentially expressed among these three clusters (Supplementary Figure S1G). The results of prognostic analysis revealed that patients in cluster-1 had a significant survival disadvantage (Figure 3B). To explore the biological characteristics among these three distinct clusters, GSVA was conducted. As shown in Figure 3C, cluster-1 has significantly different biological characteristics from those of cluster-2 and cluster-3, while the characteristics of cluster-2 and cluster-3 are similar. Interestingly, most of the top nine functional terms enriched in cluster-1 were associated with genetic alterations, such as mismatch repair, base exclusion repair, nucleotide exclusion repair, and homologous recombination. These results indicated that cuproptosis-related genes may affect the prognosis of HCC patients by affecting genome stability.

### Analysis of immune characteristics among the three clusters

**FIGURE 4:**
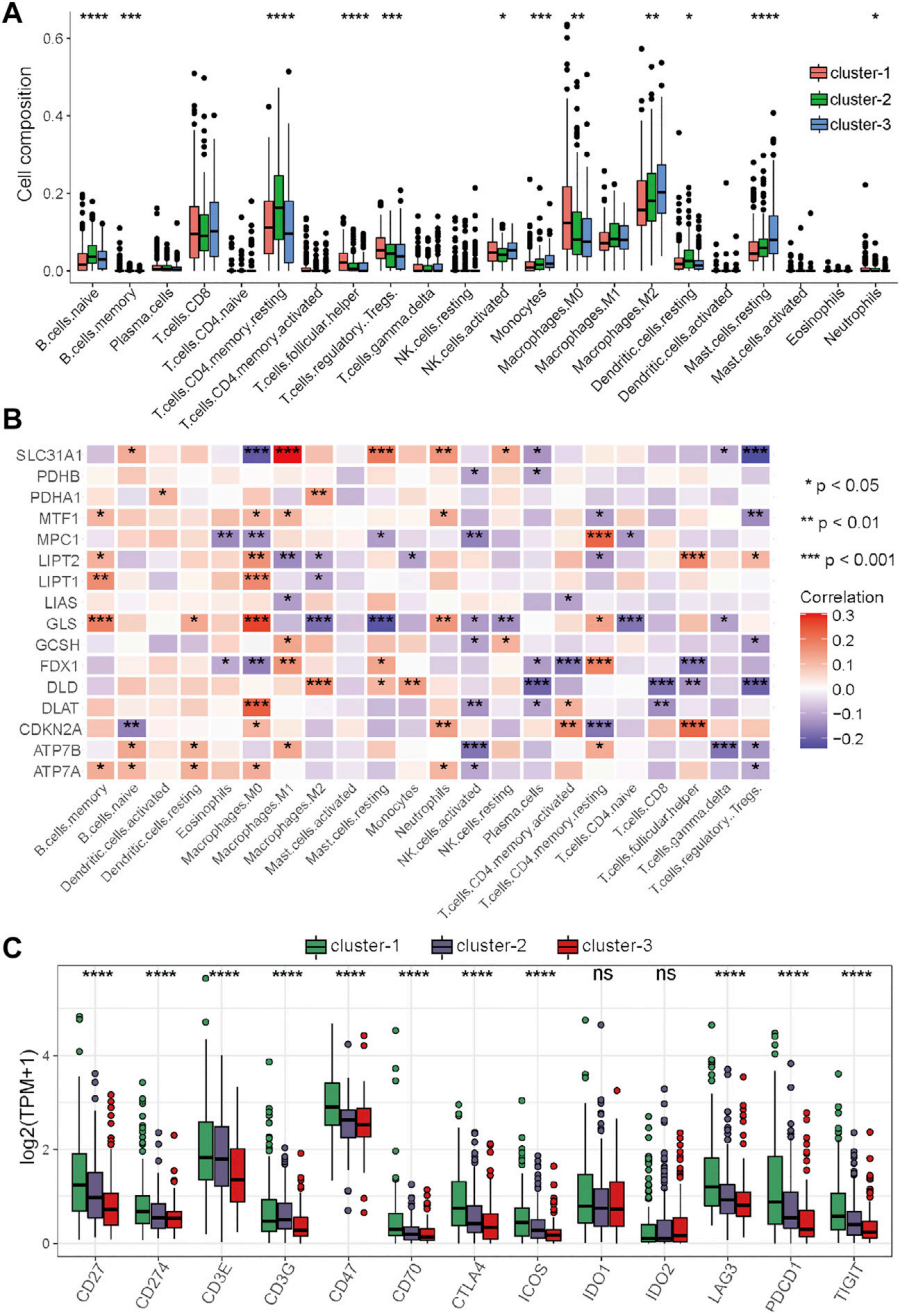
Differential analysis of immune characteristics among the three clusters in the TCGA–HCC cohort. **(A)** Differences in the 22 immune cell types among the three expression clusters. **(B)** Correlations between the 16 cuproptosis-related genes and 22 immune cells. **(C)** Differences in the 13 immune checkpoint molecules among the three expression clusters. *p < 0.05, **p < 0.01, ***p < 0.001, and ****p < 0.0001.

Numerous studies have emphasized the important role of the immune microenvironment in the prognosis and therapeutic response of HCC patients (Dong et al., 2020; Llovet et al., 2021). A recent study revealed that magnesium can promote CD8 T cell activation and high expression levels of PD-1 by regulating metabolic reprogramming to enhance the efficacy of immunotherapy (Lötscher et al., 2022). In addition, some studies have shown that copper is related to T cell infiltration and recruitment (Kaddatz et al., 2021; Wang et al., 2021). However, whether these cuproptosis-related genes can affect the immune characteristics of HCC remains largely unknown. In this study, immune cell abundances in HCC samples were calculated by the CIBERSORT function in R, and the differences in immune characteristics among these three clusters were compared. There were significant differences among these three clusters, such as in follicular helper T cells, regulatory T cells, and M0 macrophages, which were significantly more abundant in cluster-1 than in cluster-2 and cluster-3, while monocytes, M1 macrophages, M2 macrophages, and resting mast cells were significantly less abundant in cluster-1 (Figure 4A). These results indicate that there may be an immunosuppressive microenvironment in cluster-1 involving inhibitory immune cell infiltration and inhibition of macrophage polarization.

To further clarify the role of these cuproptosis-related genes in the immune microenvironment of HCC, Spearman’s correlation analysis was performed to assess the correlations between these genes and immune cells. The results showed that cuproptosis-related genes have strong correlations with many immune cells, for example, the levels of SLC31A1 were positively associated with the abundance of M1 macrophages, the levels of GLS were positively associated with the abundance of M0 macrophages, and the levels of SLC31A1 was positively associated with the abundance of M0 macrophages and regulatory T cells (Figure 4B). Immune checkpoint inhibitors (ICIs) are the main drugs for tumor immunotherapy at present, so the differential expression status of immune checkpoints among the three clusters was further investigated. To our surprise, most of the main immune checkpoints, such as CTLA4, PDCD1, CD274, ICOS, and LAG3, were expressed at significantly high levels in cluster-1 (Figure 4C). Taken together, these results suggested that the proportion of immune cells in cluster-1 was quite different from that in the two other clusters, and most of the major immune checkpoints were highly expressed in cluster-1. ICIs may be more effective for HCC patients in cluster-1, and targeting these cuproptosis-related genes may improve the inhibitory immune microenvironment to improve the efficacy of immunotherapy.

### Differential analysis, establishment, and external validation of the cuproptosis-related risk score

Considering that cluster-1 has distinct functional characteristics than the other two clusters (Figure 3C), DEGs between cluster-1 and cluster-2 or cluster-3 were identified. After screening by the criteria of an adjusted *p* value <0.05 and |log2FoldChange| > 1 and taking intersection of the DEGs of cluster-1 and cluster-2 and the DEGs of cluster-1 and cluster-3, a total of 3,854 significant DEGs were identified (Supplementary Table S2). Functional enrichment analyses were performed based on these significant DEGs. As revealed by Supplementary Figure S2A, the results of biological process (BP) analysis showed that these genes were enriched for nuclear division, organelle fission, chromosome segregation, sister chromatid segregation, etc. DEGs were enriched for cellular components (CC), including synaptic membrane, condensed chromosome, kinetochore, and centromeric region. DEGs were enriched for molecular function (MF), including gated channel activity, iron channel activity, and receptor–ligand activity. The results of KEGG analysis showed that DEGs were enriched for neuroactive ligand–receptor activity, cell cycle, drug metabolism, etc. (Supplementary Figure S2B).

**FIGURE 5:**
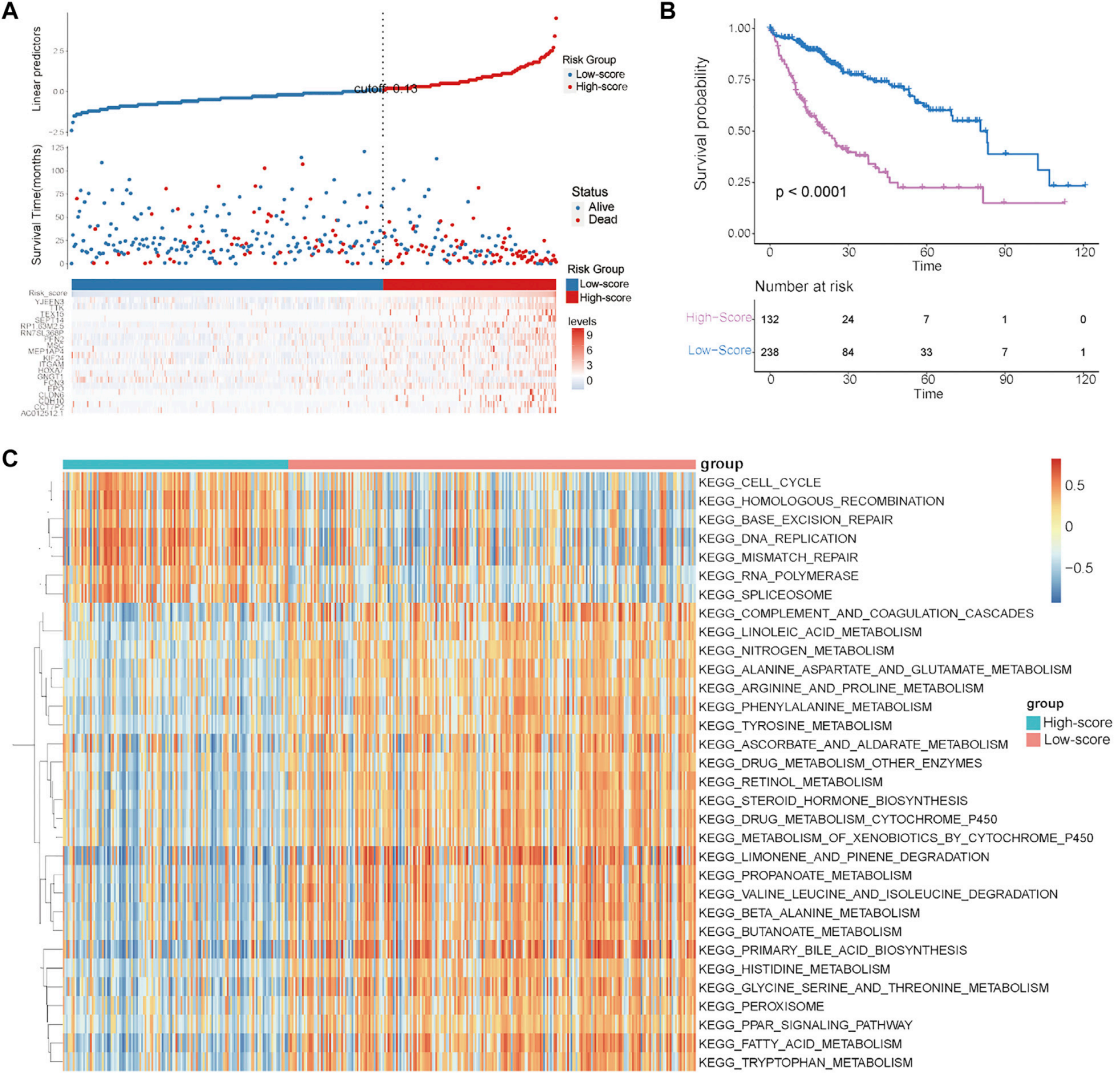
Establishment and evaluation of the cuproptosis-related risk score in the TCGA–HCC cohort. **(A)** Survival status of HCC patients and expression of the 19 significant prognostic DEGs in the high- and low-score groups. **(B)** Kaplan–Meier curve for the two risk groups. **(C)** GSVA results showing the functional characteristics in the two risk groups.

**FIGURE 6:**
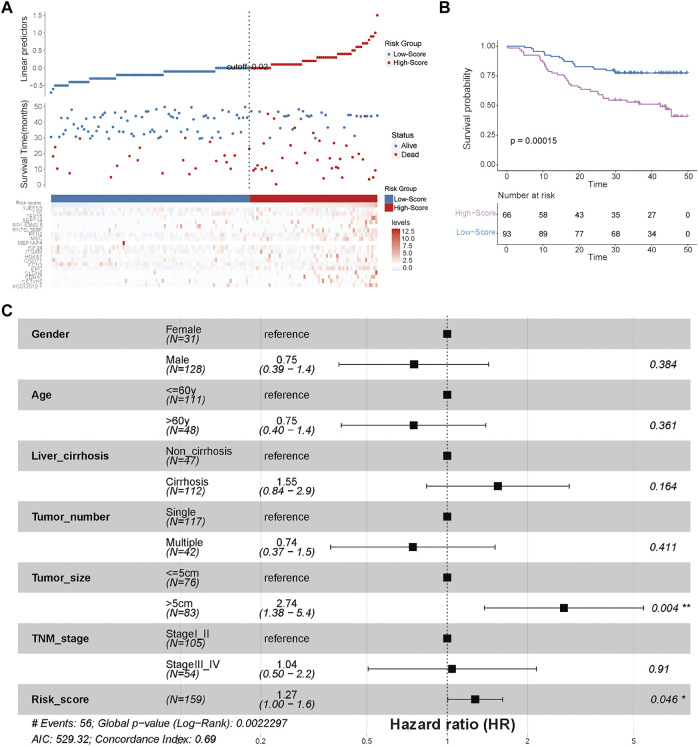
External validation of the efficiency of the cuproptosis-related risk score in validation cohort 1 (China-HCC). **(A)** Survival status of HCC patients and expression of the 19 significant prognostic DEGs in the high- and low-score groups. **(B)** Kaplan–Meier curve for the two risk groups in the China-HCC cohort. **(C)** Forest plot of clinicopathological features and risk scores in the China-HCC cohort.

To quantify the cuproptosis expression patterns of individual HCC patients, a cuproptosis-related risk score was established. First, a total of 168 prognostic genes among these 3,854 DEGs were identified by the random forest method and were further screened by subsequent univariate and multivariate Cox analyses (Supplementary Table S3). As a result, a cuproptosis-related risk score was generated based on the remaining 19 most significant prognostic DEGs and their risk coefficients (Table 1). HCC patients in the TCGA-HCC cohort were separated into two risk groups (high- and low-score groups) according to the optimal cutoff value of the risk score automatically calculated by the “roc” method in the “ggrisk” package (Figure 5A). The results of survival analyses showed that most of these 19 prognostic genes were highly expressed in the high-score group (Figure 5A), and patients in the high-score group had a significantly poorer prognosis than those in the low-score group (Figure 5B). In addition, most of these 19 prognostic genes and the risk score were highly expressed in cluster-1 (Supplementary Figures S2C,D). Next, the prognostic value of the risk score was further validated by two external HCC cohorts (China-HCC and Japan-HCC cohorts). In these two cohorts, risk scores were calculated using the same formula as that used in the TCGA-HCC cohort. It should be noted that only 14 genes for the generation of the risk score existed in the Japan-HCC data, so the risk score was calculated by these 14 genes (CDH10, CLDN6, EPO, FCN3, GNGT1, HOXA7, ITGAM, KIF24, MSC, PFN2, SEPT14, TEX15, TTK, and YJEFN3) and their corresponding risk coefficients. After that, patients in the China-HCC and Japan-HCC cohorts were also separated into two groups (high- and low-score groups) according to the optimal cutoff value of the risk score automatically calculated by the “roc” method in the “ggrisk” package (Figure 6A; Supplementary Figure S3A). The results of survival analyses also showed that most of these prognostic genes were highly expressed in the high-score groups (Figure 6A; Supplementary Figure S3A), and patients in the high-score groups had a significantly poorer prognosis than those in the low-score groups (Figure 6B; Supplementary Figure S3B). In addition, potential therapeutic drugs targeting these 19 prognostic genes were preliminarily screened by CellMiner (Supplementary Table S4). After comparing the expression levels of the 19 prognostic genes and the IC_50_ values of drugs that have been approved by the Food and Drug Administration (FDA) or are in clinical trials, the twelve most significant correlation pairs are visualized in Supplementary Figure S4, such as the SR16157–GNGT1 and Imexon–PFN2 pairs. These results suggested that these drugs may help prolong the OS time and response to immunotherapy in HCC patients by targeting the 19 prognostic genes.

**TABLE 1:** Significant prognostic DEGs and their corresponding coefficients.

Gene	Coefficient
AC012512.1	0.64716
CCT7P2	−0.58323
CDH10	0.235,149
CLDN6	0.265357
EPO	0.231876
FCN3	−0.208
GNGT1	−0.54705
HOXA7	0.238912
ITGAM	−0.15749
KIF24	−1.13771
MEP1AP4	0.527339
MSC	0.193459
PFN2	0.126459
RN7SL368P	0.944861
RP1-63M2.5	1.476354
SEPT14	0.35305
TEX15	0.886103
TTK	0.600176
YJEFN3	−0.21253

Finally, to explore the differences in biological characteristics among these two risk groups, GSVA was conducted in the TCGA-HCC cohort. As shown in Figure 5C, the high-score group had significantly different biological characteristics from those of the low-score group, while the most significant terms enriched in the high-score group were consistent with the results of cluster-1 analysis (Figure 3C, Figure 5C). These results revealed that the high-score group had prognostic and functional characteristics similar to those of cluster-1.

### Immune analyses in the high- and low-score groups

**FIGURE 7:**
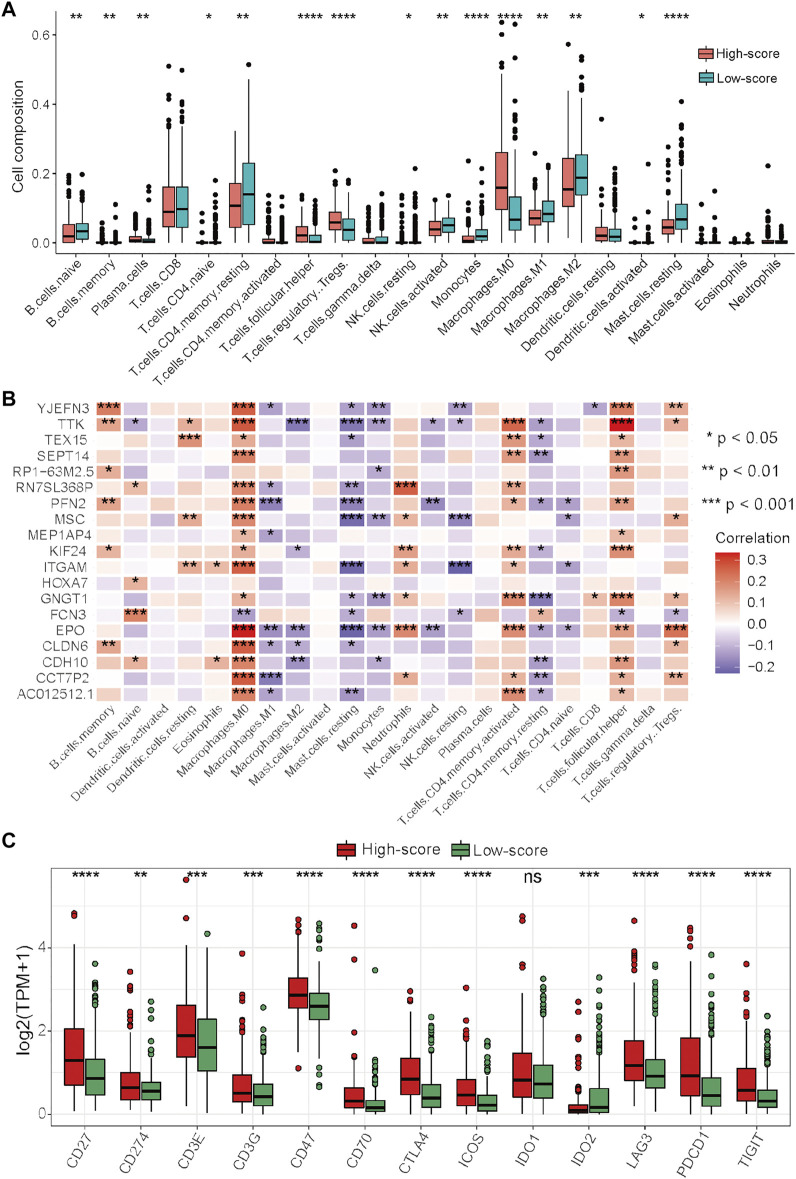
Differential analysis of immune characteristics between the two risk groups in the TCGA–HCC cohort. **(A)** Differences in the 22 immune cell types between the two risk groups. **(B)** Correlations between the 19 prognostic DEGs for the establishment of the risk score and 22 immune cells. **(C)** Differences in the 13 immune checkpoint molecules between the two risk groups. *p < 0.05, **p < 0.01, ***p < 0.001, and ****p < 0.0001.

To further explore the relationship between the risk score and immune characteristics, immune analyses were performed. There were also significant differences in immune characteristics between the high- and low-score groups (Figure 7A). Consistent with the immune characteristics in cluster-1, follicular helper T cells, regulatory T cells, and M0 macrophages were significantly abundant in the high-score group, while monocytes, M1 macrophages, M2 macrophages, and resting mast cells were significantly abundant in the low-score group (Figure 7A). As revealed by the results of Spearman’s correlation analysis, the levels of prognostic DEGs had more significant correlations with the abundance of immune cells than with the levels of cuproptosis-related genes (Figure 7B). We also noticed that the levels of almost all prognostic DEGs were significantly positively associated with the abundances of M0 macrophages and follicular helper T cells, which had significantly different abundances between the high- and low-score groups (Figures 7A,B). Finally, the expression levels of immune checkpoints between the two risk groups were compared. The results showed that most of the immune checkpoint molecules were significantly highly expressed in the high-score group, except for IDO1 and IDO2 (Figure 7C).

Taken together, these results indicated that the high-score group had immune characteristics similar to those of cluster-1, while the prognostic DEGs used for the generation of the risk score had more significant correlations with the abundances of immune cells. In this context, ICIs may be more effective for HCC patients in the high-score group, and targeting these prognostic DEGs may be a potential way to improve the inhibitory immune microenvironment to improve the efficacy of immunotherapy.

### Analysis of the relationship between clinicopathological characteristics and the risk score

**FIGURE 8:**
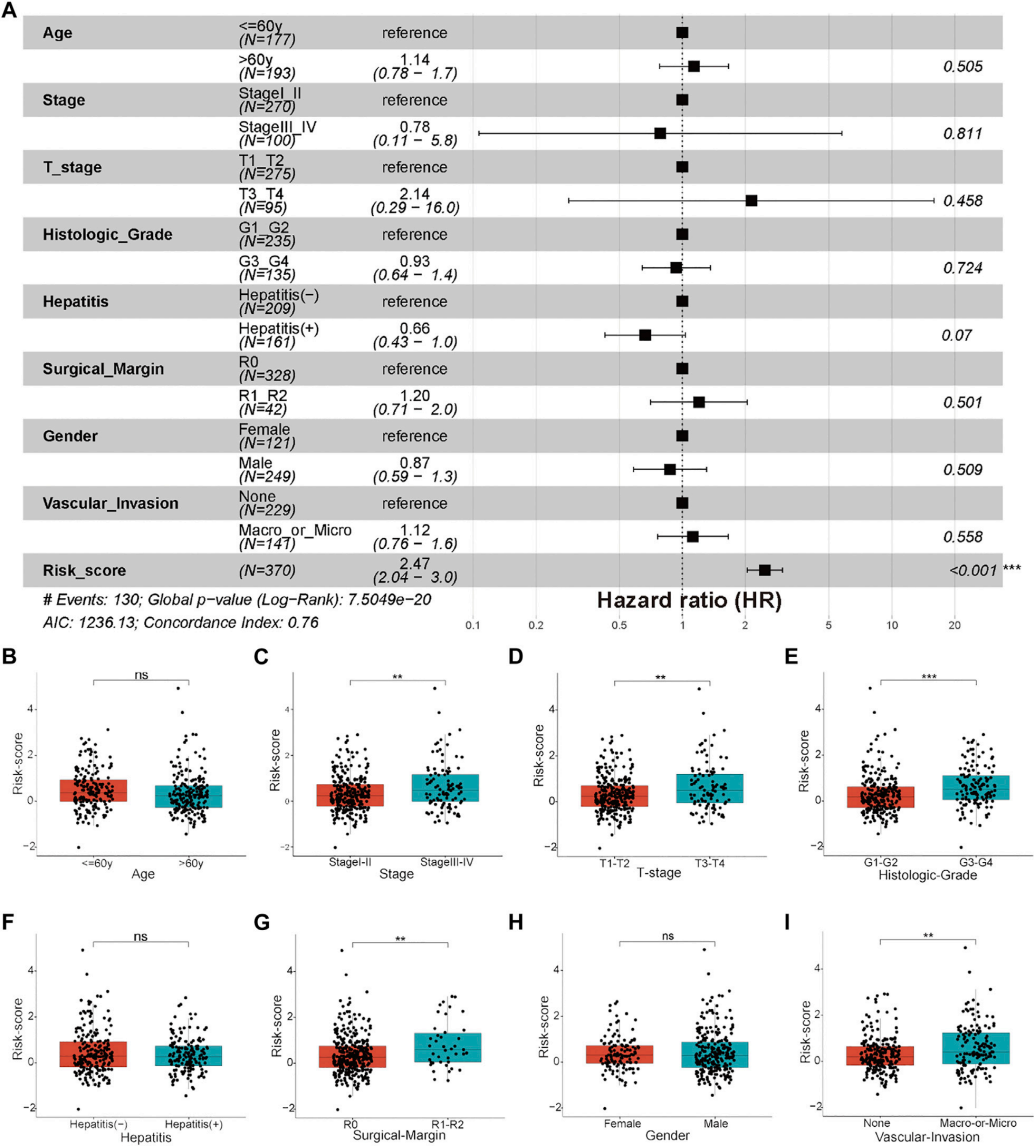
Clinicopathological features and clinical subgroup analysis. **(A)** Forest plot of clinicopathological features and risk scores in the TCGA-HCC cohort. **(B–I)** Boxplot results of the expression levels of the risk score among different clinical subgroups, including **(B)** age, **(C)** stage, **(D)** T stage, **(E)** histological grade, **(F)** hepatitis status, **(G)** surgical margin, **(H)** gender, and **(I)** vascular invasion. *p < 0.05, **p < 0.01, and ***p < 0.001, ns: not significant.

There were no significant differences in clinicopathological characteristics before and after interpolation (Supplementary Figure S5; Supplementary Table S5) in the TCGA-HCC cohort, so we used the interpolated data for follow-up analysis. The results of Cox and subgroup analyses suggested that the risk scores were independent prognostic factors in the training and validation cohorts (Supplementary Tables S6–S8; Figure 6C; Figure 8A; Supplementary Figure S3C), and the patients with a large tumor size (“> 5 cm”) had a worse prognosis than the patients with a small tumor size (“≤ 5 cm”) (Figure 6C; Supplementary Figure S3C). Next, risk scores between the different clinical subgroups were compared in the TCGA-HCC cohort. The results showed that HCC patients with higher stage (“Stage III–IV”), higher T stage (“T3–T4”), higher histological grade (“G3–G4”), nonradical resection (“R1–R2”), and vascular invasion (“Macro–Micro”) had higher risk scores (Figures 8B–I). These results were consistent with clinical experiences that patients with these adverse clinicopathological characteristics have high risk scores.

### Overall expression analyses of the cuproptosis-related genes and prognostic DEGs for the establishment of the risk score through scRNA sequencing data

**FIGURE 9:**
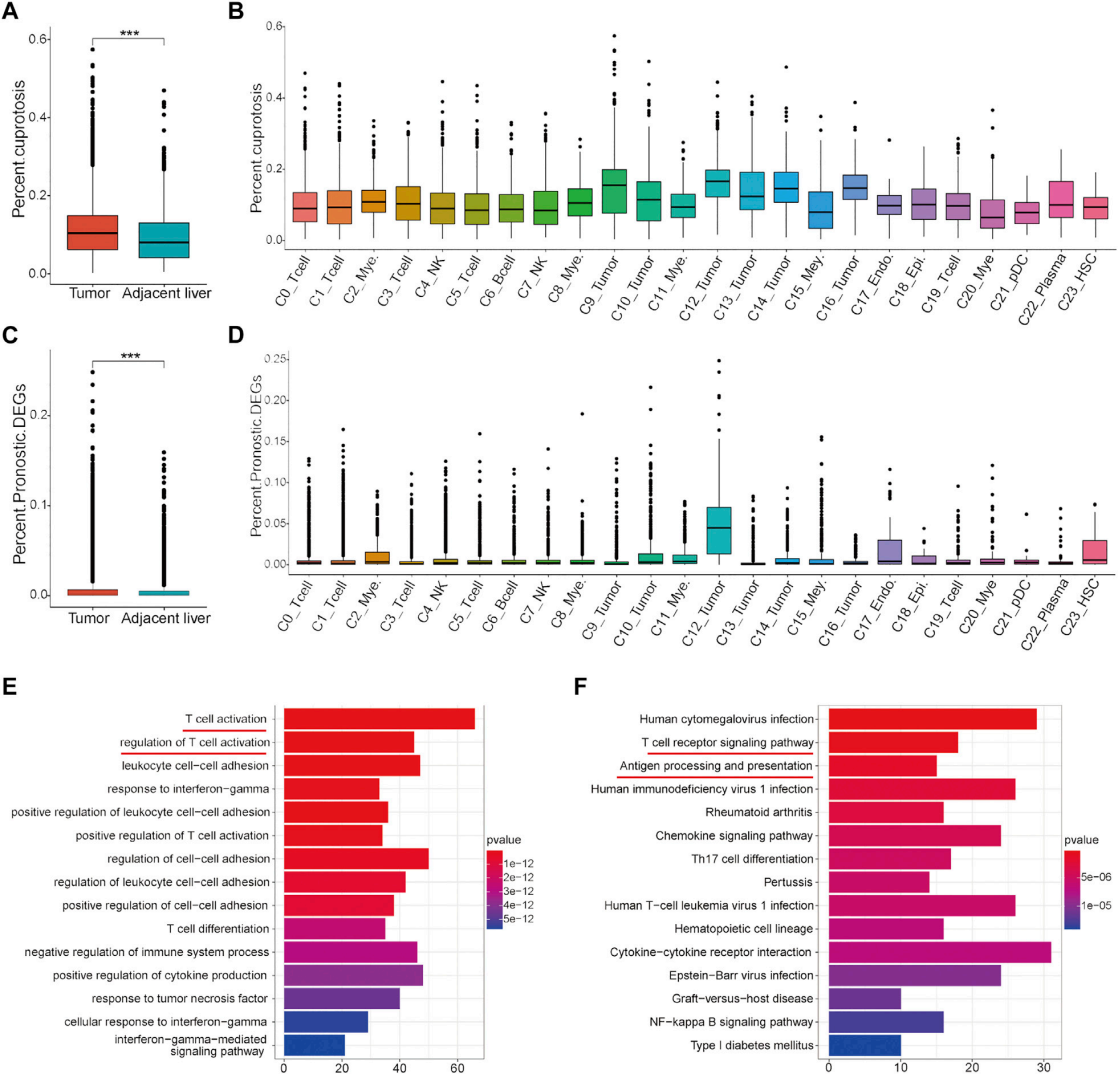
Analysis of the proportion of cell expression two gene sets in the HCC tumor microenvironment by scRNA data. **(A)** Differences in the proportion of cell expression cuproptosis-related genes between HCC tumor tissues and adjacent liver tissues. **(B)** Proportion of cuproptosis-related genes in all cell types. **(C)** Differences in the proportion of cells expressing the prognostic DEGs between HCC tumor tissues and adjacent liver tissues. **(D)** Proportion of cells expressing the prognostic DEGs in all cell types. **(E)** GO analysis results of the significant DEGs in tumor cell cluster-C12. **(F)** KEGG analysis results of the significant DEGs in tumor cell cluster-C12.

To further clarify the overall expression status of these cuproptosis-related genes and prognostic DEGs in each cell cluster, scRNA sequencing data were analyzed. In general, cells expressing these two gene sets had a higher proportion of the total number of cells in HCC tumor tissues than in adjacent liver tissues (Figures 9A,C). In detail, cells expressing cuproptosis-related genes account for a certain proportion of all cell types in the tumor microenvironment, although they account for a higher proportion in some tumor cell clusters (C9, C12, C14, and C16) (Figure 9B). However, the proportion of cell expression prognostic DEGs in almost all cell clusters in the tumor microenvironment was very low, except for in a tumor cell cluster-C12 (Figure 9D). On this condition, significant DEGs between C12 and other cell clusters were identified for subsequent functional enrichment analysis. The results of GO and KEGG analyses showed that tumor cell cluster-C12 was closely associated with T cell activation and the T cell receptor signaling pathway (Figures 9E,F). These results indicated that tumor cells, especially C12, are the main target cell types for subsequent intervention of these cuproptosis-related genes and prognostic DEGs in the HCC immune microenvironment to synergistically improve the efficacy of immunotherapy.

## Discussion

Copper is an important trace metal used to maintain normal life activities, but its excessive high or low expression will cause diseases (Dou et al., 2022; Kahlson and Dixon, 2022). For a long time, the mechanism of copper-induced cell death was unclear until a recent study showed that copper-induced cell death, called cuproptosis, was associated with the disorder of metabolites related to the tricarboxylic acid cycle (Tsvetkov et al., 2022). However, the integrated role of cuproptosis-related genes and their functional characteristics in HCC remain largely unknown.

In this study, a cuproptosis-related gene list was sorted according to the recent literature. The expression status and biological characteristics of these cuproptosis-related genes in HCC were comprehensively analyzed. In detail, based on the 16 cuproptosis-related genes, three distinct expression patterns (cluster-1, cluster-2, and cluster-3) were identified in the TCGA-HCC cohort. These three clusters had significantly distinct prognostic, functional, and immune characteristics. It is noteworthy that functional terms of cluster-1 with poor prognosis were enriched in genetic alterations such as mismatch repair and homologous recombination, as revealed by GSVA. In addition, the results of immune analysis revealed that there was significant enrichment for inhibitory immune cells in cluster-1, such as follicular helper T cells and regulatory T cells, and most of the main immune checkpoint molecules were also expressed at significantly high levels in cluster-1. These results suggest that the functional roles of cuproptosis-related genes in HCC may be partly achieved by causing genetic alterations and immune dysfunction.

To quantify the characteristics of cuproptosis in each HCC patient, a cuproptosis-related risk score was established. As revealed by the results of prognostic and functional analyses, HCC patients in the high-score group had a significantly poorer prognosis and inhibitory immune cell infiltration and as high expression levels of the main immune checkpoint molecules. These results indicated that this risk score system can well-integrate the functional characteristics of cuproptosis-related genes. It has been reported that an inhibitory immune microenvironment is usually related to poor prognosis and a non-sensitive response to immunotherapy (Li et al., 2016; Zhao et al., 2019; Eschweiler et al., 2021; Gao et al., 2022). Therefore, HCC patients with higher cuproptosis-related risk scores may be more responsive to immunotherapy with ICIs due to the high expression levels of immune checkpoint molecules. Targeting these cuproptosis-related genes or prognostic DEGs used for establishment of the risk score may ameliorate the inhibitory immune microenvironment to improve the efficacy of immunotherapy and thus prognosis. In addition, we noticed that there was a significantly increased abundance of unpolarized macrophages (M0 macrophages) in cluster-1 and the high-score group and a decreased abundance of polarized macrophages (M1 macrophages and M2 macrophages). These results indicated that macrophages in the high-score group may have strong plasticity and that inducing M0 macrophages to polarize into M1 macrophages (classic activated macrophages) may also be a potential strategy to improve the efficacy of immunotherapy in HCC patients in the high-score group.

The tumor microenvironment includes a variety of complex cell components, such as immune cells, stromal cells, and tumor cells (Shi et al., 2022; Van den Bossche et al., 2022). The difference in their composition and expression is one of the main reasons for tumor heterogeneity (Srinivasan et al., 2022). Clarifying tumor immune heterogeneity will help identify effective synergistic targets to enhance the efficacy of immunotherapy. In this context, scRNA sequencing data were analyzed in this study to clarify which kind of cells expressed the cuproptosis-related genes and the prognostic DEGs. As revealed by Figure 9, cuproptosis-related genes are expressed to some extent in all cell types of tumor microenvironments, while prognostic DEGs are mainly expressed in tumor cells. These results indicate that cuproptosis-related genes may affect the function of a variety of cells in the immune microenvironment of HCC, resulting in changes in the expression of prognostic DEGs in tumor cells to affect the prognosis of HCC patients and their response to immunotherapy.

There are still some limitations in this study. First, the prognostic and immune predictive value of this cuproptosis-related risk score needs to be validated in more HCC patients from real-world multicenters. Second, whether HCC patients with higher risk scores are more sensitive to ICIs needs to be confirmed by further preclinical and clinical studies. Third, strategies for ameliorating the inhibitory immune microenvironment by targeting these cuproptosis-related genes or prognostic DEGs for establishment of the risk score need to be further researched by experimental and clinical studies.

## Conclusions

In this study, a list of cuproptosis-related genes was identified and comprehensively analyzed in HCC for the first time. Cuproptosis-related genes can be used to identify a specific cluster of HCC patients with poor prognosis and an inhibitory immune microenvironment and may be sensitive to ICI treatment. This cluster of patients could be well-identified by a cuproptosis-related risk score system. In addition, this risk score can be used as an independent prognostic factor for HCC patients. Moreover, the overall expression status of cuproptosis-related genes and the genes for establishing the cuproptosis-related risk score in specific cell types of the tumor microenvironment were preliminarily clarified by scRNA sequencing data. Taken together, these results revealed that cuproptosis-related genes play an important role in HCC, and targeting these genes may ameliorate the inhibitory immune microenvironment to improve the efficacy of immunotherapy with ICIs and thus improve the prognosis of HCC patients.

## Data Availability

The datasets presented in this study can be found in online repositories. The names of the repository/repositories and accession number(s) can be found in the article/Supplementary Material.

## Supplementary material

SUPPLEMENTARY FIGURE S1 Unsupervised clustering and difference analysis in the training cohort. **(A–D)**Consensus matrices for k = 2-5 in the training cohort. **(E)** Delta area plot. **(F)** Consensus CDF plot. **(G)** Differential expression of the 16 cuproptosis-related genes in the three clusters. *P< 0.05, **P< 0.01, ***P<0.001, and ****P<0.0001, ns: not significant.

SUPPLEMENTARY FIGURE S2 Differential analysis and functional enrichment analysis in the training cohort. **(A)** Dot plot of GO analysis results. BP: biological process, CC: cellular components, and MF: molecular function. **(B)** Dot plot of KEGG analysis results. **(C)** Differential expression of the 19 significant prognostic DEGs for the generation of the risk score in the three clusters. **(D)** Differential expression of the risk score in the three clusters.

SUPPLEMENTARY FIGURE S3 External validation of the efficiency of the cuproptosis-related risk score in validation cohort 2 (Japan-HCC). **(A)** Survival status of HCC patients and expression of the 14 significant prognostic DEGs in the high- and low-score groups. **(B)** Kaplan–Meier curve for the two risk groups in the Japan-HCC cohort. **(C)** Forest plot of clinicopathological features and risk scores in the Japan-HCC cohort.

SUPPLEMENTARY FIGURE S4 Pearson correlation analysis between the 19 prognostic genes and drugs. Twelve representative scatter plots of the relationship between drug sensitivity (IC_50_) and the expression levels of the 19 hub genes.

SUPPLEMENTARY FIGURE S5 Comparison of clinicopathological features before and after interpolation. **(A)** Missingness map of the clinical data. **(B)** Graphical illustration of information before and after interpolation. Blue represents before interpolation, and red represents after interpolation.

## Abbreviations

BP, biological process; CC, cellular components; CNVs, copy number variations; DEGs, differentially expressed genes; FDA, Food and Drug Administration; GO, Gene Ontology; GSVA, gene set variation analysis; GTEx, Genotype-Tissue Expression; HCC, hepatocellular carcinoma; HSCs, hepatic stellate cells; ICGC, International Cancer Genome Consortium; KEGG, Kyoto Encyclopedia of Genes and Genomes; MF, molecular function; pDCs, plasmacytoid dendritic cells; RFTA, radiofrequency thermal ablation; scRNA, single-cell RNA; TACE, transcatheter arterial chemoembolization; TCGA, the Cancer Genome Atlas; TKIs, tyrosine kinase inhibitors; TMB, tumor mutation burden; TPM, transcripts per million.
