# Supplementary material for: Comprehensive multiomics analysis of cuproptosis-related gene characteristics in hepatocellular carcinoma
Source: Front Genet. 2022 Sep 6;13:942387. doi: 10.3389/fgene.2022.942387 (PMC9486098; doi:10.3389/fgene.2022.942387)
Supplement: Supplementary file 1 [file Table1.DOCX]

Table S1. CNV status of the 16 cuproptosis-related genes.

| Hugo-Symbol | Amp | Del | total | Altered Samples |
| --- | --- | --- | --- | --- |
| CDKN2A | 0 | 145 | 145 | 145 |
| DLAT | 0 | 94 | 94 | 93 |
| LIAS | 56 | 0 | 56 | 55 |
| SLC31A1 | 0 | 0 | 0 | 0 |
| ATP7A1 | 0 | 0 | 0 | 0 |
| ATP7B | 0 | 0 | 0 | 0 |
| FDX1 | 0 | 0 | 0 | 0 |
| DLD | 0 | 0 | 0 | 0 |
| LIPT1 | 0 | 0 | 0 | 0 |
| LIPT2 | 0 | 0 | 0 | 0 |
| PDHA1 | 0 | 0 | 0 | 0 |
| PDHB | 0 | 0 | 0 | 0 |
| MTF1 | 0 | 0 | 0 | 0 |
| GLS | 0 | 0 | 0 | 0 |
| GCSH | 0 | 0 | 0 | 0 |
| MPC1 | 0 | 0 | 0 | 0 |
