# Supplementary material for: Comprehensive multiomics analysis of cuproptosis-related gene characteristics in hepatocellular carcinoma
Source: Front Genet. 2022 Sep 6;13:942387. doi: 10.3389/fgene.2022.942387 (PMC9486098; doi:10.3389/fgene.2022.942387)
Supplement: Supplementary file 2 [file Table8.DOCX]

Table S8. Univariate and multivariate Cox analyses of the clinicopathological features and risk score in the Japan-HCC cohort.

|  | Univariate Cox analysis | | | Multivariate Cox analysis | | |
| --- | --- | --- | --- | --- | --- | --- |
|  | HR | CI95 | P.Value | HR | CI95 | P.Value |
| Age | 0.75 | 0.33-1.7 | 0.49 | NA | NA | NA |
| Edmondson grade | 2.41 | 1.15-5.07 | 0.02 | 1.26 | 0.57-2.81 | 0.565 |
| Gender | 0.52 | 0.24-1.13 | 0.098 | NA | NA | NA |
| T stage | 2.67 | 1.28-5.56 | 0.009 | 1.52 | 0.68-3.39 | 0.306 |
| Tumor size | 4.07 | 1.95-8.51 | 0 | 3.42 | 1.51-7.73 | 0.003** |
| Risk score | 2.31 | 1.41-3.78 | 0.001 | 2.19 | 1.28-3.74 | 0.004** |

*P< 0.05, **P< 0.01, ***P<0.001.
