# Supplementary figures and images for: Comprehensive multiomics analysis of cuproptosis-related gene characteristics in hepatocellular carcinoma
Source: Front Genet. 2022 Sep 6;13:942387. doi: 10.3389/fgene.2022.942387 (PMC9486098; doi:10.3389/fgene.2022.942387)

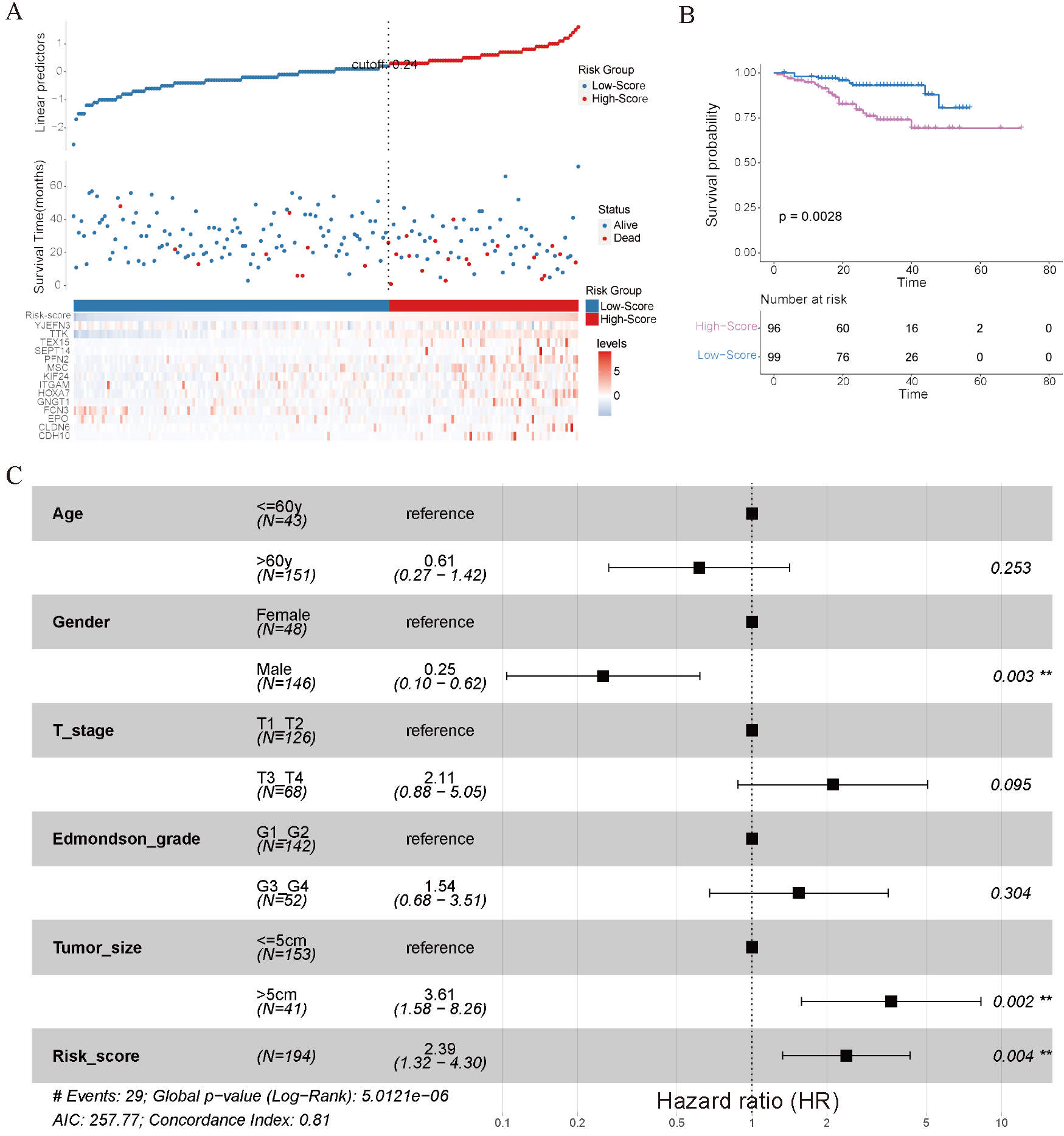

Supplement: Supplementary file 3 [file Image3.TIF]

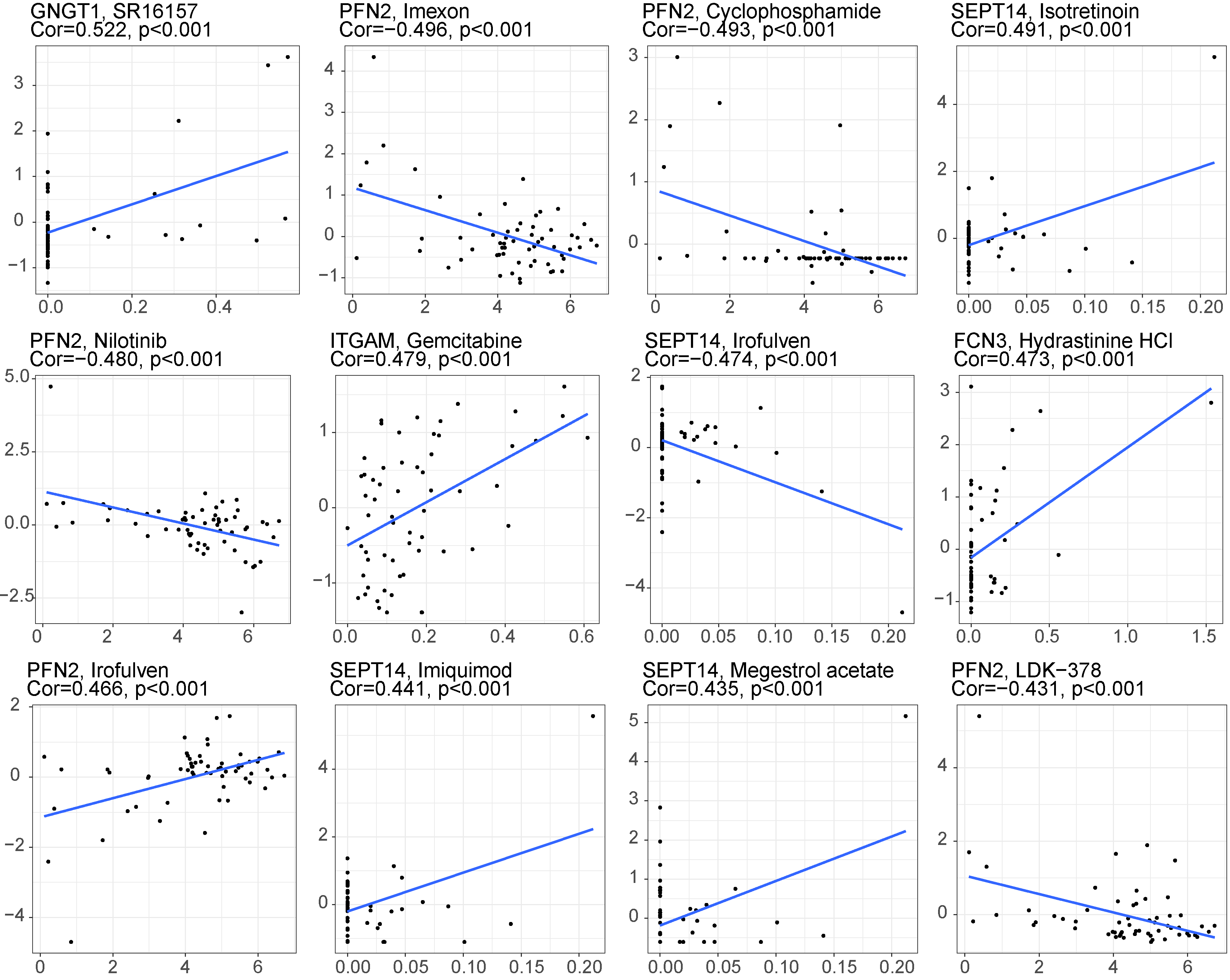

Supplement: Supplementary file 4 [file Image4.TIF]

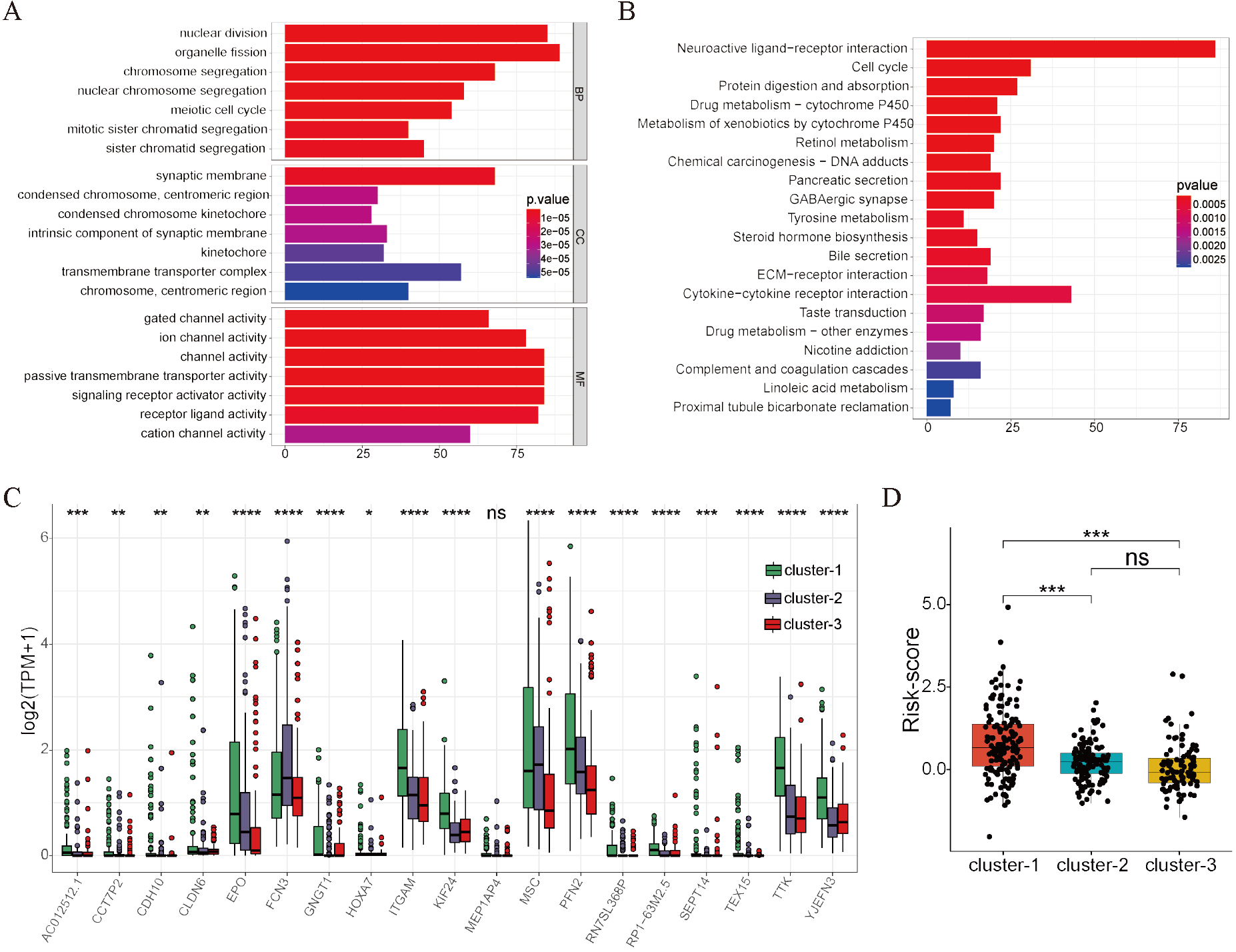

Supplement: Supplementary file 5 [file Image2.TIF]

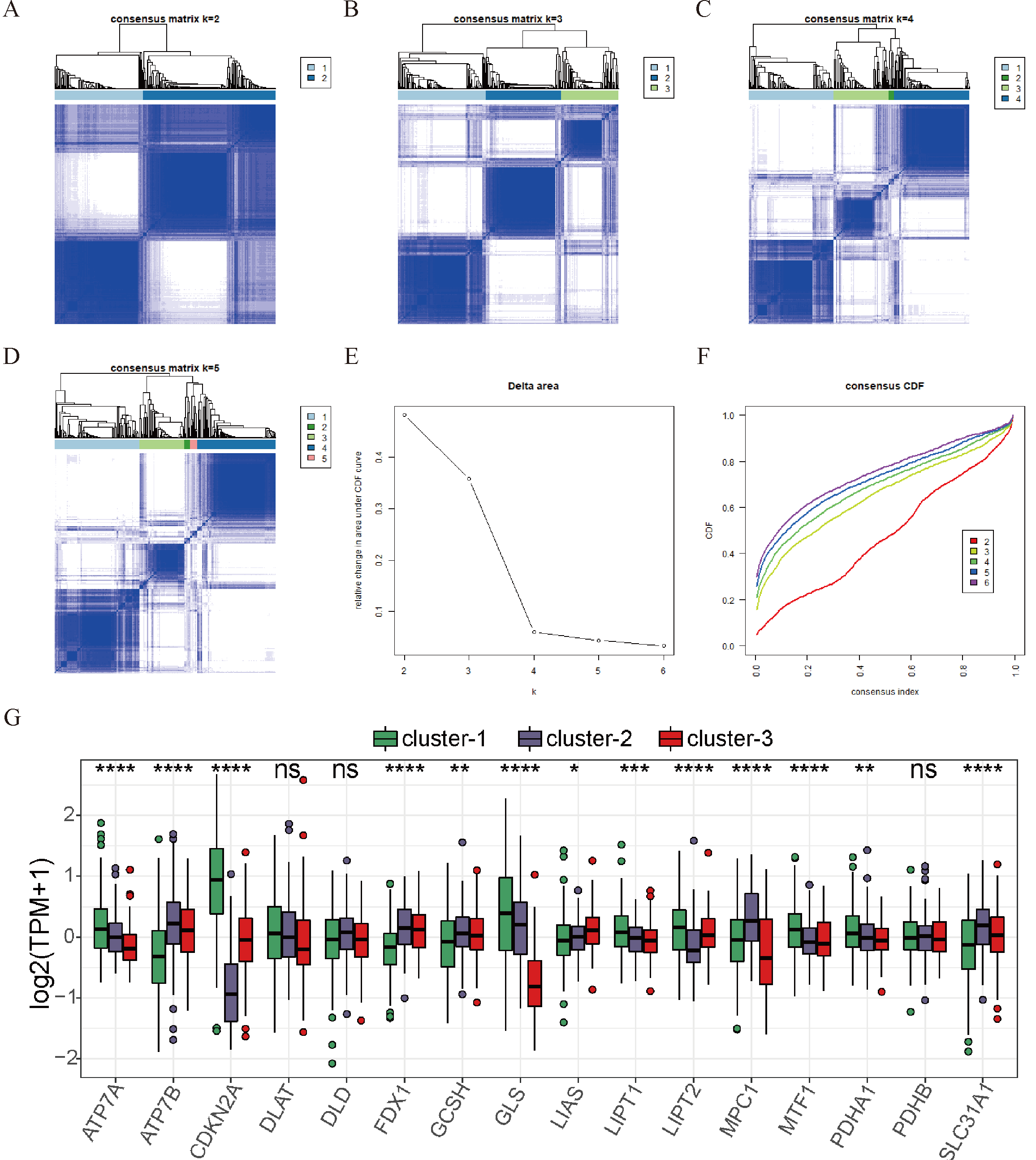

Supplement: Supplementary file 7 [file Image1.TIF]

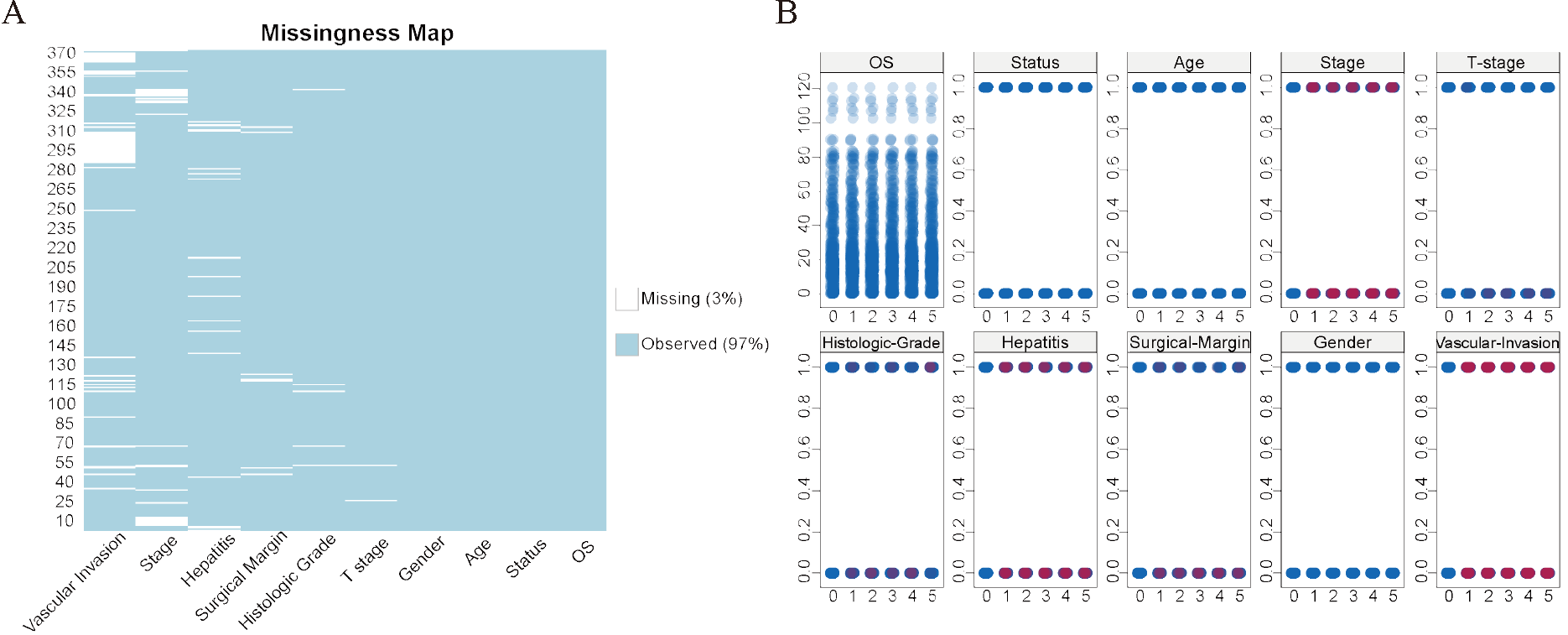

Supplement: Supplementary file 13 [file Image5.TIF]
