# Supplementary material for: Comprehensive multiomics analysis of cuproptosis-related gene characteristics in hepatocellular carcinoma
Source: Front Genet. 2022 Sep 6;13:942387. doi: 10.3389/fgene.2022.942387 (PMC9486098; doi:10.3389/fgene.2022.942387)
Supplement: Supplementary file 6 [file Table7.DOCX]

Table S7. Univariate and multivariate Cox analyses of the clinicopathological features and risk score in the China-HCC cohort.

|  | Univariate Cox analysis | | | Multivariate Cox analysis | | |
| --- | --- | --- | --- | --- | --- | --- |
|  | HR | CI95 | P.Value | HR | CI95 | P.Value |
| Age | 0.8 | 0.44-1.45 | 0.469 | NA | NA | NA |
| Gender | 0.76 | 0.41-1.41 | 0.381 | NA | NA | NA |
| Liver cirrhosis | 1.28 | 0.7-2.35 | 0.422 | NA | NA | NA |
| TNM stage | 1.74 | 1.02-2.95 | 0.041 | 0.96 | 0.53-1.76 | 0.903 |
| Tumor number | 0.8 | 0.43-1.48 | 0.471 | NA | NA | NA |
| Tumor size | 2.83 | 1.58-5.06 | 0 | 2.59 | 1.35-4.98 | 0.004** |
| Risk score | 1.43 | 1.14-1.79 | 0.002 | 1.31 | 1.04-1.65 | 0.024* |

*P< 0.05, **P< 0.01, ***P<0.001.
