# Supplementary material for: Comprehensive multiomics analysis of cuproptosis-related gene characteristics in hepatocellular carcinoma
Source: Front Genet. 2022 Sep 6;13:942387. doi: 10.3389/fgene.2022.942387 (PMC9486098; doi:10.3389/fgene.2022.942387)
Supplement: Supplementary file 8 [file Table2.DOCX]

Table S2. List of the 3854 significant prognostic DEGs.

| CFTR |
| --- |
| MYH16 |
| CYP26B1 |
| ZMYND10 |
| ABCB5 |
| ARX |
| SLC4A1 |
| CALCR |
| PRSS22 |
| HOXA11 |
| WDR54 |
| ABCB4 |
| ITGA3 |
| ITGA2B |
| TMEM132A |
| CACNA1G |
| CCL26 |
| USH1C |
| LGALS14 |
| SCIN |
| MYH13 |
| UPP2 |
| PRSS21 |
| PROM1 |
| CEACAM7 |
| TKTL1 |
| SELE |
| FMO3 |
| NOX1 |
| E2F2 |
| TFAP2B |
| AASS |
| GIPR |
| PRSS3 |
| DPF1 |
| PLAUR |
| ANLN |
| DCN |
| GABRA3 |
| TACC3 |
| ACPP |
| ISL1 |
| IL20RA |
| CLCA1 |
| SLC38A5 |
| CNTN1 |
| MARCO |
| CYP24A1 |
| SYT13 |
| PLEKHB1 |
| CYP3A43 |
| NRXN3 |
| NLRP2 |
| DEPDC1 |
| HSD17B6 |
| IBSP |
| MUSK |
| TMSB10 |
| DEPDC1B |
| OTC |
| C6 |
| CDH10 |
| RAB27B |
| C2orf83 |
| BARX2 |
| ZIC2 |
| EPHA3 |
| DSG2 |
| MAGEC2 |
| TNFRSF17 |
| LMO3 |
| COL9A2 |
| UTS2 |
| TNFRSF9 |
| EPN3 |
| NFE2L3 |
| LAMC3 |
| RAD51 |
| POLQ |
| ALX4 |
| PTPRN |
| CBLN4 |
| MCOLN3 |
| NPFFR2 |
| PHF21B |
| SERPINB3 |
| LAMC2 |
| STYK1 |
| CA11 |
| WISP2 |
| DMRT3 |
| NGFR |
| LPAR2 |
| SNCAIP |
| CHI3L2 |
| CALCRL |
| OAT |
| MCM10 |
| SNAP91 |
| SLC9A3 |
| CLDN18 |
| PFKP |
| PKM |
| SYT1 |
| PRR11 |
| PAGE1 |
| PITX1 |
| PFN2 |
| TRIP13 |
| SLC6A15 |
| SPP2 |
| ASIC4 |
| SPEG |
| HMMR |
| MCM2 |
| SLC12A1 |
| WSCD2 |
| GTSE1 |
| FGF4 |
| CACNG4 |
| WDR62 |
| MCM6 |
| CTTNBP2 |
| UBE2T |
| SPAG6 |
| SMC1B |
| NEBL |
| RBFOX1 |
| TP73 |
| CDH17 |
| DUSP13 |
| PAFAH1B3 |
| AFM |
| EPHA6 |
| SCTR |
| CPB2 |
| NDC80 |
| CDH7 |
| SLC13A1 |
| DLG3 |
| KCNK2 |
| LINC01587 |
| GSTP1 |
| KIF3C |
| TTC39A |
| ORC1 |
| RAD54L |
| HBQ1 |
| CEACAM6 |
| GNAO1 |
| PTHLH |
| SULT2B1 |
| TPX2 |
| FER1L4 |
| DOCK3 |
| TMEM40 |
| SLC4A11 |
| CFAP61 |
| LHX5 |
| CHGB |
| NOS1 |
| FXYD3 |
| BIRC5 |
| LAG3 |
| SI |
| KIF4A |
| DLL3 |
| SLC26A3 |
| SEL1L3 |
| ORC6 |
| CPA1 |
| ESR1 |
| SLC22A17 |
| WDR76 |
| CLSPN |
| UNC13D |
| TGFB2 |
| CDC45 |
| CDC6 |
| TDRD1 |
| IL11 |
| MYO3A |
| TREM2 |
| ITPR3 |
| CDC7 |
| MAGEB2 |
| IGF2-AS |
| P2RX6 |
| PLA2G3 |
| CENPM |
| SEPT3 |
| DMC1 |
| RSPH14 |
| RAB36 |
| TIMP3 |
| PNPLA5 |
| GZMB |
| CDKN3 |
| ISM2 |
| CHGA |
| GALNT16 |
| PAPLN |
| MMP9 |
| GINS1 |
| MYBL2 |
| BIRC7 |
| NKAIN4 |
| COL20A1 |
| SPEF1 |
| SLC52A3 |
| ANGPT4 |
| RSPO4 |
| MYLK2 |
| PAK7 |
| E2F1 |
| WFDC2 |
| SYNDIG1 |
| CELF4 |
| RNF125 |
| VSIG1 |
| F9 |
| PLP2 |
| LUZP4 |
| ELF4 |
| PCSK1N |
| PHEX |
| BRS3 |
| CENPI |
| DRP2 |
| FGF9 |
| SGCG |
| SLC25A15 |
| IRG1 |
| OLFM4 |
| FA2H |
| CRISPLD2 |
| ABCC1 |
| HAGHL |
| PRSS33 |
| PYCARD |
| TMC5 |
| SLC6A2 |
| RHOV |
| OIP5 |
| MYEF2 |
| EYA1 |
| TRPA1 |
| CALB1 |
| TUSC3 |
| DHDH |
| CKM |
| RNASEH2A |
| AMH |
| DKKL1 |
| CLEC4M |
| IL4I1 |
| ASF1B |
| TNNT1 |
| CASP14 |
| CCNE1 |
| OVOL3 |
| SLC1A5 |
| APLP1 |
| CEACAM5 |
| ATP1A3 |
| CNFN |
| SYNGR4 |
| CCDC114 |
| CACNG7 |
| KLF1 |
| KCNN1 |
| MAG |
| HAMP |
| ITGB8 |
| STEAP1B |
| ATP6V0A4 |
| WNT2 |
| HOXA6 |
| ANKRD7 |
| CPED1 |
| EVX1 |
| STX1A |
| EPHB6 |
| NPTX2 |
| NUDT1 |
| HYAL4 |
| TFR2 |
| IMPDH1 |
| EZH2 |
| POU6F2 |
| AGR2 |
| GCK |
| GALNTL5 |
| OGN |
| DNM1 |
| CA9 |
| TYRP1 |
| CXCL12 |
| FGF8 |
| PITX3 |
| SORCS1 |
| UBE2S |
| TRIM16L |
| ALDH3A1 |
| ASIC2 |
| CCL7 |
| PTGES3L-AARSD1 |
| MPP2 |
| FOXN1 |
| CWH43 |
| NMU |
| PF4V1 |
| UCP1 |
| CPZ |
| SLC2A9 |
| NEIL3 |
| NKX3-2 |
| HGFAC |
| NCAPG |
| HPX |
| FOLR1 |
| KIAA1549L |
| SLC1A2 |
| SLC35F2 |
| ELMOD1 |
| CALCA |
| ASIC1 |
| SYT10 |
| SLC6A12 |
| FOXM1 |
| RAD51AP1 |
| GPRC5D |
| SLC38A1 |
| CDCA3 |
| ENO2 |
| SLCO1B3 |
| GYS2 |
| AICDA |
| TULP1 |
| MDGA1 |
| BMP5 |
| PRDM13 |
| HDGFL1 |
| GPLD1 |
| EYA4 |
| SLC17A2 |
| PACRG |
| SMOC2 |
| TTK |
| TBX18 |
| C7 |
| KIF20A |
| CDH9 |
| HAVCR1 |
| CDH6 |
| LMNB1 |
| IL4 |
| LIFR |
| C9 |
| HRH2 |
| EHHADH |
| SERPINI2 |
| PFKFB4 |
| ECT2 |
| HHLA2 |
| C3orf52 |
| VIPR1 |
| SLC4A3 |
| IL1A |
| CENPA |
| ITGB6 |
| TLX2 |
| GALNT3 |
| OTX1 |
| IL1RL1 |
| DLX2 |
| KISS1R |
| PAPPA2 |
| NPHS2 |
| CHD5 |
| DLGAP3 |
| LEPR |
| RGS2 |
| CTH |
| KMO |
| KCNQ4 |
| ST6GALNAC5 |
| SLC2A1 |
| CDC20 |
| LPPR5 |
| STMN1 |
| NEK2 |
| CENPF |
| MUC5B |
| TNP1 |
| CASC1 |
| ELOVL4 |
| CNR1 |
| ADGB |
| MYB |
| GHRH |
| CASQ2 |
| TRPM6 |
| TRIM67 |
| WDR34 |
| PHF19 |
| SLC46A2 |
| BCL11A |
| ELOVL3 |
| NKX2-3 |
| HELLS |
| HOXB8 |
| CRHR1 |
| TEK |
| INSL6 |
| GRIA2 |
| TEX11 |
| SOHLH2 |
| DUSP4 |
| ADRA1A |
| NPPB |
| TBX4 |
| NCAPH |
| PLBD1 |
| RP11-408E5.4 |
| KIF18A |
| GJA3 |
| BAI2 |
| TMEM54 |
| HPCA |
| GPSM2 |
| RASL11A |
| OBP2A |
| PLG |
| HOXA7 |
| SPINK4 |
| TAF1L |
| NUDT10 |
| ZWINT |
| CIT |
| CDKN2C |
| BHLHE41 |
| NECAB1 |
| ATP7B |
| CENPK |
| ITIH5 |
| NR4A1 |
| STIL |
| HJURP |
| IL13RA2 |
| SERPINA7 |
| H2BFWT |
| NRK |
| TNFAIP6 |
| C4BPA |
| CKS2 |
| DAW1 |
| SLC12A5 |
| MATN4 |
| GDAP1L1 |
| RBPJL |
| PCK1 |
| MAGEA10 |
| POF1B |
| NDP |
| CRISP2 |
| GRM4 |
| OR2B6 |
| TREM1 |
| MT1G |
| SOX21 |
| C17orf53 |
| KIF25 |
| HS3ST3B1 |
| IL37 |
| CD70 |
| FOSB |
| FAM182A |
| CST8 |
| CSTL1 |
| NRSN2 |
| OVOL2 |
| LAMP5 |
| BPIFB1 |
| PROZ |
| GPR42 |
| KIRREL2 |
| FFAR2 |
| ASL |
| PRKCG |
| DACH2 |
| SSX1 |
| DLGAP5 |
| CTAG2 |
| TMEM35 |
| NXF5 |
| RGS13 |
| OMD |
| MASP1 |
| BEST3 |
| PTPRB |
| TRPV5 |
| AUNIP |
| OR7C1 |
| PKMYT1 |
| CHTF18 |
| GNG13 |
| GNGT1 |
| STEAP4 |
| KDR |
| GAL3ST1 |
| RFPL1 |
| APOL5 |
| LIF |
| RIBC2 |
| KRT17 |
| VGF |
| STRIP2 |
| FEZF1 |
| GAD1 |
| HOXD11 |
| HOXD13 |
| E2F8 |
| FAM64A |
| ATP1B2 |
| AP1M2 |
| EGLN3 |
| FOXJ1 |
| AANAT |
| SGOL1 |
| INS-IGF2 |
| NXNL2 |
| STK33 |
| EPO |
| CACNG6 |
| FCHO1 |
| SULT4A1 |
| CYP2E1 |
| RBBP8NL |
| FIBCD1 |
| NPAS1 |
| SMPDL3B |
| SLC6A8 |
| PNCK |
| SLC7A10 |
| BPIFA2 |
| TEX101 |
| GINS2 |
| GALNT15 |
| PSMC3IP |
| G6PC |
| KREMEN2 |
| BARX1 |
| RHOXF2 |
| TOP2A |
| PPP1R1B |
| LIN28A |
| RTBDN |
| MATN3 |
| SLC6A11 |
| FCRLA |
| SLX1A |
| POPDC3 |
| FTHL17 |
| ITGB4 |
| ATP1A4 |
| RAB25 |
| ALDH3B2 |
| DMGDH |
| SYT4 |
| SLC14A2 |
| PDE6A |
| ATP8A2 |
| MTUS2 |
| RNF17 |
| CHIT1 |
| LGALS12 |
| HRASLS2 |
| ZDHHC8P1 |
| NTS |
| LRRIQ1 |
| TEX15 |
| PEBP4 |
| CCNB1 |
| CHL1 |
| GSTM1 |
| REG4 |
| CHIA |
| PSRC1 |
| TRIM45 |
| VTCN1 |
| SAA2 |
| CFHR4 |
| CFHR5 |
| GRP |
| SLCO1B1 |
| FOLH1B |
| MTNR1B |
| SPOCD1 |
| CDCA8 |
| DSG1 |
| DSC3 |
| FHOD3 |
| TCN1 |
| CLDN10 |
| ACRV1 |
| CCNJL |
| HRK |
| RNFT2 |
| UGT2B28 |
| HILPDA |
| KCP |
| BAI3 |
| KHDC1 |
| EPHA7 |
| CGA |
| PRPH |
| GLS2 |
| KRT85 |
| TROAP |
| PKIB |
| KCNMB4 |
| CHST5 |
| PAX3 |
| EDAR |
| STAB2 |
| SCRN1 |
| IGF2BP3 |
| NKX2-8 |
| MYCBPAP |
| TBR1 |
| GALNT5 |
| SCN7A |
| GAD2 |
| WDR38 |
| GABBR2 |
| CTSV |
| CCL21 |
| DMRT1 |
| ARHGEF39 |
| IGFBPL1 |
| TFAP2A |
| TINAG |
| KIAA0319 |
| TCF19 |
| CLPS |
| NRM |
| TTC29 |
| TTPA |
| TMPRSS4 |
| MMP7 |
| MMP13 |
| TMPRSS13 |
| CASP5 |
| UNC13C |
| NUSAP1 |
| KIF23 |
| PAQR5 |
| STRA6 |
| BRDT |
| SLC44A5 |
| SULT6B1 |
| SIX3 |
| TRIM54 |
| CYP2C9 |
| CYP2C8 |
| LBX1 |
| KIF11 |
| CEP55 |
| OIT3 |
| AOX1 |
| CHRNA1 |
| MNS1 |
| HCN4 |
| NDST4 |
| PRKG2 |
| FGF5 |
| TRPC3 |
| ANXA3 |
| CENPE |
| B4GALNT3 |
| TMEM132B |
| GPR84 |
| SRRM4 |
| CDH24 |
| MDGA2 |
| SYT16 |
| FBLN5 |
| SLC25A47 |
| DUOXA2 |
| DUOX2 |
| FGF7 |
| GCNT3 |
| PIF1 |
| CYP1A1 |
| PCSK6 |
| CCDC33 |
| CYP1A2 |
| RHCG |
| FANCI |
| WDR93 |
| TICRR |
| SEPT12 |
| SLC5A2 |
| CHST4 |
| CTRL |
| ABCA8 |
| C17orf64 |
| SLMO1 |
| SLC13A5 |
| SLC16A3 |
| CARD14 |
| TNFRSF11A |
| CBLN2 |
| PMAIP1 |
| P3H4 |
| PNMT |
| STAC2 |
| FKBP10 |
| SLC2A5 |
| CNKSR1 |
| C1orf94 |
| PLK4 |
| FCN3 |
| CELA3A |
| KIF2C |
| TMEM61 |
| IGSF3 |
| XCL1 |
| MAEL |
| DPT |
| PVRL4 |
| NUF2 |
| CRABP2 |
| SYT14 |
| DTL |
| HHIPL2 |
| MBOAT2 |
| PPFIA4 |
| GDF7 |
| REG3G |
| NT5DC4 |
| GPR17 |
| SCN1A |
| CDCA7 |
| DLX1 |
| GULP1 |
| ABCA12 |
| NYAP2 |
| CPNE9 |
| FANCD2 |
| STAC |
| LRTM1 |
| TAGLN3 |
| IGSF11 |
| NCEH1 |
| SPATA16 |
| UCN2 |
| AC062028.1 |
| STXBP5L |
| TM4SF19 |
| VWA5B2 |
| EPHA5 |
| SLC10A6 |
| GLRA3 |
| CDH18 |
| FAM159B |
| HAPLN1 |
| CRHBP |
| CXCL14 |
| GFRA3 |
| HIST1H2BA |
| HMGCLL1 |
| CYP39A1 |
| TPBG |
| FAXC |
| GABRR1 |
| CLVS2 |
| RSPO3 |
| MTFR2 |
| VWDE |
| CDCA5 |
| TRIM50 |
| ZNF157 |
| NXF3 |
| CXorf57 |
| HTR2C |
| ARHGAP36 |
| GPR119 |
| FATE1 |
| MAGEA4 |
| CHRNB3 |
| ST18 |
| RGS20 |
| CRH |
| LRP12 |
| GSDMC |
| VLDLR |
| CER1 |
| CDKN2B |
| CDKN2A |
| CRB2 |
| SLC25A25 |
| CACNA1B |
| ST8SIA6 |
| ANKRD30A |
| VAX1 |
| PLEKHS1 |
| MKI67 |
| NKX6-2 |
| SYT8 |
| ZNF215 |
| DRD2 |
| HTR3B |
| GLB1L2 |
| ZP1 |
| CHEK1 |
| FAM57B |
| MMP3 |
| CNTN5 |
| PCDH15 |
| FCGR1A |
| LYPD6B |
| GPM6A |
| CNDP1 |
| C11orf53 |
| C2orf50 |
| TKTL2 |
| CENPU |
| CCDC74B |
| GRID2 |
| SPC25 |
| BOLL |
| GRIA4 |
| DMP1 |
| MEPE |
| CAPSL |
| DDX4 |
| ANKRD22 |
| BMP3 |
| GRM1 |
| CNTNAP4 |
| PART1 |
| LMNTD1 |
| CENPH |
| GPR115 |
| FRMD1 |
| FAM81B |
| PLEKHG4B |
| TGIF2LX |
| FAM92B |
| TMPRSS11D |
| KCNJ16 |
| AK5 |
| CABYR |
| C16orf74 |
| PANX3 |
| CDH12 |
| ABCA9 |
| UCHL1 |
| ASZ1 |
| FAM27C |
| PSMA8 |
| CHODL |
| TMPRSS15 |
| SLFN13 |
| SKA1 |
| EME1 |
| SEPT14 |
| CNTNAP5 |
| PTPRN2 |
| GOLGA7B |
| MAGEC1 |
| XAGE2B |
| SPAG17 |
| FMN2 |
| SLC24A2 |
| RAET1L |
| AFF2 |
| GRIP1 |
| NAT2 |
| MAGEA8 |
| WIF1 |
| GPR61 |
| NAA11 |
| FBXO43 |
| UNC5D |
| GLYATL2 |
| BUB1B |
| SST |
| LRP8 |
| C1orf158 |
| CLEC18C |
| CCNB2 |
| FAM81A |
| MUM1L1 |
| CREB3L1 |
| SLC34A2 |
| DPYSL5 |
| SSX8 |
| EXTL1 |
| TRIM63 |
| HPD |
| FAM46B |
| RHBDL2 |
| CDC25C |
| TMSB15B |
| CPA2 |
| CPA5 |
| PAGE5 |
| NBL1 |
| VWA5B1 |
| WNT9B |
| HOXB13 |
| GIP |
| CBR3 |
| CHAF1B |
| GOLGA6A |
| PLA2G4D |
| IRX6 |
| ISL2 |
| UROC1 |
| LRRC36 |
| PIP |
| TFF2 |
| TFF1 |
| G6PD |
| ZNF208 |
| FCN2 |
| LCN1 |
| COX6B2 |
| TMEM190 |
| NLRP4 |
| CYP3A4 |
| CYP3A7 |
| LY6K |
| RECQL4 |
| FOXH1 |
| DMKN |
| NPHS1 |
| CCDC155 |
| HCRT |
| MPP3 |
| CD300LG |
| PLCD3 |
| RACGAP1 |
| SPC24 |
| CCDC78 |
| C16orf59 |
| CCNF |
| PAQR4 |
| ZG16B |
| ASRGL1 |
| CYP4A22 |
| TAL1 |
| FAM151A |
| LHX8 |
| B3GALT2 |
| WDR63 |
| HFM1 |
| BRINP3 |
| VANGL2 |
| FCGR3B |
| AXDND1 |
| TDRD5 |
| SPATA17 |
| C1orf145 |
| C2orf48 |
| CCDC74A |
| SLC16A14 |
| SGPP2 |
| XIRP2 |
| PDHA2 |
| S100A11 |
| BMP10 |
| PGLYRP4 |
| S100A9 |
| ALPPL2 |
| ALPI |
| DAPL1 |
| LINC01116 |
| TRIM46 |
| ADORA1 |
| KIAA1524 |
| SGOL2 |
| SERPINI1 |
| AIM2 |
| SLC2A2 |
| CADPS |
| NKX6-1 |
| DNASE1L3 |
| CXCL3 |
| CXCL5 |
| CXCL1 |
| PLSCR2 |
| KIF15 |
| CDCP1 |
| LIPH |
| MFI2 |
| SLC9B2 |
| CDC25A |
| MST1R |
| PITX2 |
| PRSS12 |
| NDST3 |
| HAND2 |
| MAD2L1 |
| TMEM155 |
| HPGD |
| NPY1R |
| NPY5R |
| HHIP |
| ELOVL7 |
| RANBP3L |
| ANKRD33B |
| PRDM9 |
| CAGE1 |
| TERT |
| GPR111 |
| ACSL6 |
| IL3 |
| GJB7 |
| GRIK2 |
| FABP7 |
| PI16 |
| TBX20 |
| PTTG1 |
| BMPER |
| KCNK5 |
| CDCA7L |
| FABP5 |
| SUN3 |
| SLC30A8 |
| SBSPON |
| KCNV1 |
| CSMD3 |
| OSR2 |
| BAALC |
| FREM1 |
| DIRAS2 |
| LETM2 |
| NKX6-3 |
| C8orf34 |
| SVEP1 |
| TRPV6 |
| RNF183 |
| ASB11 |
| SLITRK5 |
| MELK |
| ARMC3 |
| LRFN5 |
| MBL2 |
| HEPACAM |
| SKA3 |
| DDIAS |
| RPL10L |
| MAGEC3 |
| NGB |
| CDX2 |
| SSX5 |
| SSX3 |
| DRGX |
| SOHLH1 |
| CLEC1B |
| TMEM52B |
| PPP1R36 |
| BTNL9 |
| ANKRD2 |
| E2F7 |
| OTOGL |
| SMCO2 |
| PDZRN4 |
| NELL1 |
| PTPLA |
| PASD1 |
| TMCO5A |
| GLB1L3 |
| PPP1R14D |
| SERPINB7 |
| LMO1 |
| PKD1L2 |
| MCM7 |
| IQCD |
| MC4R |
| MMP10 |
| HTR3A |
| STRCP1 |
| SAA3P |
| KIAA0101 |
| PLIN1 |
| GLYATL1 |
| C18orf54 |
| PLK1 |
| GPR182 |
| TAC3 |
| CHP2 |
| ELFN2 |
| C15orf48 |
| MS4A8 |
| MS4A15 |
| C15orf43 |
| PIP5KL1 |
| BPIFB6 |
| LINC00483 |
| IGF2 |
| RBFOX3 |
| AC104389.28 |
| LPO |
| CA4 |
| JSRP1 |
| CDT1 |
| RHEBL1 |
| TTYH1 |
| NLRP7 |
| C19orf33 |
| GGT6 |
| KLK1 |
| KLK4 |
| KLK11 |
| KRT80 |
| KRT1 |
| ACER1 |
| RCOR2 |
| TK1 |
| ENTPD3 |
| SCARA5 |
| OR2W6P |
| FAM83B |
| PTF1A |
| FOXI1 |
| HIST1H1E |
| C8orf22 |
| LINC00917 |
| MTNR1A |
| KLHL30 |
| TNXB |
| ADAM18 |
| GDNF |
| UGT3A2 |
| NPNT |
| C20orf62 |
| FAM178B |
| TSPY2 |
| SHOX2 |
| CTRB1 |
| CTRB2 |
| TM4SF20 |
| GRM5 |
| VCX3A |
| ZBBX |
| ACTBL2 |
| IL13 |
| GCSAML |
| HSPB3 |
| NR0B1 |
| STK32A |
| GP2 |
| RNASE2 |
| KCNK9 |
| COL22A1 |
| ZNF280A |
| MUC15 |
| CT55 |
| CLIC3 |
| CKAP2L |
| PROKR1 |
| BCRP2 |
| BUB1 |
| CHRNA5 |
| NLGN1 |
| TACR3 |
| ITGAM |
| PYDC1 |
| TM4SF1 |
| TMEM154 |
| FAM153A |
| VGLL2 |
| FABP6 |
| MRAP |
| C7orf33 |
| CNGB3 |
| SLN |
| CDK1 |
| FRMPD2 |
| CST5 |
| CST1 |
| SP7 |
| SEMA3E |
| ZNF804A |
| ADORA2B |
| MZB1 |
| COX7B2 |
| IRX2 |
| SIX2 |
| GTSF1 |
| ZNF296 |
| HOXB9 |
| TTLL6 |
| SYT9 |
| SDR16C5 |
| LILRP2 |
| TRH |
| PLAC1 |
| PKIA |
| C8orf74 |
| FUT3 |
| KCNG3 |
| RLN3 |
| CSN3 |
| SHCBP1 |
| NPTX1 |
| ESCO2 |
| KRT19 |
| KRT15 |
| XAGE5 |
| KRT20 |
| KSR2 |
| KRT27 |
| CDK5R2 |
| SSX6 |
| NLRP5 |
| MROH2B |
| COL24A1 |
| ETFDH |
| OTP |
| ECEL1 |
| P2RY6 |
| C1orf111 |
| UTF1 |
| CFAP46 |
| MLLT3 |
| RRM2 |
| FRMD5 |
| ZNF556 |
| GAP43 |
| EPHX4 |
| TEX37 |
| CALB2 |
| PRL |
| STARD5 |
| PDZD3 |
| ARNT2 |
| PRSS30P |
| FUT9 |
| ACOT12 |
| SNTG2 |
| MS4A10 |
| CCL19 |
| COL6A5 |
| OVOL1 |
| AQPEP |
| MYEOV |
| XKR3 |
| RXFP4 |
| ADAMTS20 |
| C11orf86 |
| LIPM |
| DMRT2 |
| KCNK7 |
| OLR1 |
| INSM1 |
| SAA1 |
| AGR3 |
| C2orf70 |
| SULT1B1 |
| GPRC6A |
| CMB9-22P13.1 |
| CD7 |
| CCDC13-AS1 |
| TIGD3 |
| CBX2 |
| PIFO |
| SPERT |
| TLR10 |
| SLC16A11 |
| CHRNA9 |
| PODN |
| EXO1 |
| GOLGA6L2 |
| LINGO2 |
| SLC26A9 |
| MYO1H |
| KLK15 |
| IL20RB |
| TRAM1L1 |
| UGT8 |
| KY |
| SLCO2A1 |
| PCP2 |
| CNIH2 |
| NLRP6 |
| PQLC2L |
| CD164L2 |
| UBE2C |
| TMEM51-AS1 |
| CADM2 |
| FAM182B |
| CATSPER1 |
| GRAMD2 |
| CHRNA7 |
| PCSK1 |
| RMI2 |
| TEX26 |
| GPR156 |
| LINC01106 |
| PRIMA1 |
| SFN |
| WBSCR28 |
| A2M |
| DOK7 |
| LRRN1 |
| TMPRSS7 |
| FOXG1 |
| SPHK1 |
| CIDEA |
| EID2B |
| RIMS2 |
| PRR15 |
| DCAF4L2 |
| CNBD1 |
| KBTBD11 |
| C20orf197 |
| MAGEB6 |
| TCERG1L |
| MIR7-3HG |
| IRX5 |
| GRIN1 |
| SOX11 |
| TYMS |
| TYMSOS |
| LY6H |
| CETN1 |
| RIMKLA |
| TRIM72 |
| KCNA2 |
| RP11-551L14.1 |
| VCX2 |
| ST8SIA3 |
| RPRM |
| NHLH2 |
| GSG2 |
| MAGEB10 |
| ANXA2R |
| KCNJ10 |
| AC108142.1 |
| MAMDC4 |
| LCN15 |
| PPP1R42 |
| RP1-232L22__B.1 |
| SLITRK1 |
| SHISA3 |
| KIAA1731NL |
| TUBAL3 |
| UCN3 |
| CTXN1 |
| C5orf46 |
| GDPD4 |
| RNF186 |
| MSC |
| LGALS7B |
| AURKB |
| ZFP42 |
| TAAR3 |
| FAM133A |
| HTR1F |
| TMTC2 |
| HES7 |
| ALOXE3 |
| DAND5 |
| MKRN3 |
| ALOX12B |
| ALOX15B |
| RPRML |
| ARL14 |
| APOBEC3B |
| ATP8B5P |
| OR7E22P |
| MRGPRX4 |
| MRGPRX3 |
| ZNF648 |
| LINC00652 |
| C10orf91 |
| FAR2P1 |
| SLC9A4 |
| GPR144 |
| PLD5 |
| ALX1 |
| FZD2 |
| EMC3-AS1 |
| TPRXL |
| LINC01559 |
| OXTR |
| OR52N2 |
| CHRM2 |
| MAPK15 |
| HLA-V |
| OFCC1 |
| CCL13 |
| UTS2R |
| DDN |
| SAGE1 |
| MAB21L2 |
| FANCB |
| C6orf223 |
| FDCSP |
| PHLDA2 |
| GPR88 |
| C5orf30 |
| TMEM252 |
| CELF2-AS1 |
| USH1G |
| KCNIP1 |
| FAM159A |
| LDOC1 |
| FAM153B |
| BACE2 |
| GABRG3 |
| TMIGD1 |
| NXPH4 |
| CLEC4G |
| VCX |
| HS3ST4 |
| CCNYL2 |
| KCNB2 |
| PPP1R27 |
| MAGEB17 |
| WFDC10B |
| OTOP3 |
| MCEMP1 |
| PCP4 |
| ABAT |
| NKX2-5 |
| CALHM3 |
| RIPPLY3 |
| GPR19 |
| RP11-863K10.7 |
| C2CD4C |
| CHST6 |
| POTEC |
| CTNNA3 |
| CCBE1 |
| EPHA10 |
| IZUMO1R |
| CCR3 |
| GPR1 |
| EFHC2 |
| MRGPRX2 |
| NPBWR1 |
| ASCL2 |
| MACC1 |
| TRAIP |
| FOXL2 |
| B3GALT5 |
| KCTD8 |
| ZNF730 |
| ARSI |
| C1orf64 |
| KCNH8 |
| DSCR4 |
| KRTAP20-2 |
| TREML3P |
| RPL7AP28 |
| KCNQ3 |
| DGAT2L6 |
| CDR1 |
| KCNK12 |
| EFNA5 |
| KRTAP19-1 |
| HIST1H1B |
| PKP3 |
| COLEC10 |
| KNTC1 |
| OR56A3 |
| POU3F2 |
| SLITRK6 |
| PIWIL3 |
| KCNH7 |
| ZNF93 |
| CDCA2 |
| CLDN6 |
| DDX53 |
| TUSC5 |
| LINC00308 |
| TCEAL2 |
| CLCNKB |
| FAM227A |
| MUC6 |
| RFX6 |
| SGCZ |
| FAM169B |
| ANO9 |
| MYADML2 |
| INPP5J |
| LINC00313 |
| MAGEA11 |
| WBSCR17 |
| CTD-2139B15.1 |
| C12orf56 |
| C14orf80 |
| LINC00158 |
| MUC1 |
| AHNAK2 |
| PDIA2 |
| KRT79 |
| SMIM23 |
| PRAME |
| NRG3 |
| SRL |
| IFIT1 |
| FFAR3 |
| LINC00839 |
| CALHM1 |
| NKAIN3 |
| LEMD1 |
| AC084219.4 |
| HTR3E |
| NBPF6 |
| LRRC70 |
| AC013271.3 |
| KIF18B |
| SAPCD2 |
| GABRA5 |
| RGS9BP |
| SLC36A2 |
| TRDN |
| SPATA12 |
| DEFB132 |
| PCLO |
| ANKRD20A5P |
| MYT1L |
| C5orf38 |
| HPDL |
| KIF24 |
| PDE2A |
| MPPED1 |
| FOXI2 |
| FOXE3 |
| KRT16 |
| QRFPR |
| ERCC6L |
| TNFRSF18 |
| C1QL4 |
| SERPINA11 |
| P2RY4 |
| FAM183A |
| KRTAP19-5 |
| PNLIPRP1 |
| LYPD6 |
| H1FNT |
| GCNT1 |
| KRT12 |
| NPSR1 |
| DCC |
| LHFPL3 |
| CDHR4 |
| NANOS3 |
| COX8C |
| TMEM8C |
| TCL6 |
| ERC2 |
| AMTN |
| CXorf67 |
| KBTBD12 |
| DNAJB13 |
| ADH1A |
| LIN28B |
| TMEM72 |
| FAM205CP |
| P2RX2 |
| DMBT1 |
| COL14A1 |
| DNER |
| S100A3 |
| NWD1 |
| RAB42 |
| WNT7B |
| MESP2 |
| COL4A5 |
| OTOG |
| HEPACAM2 |
| PLA2G2A |
| SBK1 |
| PRR19 |
| PDCD1 |
| SERPINA5 |
| NCCRP1 |
| COL25A1 |
| FAM72B |
| ASAH2 |
| FSIP2 |
| BCL2L15 |
| HMX2 |
| GJB3 |
| NYX |
| UTS2B |
| KIR2DL4 |
| FAM111B |
| GAGE2A |
| CLDN4 |
| GRAPL |
| HN1 |
| ZAR1L |
| S100A13 |
| DCAF8L2 |
| DPY19L2P1 |
| AC069277.2 |
| C6orf222 |
| SH2D5 |
| USP32P3 |
| GJB4 |
| SYCP2 |
| AC079610.2 |
| SPOCK3 |
| ZNF676 |
| C16orf93 |
| OR8A1 |
| SERPINA3 |
| S100A4 |
| FAT4 |
| ACADSB |
| CTSE |
| HRCT1 |
| OR2C3 |
| SFTA2 |
| BECN1P1 |
| ZNF729 |
| CPNE4 |
| NTNG2 |
| NUDT11 |
| ZNF781 |
| PRTN3 |
| S100A5 |
| NBPF4 |
| ESRRG |
| ARL9 |
| SPTSSB |
| FAM72A |
| LAMA2 |
| XRCC2 |
| MMP1 |
| ADH1B |
| LINC00173 |
| CLPSL2 |
| S100A2 |
| HCAR1 |
| FAM163B |
| ZNF300P1 |
| ZNF257 |
| MAGEA6 |
| GPR123 |
| LINC00336 |
| C6orf141 |
| GUCA2A |
| LYPD2 |
| UAP1L1 |
| ZNF98 |
| ZNF860 |
| DIO3 |
| DCHS2 |
| VEPH1 |
| GYPE |
| ZNF695 |
| COL4A6 |
| BCO2 |
| DMBX1 |
| KLKP1 |
| VN1R5 |
| NMB |
| CR1L |
| LHFPL5 |
| CYP2A13 |
| S100A6 |
| ELOVL2 |
| KEL |
| DLGAP2 |
| ZNF560 |
| TUBA3C |
| AVPR1B |
| DSCR8 |
| ADH4 |
| MB |
| BPIFA1 |
| CTC-490E21.13 |
| PEG3 |
| DCAF12L2 |
| TPTE2P3 |
| TMEM207 |
| TPM2 |
| ITGBL1 |
| WDHD1 |
| ZNF534 |
| FAM3D |
| TAT |
| PPP1R14C |
| APCDD1L |
| FAM169A |
| MUC2 |
| MAGEB3 |
| PAX9 |
| GRM3 |
| ARHGAP11A |
| PNMA5 |
| PRC1 |
| RASGEF1A |
| CSAG1 |
| SNORA73B |
| SNORA74A |
| RNU6-353P |
| RNA5SP334 |
| SNORA23 |
| VTRNA1-2 |
| RN7SK |
| RNU6-8 |
| VTRNA1-3 |
| RNU4-2 |
| RP11-498P14.5 |
| RP11-370F5.4 |
| E2F3P2 |
| RBMS3-AS2 |
| TAS2R2P |
| LINC00970 |
| LINC00501 |
| TCP10 |
| TEDDM1 |
| METTL11B |
| CENPW |
| FANK1 |
| HIST2H3C |
| PNLIPRP3 |
| RIPPLY2 |
| OOEP |
| LINC00632 |
| SAMD13 |
| CCDC160 |
| RP13-379L11.2 |
| RHOXF2B |
| CT83 |
| RP11-465L10.10 |
| RP11-126H7.4 |
| TCEAL5 |
| TDRG1 |
| NAP1L6 |
| CLPSL1 |
| GPRIN2 |
| COL11A2 |
| XAGE1A |
| XAGE1B |
| SLC44A4 |
| LY6G6C |
| POTEKP |
| AC079586.1 |
| C1orf195 |
| PRAMEF9 |
| MCCD1 |
| PSORS1C3 |
| POU5F1 |
| PSORS1C2 |
| PSORS1C1 |
| LINC01123 |
| OR2H2 |
| C9orf57 |
| IL31 |
| FAM153C |
| OR2H1 |
| LINC01556 |
| IGFL2 |
| KRT40 |
| KRT25 |
| FAM83A-AS1 |
| MS4A13 |
| PRSS1 |
| CLLU1OS |
| CCDC144NL |
| TRBV20OR9-2 |
| RP11-352D3.2 |
| KRT17P4 |
| SLCO6A1 |
| TP53TG3D |
| MUC19 |
| LINC00898 |
| VCX3B |
| RP11-15H7.2 |
| LINC00634 |
| SLCO1B7 |
| GAGE1 |
| LINC01531 |
| KLRC2 |
| C1QTNF9B |
| ONECUT3 |
| IFITM5 |
| SERPINB4 |
| KRTAP20-4 |
| EGFEM1P |
| GOLGA8O |
| CTD-2008L17.2 |
| DUXAP8 |
| FOXL2NB |
| TMEM200C |
| CD200R1L |
| XKR4 |
| RNVU1-7 |
| RNU6-1189P |
| RNU1-67P |
| RNU6-45P |
| SNORD15B |
| MIR621 |
| MIR320A |
| MT-TM |
| IGKC |
| IGKJ5 |
| IGKV3D-11 |
| IGLV1-50 |
| IGLV1-47 |
| IGLV1-44 |
| IGLV7-43 |
| IGLV3-27 |
| IGLV2-23 |
| IGLV3-21 |
| IGLV3-19 |
| IGLC2 |
| IGLC3 |
| IGHG4 |
| IGHG1 |
| IGHV3-21 |
| IGHV3-23 |
| IGHV1-24 |
| IGHV3-49 |
| IGHV1-69 |
| AP000807.1 |
| SNORD72 |
| SNORA74B |
| KRTAP2-3 |
| RP11-563J2.3 |
| AURKAPS1 |
| CFAP45 |
| AC104131.1 |
| TCTEX1D2 |
| EEF1DP5 |
| CCDC183 |
| AC018804.7 |
| PPP1R2P10 |
| RP1-29C18.9 |
| RP11-577H5.1 |
| HAUS7 |
| MAGEA12 |
| HNRNPA1P33 |
| GPC2 |
| FIRRE |
| RP11-428P16.2 |
| AC005077.8 |
| POLD2P1 |
| FAM210CP |
| HBE1 |
| ZNF99 |
| ZNF90 |
| RP11-414H17.5 |
| PGAM1P7 |
| ATP5F1P3 |
| RRM2P3 |
| MAGEB1 |
| AZGP1P2 |
| FOXI3 |
| LINC00488 |
| LCNL1 |
| KRT18P13 |
| LRRC37A4P |
| AS3MT |
| SEC14L6 |
| SPINK13 |
| AC087491.2 |
| LRRC37A11P |
| CPEB1 |
| SLC6A10P |
| CDIPT-AS1 |
| RP11-174G6.1 |
| RP11-37N22.1 |
| FER1L6 |
| KRT16P1 |
| AC004540.5 |
| CTD-2299I21.1 |
| SPATA31D1 |
| AKAP17BP |
| AC129492.6 |
| COL28A1 |
| PKMP5 |
| RP3-420J14.1 |
| RP11-313J2.1 |
| PRSS41 |
| MUC5AC |
| GOLGA6B |
| UBA52P6 |
| RP11-54D18.2 |
| RP11-563J2.2 |
| RP11-43F13.3 |
| LA16c-60H5.7 |
| ZNF663P |
| LINC00189 |
| FAM72D |
| LINC01139 |
| BTNL10 |
| RP11-364B6.1 |
| RPL37P15 |
| AC093850.1 |
| RBMXP1 |
| AC114776.1 |
| RP1-13D10.3 |
| AC091801.1 |
| AC099552.4 |
| KRT19P1 |
| RP11-157J24.1 |
| RP3-407E4.4 |
| RP3-407E4.3 |
| RAET1K |
| RP1-140K8.2 |
| AC008060.7 |
| RP1-34L19.1 |
| TDRD15 |
| RPS3AP2 |
| FAM19A5 |
| TPT1P8 |
| GCNT1P4 |
| RP11-40G16.1 |
| ACTBP8 |
| RP11-95M15.2 |
| RP11-69L16.5 |
| RPL21P28 |
| LL22NC03-63E9.3 |
| RP11-127B16.1 |
| SNORA77 |
| SNORD88A |
| SNORD99 |
| DPP3P2 |
| PLXNA4 |
| MAGEA3 |
| HMSD |
| TRIM16 |
| C7orf71 |
| AC007131.1 |
| RNA5SP118 |
| RNU2-63P |
| IGLV9-49 |
| CLDN10-AS1 |
| LINC00397 |
| RP11-285G1.9 |
| AC079613.1 |
| TMSB4XP4 |
| SMPD4P1 |
| AP001601.2 |
| CYP4F32P |
| CKMT1A |
| DSCR4-IT1 |
| AC104395.1 |
| RP5-1172N10.2 |
| RP11-314N2.2 |
| RP11-145E17.2 |
| RP11-644K8.1 |
| TSSC2 |
| MED15P9 |
| RP11-197K6.1 |
| AC004870.4 |
| RP1-56J10.8 |
| AC006372.5 |
| RP11-251G23.2 |
| LINC01014 |
| ROR1-AS1 |
| SMCR2 |
| AC111155.1 |
| POU6F2-AS1 |
| UBE2SP2 |
| AL109763.2 |
| RP4-798A10.4 |
| LINC00466 |
| RP11-191L9.4 |
| AC005077.7 |
| LINC01142 |
| RP3-326I13.1 |
| ANKRD30BP2 |
| CHL1-AS2 |
| RP11-462G2.2 |
| OACYLP |
| RP11-235G24.1 |
| LINC01272 |
| RP11-114B7.6 |
| SLC31A1P1 |
| GUSBP6 |
| C4BPAP1 |
| HTR2A-AS1 |
| RP11-561I11.3 |
| MEP1AP4 |
| LINC01087 |
| LPP-AS1 |
| AC096669.3 |
| RP4-612B18.1 |
| GAGE12J |
| MED15P4 |
| ZNF812 |
| RP11-179K3.2 |
| AC090945.1 |
| AC015987.1 |
| AP001065.7 |
| BRAFP1 |
| RP11-235D19.2 |
| TUBB4BP2 |
| RP11-190J1.3 |
| CDKN2A-AS1 |
| RP1-200K18.1 |
| RP11-3B12.5 |
| LINC00184 |
| PGM5-AS1 |
| GS1-309P15.2 |
| RP11-524H19.2 |
| RP11-204C23.1 |
| RP4-660H19.1 |
| OR52K3P |
| LINC00237 |
| AC012354.6 |
| XXbac-BPG308K3.5 |
| MIR137HG |
| DUXAP10 |
| HSPA7 |
| AC007250.4 |
| AC091814.2 |
| LA16c-83F12.6 |
| LINC00705 |
| LINC00113 |
| LHFPL3-AS2 |
| CT62 |
| TDGF1P3 |
| RP11-350E12.4 |
| PGM5P4 |
| RP11-3B7.1 |
| AC080125.1 |
| AC104134.2 |
| RAB6C-AS1 |
| RP11-736E3.1 |
| RP11-120J1.1 |
| RP11-377K22.2 |
| RP11-203F10.1 |
| SP3P |
| XX-C2158C6.3 |
| AC002480.5 |
| ZNF385D-AS1 |
| RP11-328J2.1 |
| AC098973.2 |
| LINC00505 |
| CADM3-AS1 |
| RP4-784A16.5 |
| AP000619.5 |
| FAM225B |
| PLAC4 |
| RP11-44M6.1 |
| LINC00626 |
| AC010970.2 |
| AC016582.2 |
| LINC01529 |
| LINC01456 |
| RPS27P16 |
| NTF4 |
| ELFN1 |
| ABHD11-AS1 |
| MTND1P23 |
| LAMP5-AS1 |
| RP11-10J5.1 |
| LGALS17A |
| PHF2P2 |
| AF277315.13 |
| KRT18P36 |
| RP1-18D14.7 |
| GRM7-AS3 |
| RP11-274B18.4 |
| DCAF8L1 |
| MRPL23-AS1 |
| RP13-401N8.1 |
| AC018641.7 |
| RP11-551L14.4 |
| RP11-776H12.1 |
| LINC00323 |
| AC126365.1 |
| AC012512.1 |
| CYP4F26P |
| AC017060.1 |
| KIF4B |
| TEX41 |
| AC007966.1 |
| PP12613 |
| KIAA0125 |
| RP11-148B18.3 |
| ERVMER34-1 |
| ISCA2P1 |
| BSN-AS2 |
| ALG1L5P |
| RP4-714D9.4 |
| CTD-2328D6.1 |
| RP1-97J1.2 |
| AC007285.6 |
| AC007036.6 |
| SLC8A1-AS1 |
| LINC00511 |
| RP11-395B7.4 |
| AP000472.3 |
| RP11-4C20.4 |
| AC084149.2 |
| RP11-267C16.1 |
| LBX1-AS1 |
| RP11-552E20.1 |
| RP11-545A16.3 |
| IL21-AS1 |
| MIR663AHG |
| SPANXB2 |
| AC009502.4 |
| AC005042.2 |
| LINC00866 |
| RP11-112J3.16 |
| AC012501.2 |
| RPL37P1 |
| RP11-179H18.5 |
| ATP5G1P4 |
| AC124861.1 |
| LINC01483 |
| RP11-202G18.1 |
| SLC2A1-AS1 |
| RP11-492E3.2 |
| SOX21-AS1 |
| RP11-307P5.1 |
| RP11-445N18.5 |
| CROCCP4 |
| RP11-15M15.2 |
| RPSAP64 |
| RP4-565E6.1 |
| RP4-738P11.3 |
| AC015922.5 |
| SLC9A7P1 |
| RP4-533D7.5 |
| LINC00948 |
| FLJ31104 |
| RP13-16H11.2 |
| ZNF680P1 |
| HNRNPA1P46 |
| RP4-663N10.1 |
| RP4-781K5.4 |
| RP11-552D4.1 |
| MFI2-AS1 |
| RP11-165F24.5 |
| RP11-342M1.3 |
| RNF144A-AS1 |
| C1orf143 |
| RP11-541F9.2 |
| GAPDHP1 |
| AP000593.5 |
| RP11-184A2.2 |
| UGT1A2P |
| TMSB10P1 |
| RP11-339B21.8 |
| AC093159.1 |
| RP11-114M1.1 |
| RP11-170M17.1 |
| ZNF883 |
| RP11-1B20.1 |
| RP11-495P10.9 |
| RP5-1051H14.2 |
| AC079779.4 |
| RP5-968D22.1 |
| RP11-155G14.1 |
| AL109763.1 |
| RP11-284G10.1 |
| SRGAP3-AS2 |
| RP5-884M6.1 |
| AC004022.8 |
| CBX3P6 |
| AC090954.5 |
| OR7E128P |
| RP11-567G11.1 |
| SATB1-AS1 |
| UBTFL6 |
| AC006483.5 |
| LINC01205 |
| IGHV3-47 |
| AC016710.1 |
| AC069513.4 |
| LINC00200 |
| AC098973.1 |
| LINC00377 |
| RP11-227H15.4 |
| RP4-580N22.2 |
| RP11-431K24.2 |
| DLEU1-AS1 |
| PIN1P1 |
| SZT2-AS1 |
| LINC00452 |
| LINC00858 |
| AC007349.4 |
| CTD-2554C21.3 |
| SACS-AS1 |
| LINC01204 |
| RP11-423C15.3 |
| LINC01501 |
| AC011288.2 |
| RP11-330A16.1 |
| AC016753.7 |
| RP11-435P24.2 |
| RP11-536K7.5 |
| RP11-184A2.3 |
| ZNF492 |
| XX-CR54.1 |
| RP4-738P11.4 |
| AC013444.1 |
| RP11-398K22.12 |
| CTC-546K23.1 |
| RP11-363N22.2 |
| CASC20 |
| RP11-399E6.4 |
| RPS4XP5 |
| KIF25-AS1 |
| PRPS1L1 |
| TFAP2A-AS1 |
| FLJ31356 |
| RP11-366F6.2 |
| AC007128.1 |
| RP11-15I11.2 |
| MIR181A1HG |
| RP11-70J12.1 |
| ANKRD36BP2 |
| RP4-568F9.6 |
| RP11-481H12.1 |
| RP11-76N22.1 |
| RP11-172E9.2 |
| TRPM2-AS |
| PRRT3-AS1 |
| RP11-385N23.1 |
| RP11-282I1.1 |
| DDX43P3 |
| AC002539.1 |
| HERC2P4 |
| RP1-40E16.9 |
| ELOVL2-AS1 |
| FEZF1-AS1 |
| AC009234.1 |
| AC004538.3 |
| NCAPD2P1 |
| RP4-737E23.2 |
| RP5-1139I1.1 |
| LINC00856 |
| ERVMER61-1 |
| AC114803.3 |
| AC004160.4 |
| NEK2P4 |
| ANKRD18B |
| RP3-437I16.1 |
| NOS2P3 |
| HMGB1P11 |
| LINC01183 |
| RP5-828H9.1 |
| RP11-69L16.6 |
| GPAA1P2 |
| RP11-486B10.4 |
| AC016682.1 |
| AC112229.1 |
| FOXD3-AS1 |
| LINC01362 |
| GOT2P2 |
| RP4-595K12.2 |
| MAGEA8-AS1 |
| RP11-775L16.1 |
| HHATL-AS1 |
| RP11-113I24.1 |
| AC092625.1 |
| AC013275.2 |
| AC008271.1 |
| RP11-122M14.1 |
| NPM1P9 |
| LINC01436 |
| LINC01508 |
| LINC01468 |
| AC093702.1 |
| LINC01098 |
| AC007099.1 |
| AC005592.2 |
| FARP1-AS1 |
| AC011752.1 |
| ZNF826P |
| ZBTB46-AS1 |
| RP11-436K8.1 |
| APCDD1L-AS1 |
| LINC00704 |
| AC103563.2 |
| LINC00689 |
| AC006050.2 |
| RP5-899B16.1 |
| FAR2P4 |
| WASIR2 |
| RP11-472M19.2 |
| LARP1BP1 |
| AC024937.6 |
| LINC00032 |
| AC024937.4 |
| RP5-1170K4.7 |
| RP11-336A10.5 |
| AC108868.5 |
| LINC01249 |
| RP1-232L24.3 |
| PTCHD3P2 |
| LINC01037 |
| DLG3-AS1 |
| LINC01090 |
| TSPAN19 |
| ABCA9-AS1 |
| RP1-154K9.2 |
| RP1-90L14.1 |
| PIK3CD-AS2 |
| LINC01198 |
| C18orf42 |
| RP5-1120P11.3 |
| RP11-281A20.1 |
| AC007682.1 |
| NEBL-AS1 |
| AC102953.6 |
| RP11-573D15.1 |
| CLCN3P1 |
| AC108868.6 |
| AC005537.2 |
| AC009410.1 |
| LINC01105 |
| AL035610.1 |
| AL022344.2 |
| RP11-149I23.3 |
| NCOA7-AS1 |
| RP4-659I19.1 |
| RP11-398M15.1 |
| RP11-573D15.2 |
| RP11-4C20.3 |
| RP1-124C6.1 |
| RP11-655G22.1 |
| RP11-263F14.3 |
| RP1-102D24.5 |
| BPESC1 |
| RP11-416N4.1 |
| RP11-5P18.10 |
| TGFB2-AS1 |
| RP5-1011O1.2 |
| AC112198.1 |
| RP11-332K15.1 |
| RP11-14N7.2 |
| AC007386.4 |
| LINC01517 |
| AC002511.3 |
| YWHAEP1 |
| AC073043.1 |
| RP11-15B24.4 |
| RP5-1185I7.1 |
| RP11-382F24.1 |
| AP000525.10 |
| SLCO4A1-AS1 |
| RP4-665N4.4 |
| RP11-746P2.3 |
| RP4-597N16.1 |
| TTLL7-IT1 |
| AC005009.2 |
| RP11-440G5.2 |
| XXyac-YX65C7_A.3 |
| CCDC144NL-AS1 |
| HOXB-AS3 |
| RP11-301L8.2 |
| ACTBP12 |
| HMGA1P8 |
| CTD-2020K17.3 |
| RNF224 |
| RP11-67L3.4 |
| AC002511.2 |
| RP11-220I1.2 |
| RP11-415J8.5 |
| RP11-760D2.5 |
| AC092159.2 |
| RPS6P12 |
| DSCR10 |
| RP11-482E14.1 |
| RP1-209A6.1 |
| RP11-202K23.1 |
| MTND4P20 |
| RP11-318G21.4 |
| RP4-569D19.5 |
| AP000688.29 |
| RP4-736H5.3 |
| RP11-175I6.5 |
| RP11-63N8.3 |
| STXBP5-AS1 |
| AC011322.1 |
| AC010091.1 |
| LINC01518 |
| LINC00460 |
| KRT8P15 |
| RP4-694A7.2 |
| LINC00462 |
| RP5-905G11.3 |
| RP11-342M1.7 |
| GOT2P3 |
| AP000695.4 |
| AC005083.1 |
| POU6F2-AS2 |
| DLG5-AS1 |
| RP5-1061H20.5 |
| AL133493.2 |
| RP11-305F18.1 |
| CTXN2 |
| UBE2SP1 |
| RP11-297H3.3 |
| AC013439.4 |
| GAPDHP22 |
| RP11-452D2.2 |
| RP11-308D16.2 |
| PAGE2 |
| MTND4P11 |
| RP11-276E17.2 |
| AKAP8P1 |
| RP3-460G2.2 |
| AC004854.4 |
| AC006372.4 |
| AC003988.1 |
| RP11-353N4.5 |
| DEPDC1-AS1 |
| RP11-432J24.3 |
| AC009264.1 |
| SNX18P7 |
| ELK2BP |
| RP11-459O1.2 |
| MAGI2-AS3 |
| GS1-421I3.2 |
| C5orf58 |
| RP11-100G15.7 |
| MCIDAS |
| SNRK-AS1 |
| WDR82P1 |
| AC009263.2 |
| IFT74-AS1 |
| RP11-445L6.3 |
| AC002076.10 |
| AC060834.3 |
| RP11-166B2.1 |
| LINC01287 |
| RP11-336N8.4 |
| PHBP13 |
| LINC00676 |
| RP3-439F8.1 |
| RP11-20F24.2 |
| RP11-356I2.1 |
| FABP5P7 |
| RP1-150O5.3 |
| AC068138.1 |
| RP3-417L20.4 |
| RP11-374P20.4 |
| C12orf75 |
| RP11-121A14.2 |
| TATDN2P3 |
| AF127577.8 |
| AC137723.5 |
| AC090627.1 |
| RP11-165F24.2 |
| AC016723.4 |
| RP11-561N12.6 |
| RP11-428G2.1 |
| GS1-600G8.5 |
| AC006373.1 |
| DPY19L2P4 |
| AC092415.1 |
| RP11-498P14.4 |
| AC012506.4 |
| AC087499.5 |
| AJ239322.1 |
| RP1-69M21.2 |
| AC064850.4 |
| RPL21P40 |
| LINC00993 |
| AP000351.13 |
| AC013268.3 |
| RP11-32D17.4 |
| AC023347.1 |
| IFITM4P |
| CNN2P3 |
| LINC00941 |
| AC018816.3 |
| GRAMD4P2 |
| RP3-470B24.5 |
| LINC01036 |
| AC019117.2 |
| LL22NC03-104C7.1 |
| GAPDHP14 |
| AC010127.3 |
| AC064853.2 |
| RP11-343J3.2 |
| LINC01361 |
| CLEC2L |
| AP001189.4 |
| AC005027.3 |
| TRHDE-AS1 |
| PSMC1P10 |
| SUCLG2P2 |
| RP11-440G9.1 |
| RP11-320G24.1 |
| VLDLR-AS1 |
| CT47B1 |
| AC018890.6 |
| PKMP1 |
| SIX3-AS1 |
| KLF2P4 |
| ARF4P2 |
| HNRNPA1P54 |
| MED28P7 |
| RP11-95P9.1 |
| AC023115.4 |
| RP11-473A10.2 |
| DLX2-AS1 |
| ARHGEF38 |
| AC078941.1 |
| BCYRN1 |
| RP11-401L13.5 |
| LINC01474 |
| RP11-408N14.1 |
| RP3-329E20.2 |
| NF1P8 |
| RP11-451O13.1 |
| GAPDHP21 |
| AC010987.6 |
| AC015922.6 |
| AC005550.3 |
| RP5-836J3.1 |
| AC105399.2 |
| AC004022.7 |
| IMPDH1P4 |
| RP11-13E5.2 |
| RP11-402P6.9 |
| CKMT1B |
| UPP2-IT1 |
| RNF223 |
| LINC01502 |
| RP11-145M4.3 |
| CAMTA1-IT1 |
| LINC01522 |
| FOXD2-AS1 |
| RP11-312B8.1 |
| CECR7 |
| BHMG1 |
| RP11-67L3.2 |
| RP11-280O1.2 |
| AC008268.2 |
| UNC5B-AS1 |
| SHISA9 |
| LINC00383 |
| RP11-462G2.1 |
| ERICH1-AS1 |
| KIFC1 |
| RP11-655G22.2 |
| LINC01115 |
| RP11-360O19.4 |
| RP5-1120P11.1 |
| IRGM |
| AC010980.2 |
| CTD-2571E19.3 |
| AC073130.1 |
| AC092839.1 |
| RP11-90H3.2 |
| XXbac-BPG27H4.8 |
| AC008746.10 |
| KCNMB2-AS1 |
| RP11-159M11.2 |
| DDX39BP2 |
| AC002480.2 |
| SPATA3-AS1 |
| RP11-435F13.2 |
| FMO8P |
| SLC25A24P2 |
| CTA-941F9.9 |
| AC068196.1 |
| MKRN4P |
| PAGE2B |
| BX470102.3 |
| TBC1D3P1 |
| LINC01448 |
| MIR1270 |
| RPS20P22 |
| CLRN1-AS1 |
| RP11-615J4.3 |
| PCDHA13 |
| RP11-260O18.1 |
| RN7SL221P |
| RN7SL583P |
| RP11-451B8.1 |
| LINC00879 |
| RP11-88I21.1 |
| MEIKIN |
| PRAMEF11 |
| RN7SL368P |
| IGKV3-20 |
| RP11-451G4.2 |
| SMKR1 |
| CPHL1P |
| RP11-314M24.1 |
| TCAM1P |
| ACAD11 |
| RP11-331F4.4 |
| RP1-228P16.1 |
| LINC01212 |
| RP11-185E8.1 |
| CDKN2B-AS1 |
| TM4SF1-AS1 |
| AC006539.2 |
| RP11-155G14.6 |
| ATP8A2P1 |
| LSAMP-AS1 |
| PLGLA |
| RP11-313M3.1 |
| HOXA11-AS |
| RPL29P14 |
| ARHGAP31-AS1 |
| RP11-3K24.1 |
| RP11-10O22.1 |
| TDGF1 |
| RP11-221J22.2 |
| RPL34P33 |
| CDRT1 |
| IGKV3-11 |
| RP11-758I14.3 |
| SLC25A24P1 |
| LINC01192 |
| CD302 |
| FAM195CP |
| RPL37P6 |
| RP4-781K5.5 |
| SSX2 |
| RP11-641D5.2 |
| RP11-381E24.1 |
| UGT1A1 |
| RPS23P6 |
| INMT |
| VWFP1 |
| SUMO1P1 |
| WWTR1-IT1 |
| GS1-388B5.1 |
| IGKV1-33 |
| RP11-738B7.1 |
| RP11-340E6.1 |
| SNHG3 |
| RP13-463N16.6 |
| HOXB-AS4 |
| RP11-368I23.2 |
| UGT1A8 |
| RP11-501O2.5 |
| UGT1A10 |
| POU5F1P6 |
| AC012501.3 |
| MED28P8 |
| CSAG4 |
| RP11-302F12.1 |
| RPS16P9 |
| IGKV1D-17 |
| RP11-47P18.2 |
| RP11-451G4.3 |
| RP11-651P23.5 |
| RP11-47P18.1 |
| RN7SL180P |
| RP11-118N24.2 |
| RN7SL233P |
| GK-AS1 |
| UGT1A3 |
| RP11-115N4.1 |
| VSIG8 |
| RN7SL8P |
| AF165138.7 |
| IGKV1-5 |
| AOX2P |
| RP11-364B6.2 |
| CTC-458A3.1 |
| RP4-669B10.3 |
| LEFTY1 |
| HOTTIP |
| KIR2DL3 |
| RPL7P18 |
| RP5-1096J16.1 |
| HMGB1P38 |
| TUBA4B |
| RP11-167H9.4 |
| RN7SL684P |
| RP5-839B4.8 |
| RP1-300I2.2 |
| IL12A-AS1 |
| RPS18P6 |
| AC024704.2 |
| IGKV2-28 |
| UGT1A7 |
| LINC01322 |
| EIF4E2P2 |
| RP11-810P12.1 |
| LINC01296 |
| RP11-119K6.6 |
| RPL30P7 |
| KRTAP5-7 |
| IGKV3-15 |
| UGT1A4 |
| RPL18P13 |
| LINC01391 |
| CCDC13 |
| CTD-2377D24.6 |
| AL109761.5 |
| MTHFD2P1 |
| FCGR2C |
| RP11-320N21.1 |
| CTD-2540L5.6 |
| RP11-540A21.2 |
| LINC00461 |
| DDX11-AS1 |
| RP11-277P12.20 |
| RP11-184E9.1 |
| CRNDE |
| NADK2-AS1 |
| RP11-410D17.2 |
| RP11-179A16.1 |
| GS1-24F4.2 |
| RP11-158I9.5 |
| SNHG6 |
| LINC01096 |
| CASC8 |
| PRR7-AS1 |
| RP5-1186N24.3 |
| RP11-461O7.1 |
| RP11-57A19.2 |
| RP11-159H10.3 |
| RP11-1094H24.4 |
| RP11-325N19.3 |
| RP11-519M16.1 |
| MIR210HG |
| RP11-296I10.3 |
| RP11-266N13.2 |
| RP11-629G13.1 |
| RP11-422N16.3 |
| INSL3 |
| CTC-338M12.9 |
| LINC01019 |
| LINC01194 |
| RP11-446J8.1 |
| PES1P1 |
| RP11-722M1.1 |
| RP11-83C7.1 |
| CTC-499J9.1 |
| CTD-2218G20.2 |
| LUCAT1 |
| CTC-327F10.5 |
| RP11-366H4.1 |
| AC005150.1 |
| RP11-826N14.2 |
| RP11-479O16.1 |
| CTD-3007L5.1 |
| ASNSP1 |
| RP11-145G20.1 |
| CTC-458G6.2 |
| BCL2L12P1 |
| OTX2-AS1 |
| GDNF-AS1 |
| FLJ42969 |
| RP11-206P5.2 |
| RP11-1267H10.4 |
| LINC00992 |
| HAS2-AS1 |
| LINC01085 |
| RP11-766F14.2 |
| RP11-31K23.2 |
| RP11-632F7.1 |
| RP11-308B16.2 |
| RP11-173E2.1 |
| RP11-664D7.4 |
| RP11-362F19.1 |
| PMPCAP1 |
| RP11-252I13.1 |
| RP11-412P11.1 |
| HHIP-AS1 |
| RP11-94H18.1 |
| CTD-2256P15.1 |
| RP11-445O3.2 |
| CTD-2251F13.1 |
| EFTUD1P2 |
| RP11-742B18.1 |
| RP5-951N9.1 |
| LINC01033 |
| CTD-2325A15.3 |
| RP11-381N20.2 |
| RP11-1C1.6 |
| RP11-124N3.3 |
| RP11-669N7.2 |
| AP000304.12 |
| LINC01088 |
| CTB-78O21.1 |
| CASC11 |
| LINC01060 |
| CASC9 |
| RP11-1C1.4 |
| RP11-1E22.1 |
| NACAP5 |
| ZEB2P1 |
| LINC01258 |
| RP11-366M4.17 |
| RP11-496H1.1 |
| LINC00942 |
| RP11-281P23.2 |
| CTB-138E5.1 |
| RP11-552M14.1 |
| RP11-44F21.3 |
| RP11-83M16.4 |
| RP11-618I10.1 |
| RP11-114H21.2 |
| RP11-269F21.3 |
| HS3ST5 |
| AC142293.3 |
| CCT7P2 |
| HMGB1P3 |
| RP11-332J15.2 |
| RP11-141O11.1 |
| YJEFN3 |
| CTD-2116N20.1 |
| RP11-314N14.1 |
| CTD-2013M15.1 |
| RP11-380P13.1 |
| CTC-338M12.5 |
| CTB-49A3.4 |
| CTB-1I21.1 |
| CTD-2269F5.1 |
| RP11-310P5.2 |
| RP11-448G15.1 |
| AACSP1 |
| KIAA1210 |
| RP11-834C11.5 |
| CTD-2081C10.1 |
| FAM218A |
| RP11-8L2.1 |
| RP11-47I22.2 |
| KNOP1P5 |
| LINC01511 |
| RP11-565A3.2 |
| RP11-138B4.1 |
| LINC00491 |
| RP11-161D15.1 |
| RP11-567N4.3 |
| RP11-241F15.10 |
| RP11-153M7.3 |
| RP11-12K22.1 |
| RP13-884E18.4 |
| RP11-501C14.5 |
| CTC-480C2.1 |
| ZFPM2-AS1 |
| RP11-685F15.1 |
| RP11-168A11.4 |
| RP11-115D19.1 |
| RP11-280G9.1 |
| RP11-734I18.1 |
| RP11-81H3.2 |
| CTD-2532K18.2 |
| RP11-281P23.1 |
| RP11-540O11.1 |
| CTD-2325A15.2 |
| RP11-401E5.2 |
| RP11-542G1.1 |
| LINC00589 |
| RP11-400D2.2 |
| RP11-469N6.1 |
| CTD-2263F21.1 |
| RFPL4B |
| RP11-549K20.1 |
| ERVH-1 |
| RP11-81H14.2 |
| CTD-2003D5.1 |
| CTC-327F10.4 |
| RP11-328N19.1 |
| CTD-2091N23.1 |
| RP11-129M6.1 |
| RP11-332J15.3 |
| LINC00958 |
| RP11-397E7.4 |
| RP11-369K16.1 |
| FOXD1 |
| RP11-197N18.7 |
| LINC01099 |
| RP11-109E24.1 |
| RP11-468N14.5 |
| RP11-501C14.7 |
| AC008427.2 |
| RP11-610P16.1 |
| RP11-774D14.1 |
| RP11-192H6.2 |
| RP11-614F17.2 |
| RNU7-123P |
| RNU6-1161P |
| RNA5SP154 |
| RNU7-154P |
| RNY3P16 |
| RNU6-1337P |
| RP11-369E15.4 |
| RP11-100L22.1 |
| RP11-383J24.1 |
| IGLV2-5 |
| CTB-37A13.1 |
| RP1-170O19.17 |
| RP11-675F6.3 |
| RP11-962G15.1 |
| TRNP1 |
| RP11-44D19.1 |
| RP11-1102P16.1 |
| LINC00534 |
| EVX1-AS |
| SRPK2P |
| IGHV7-40 |
| RP11-109P6.2 |
| IGKV1-13 |
| RP11-393K19.1 |
| RP1-170O19.14 |
| RP11-586K2.1 |
| RP11-32K4.1 |
| VENTXP5 |
| RP11-284H18.1 |
| RP11-573J24.1 |
| RP11-770E5.1 |
| KB-1980E6.3 |
| RP11-317N12.1 |
| RP11-1114I9.1 |
| ZFHX4-AS1 |
| RP11-359E19.2 |
| KB-1615E4.2 |
| RP11-758M4.4 |
| RP11-697M17.2 |
| RP11-527N22.2 |
| IGHGP |
| RP11-439C15.4 |
| RP11-1084E5.1 |
| LINC01170 |
| KB-173C10.2 |
| RP11-946L20.2 |
| LINC01419 |
| KB-1980E6.2 |
| RP11-44K6.3 |
| IGHV3-22 |
| CTB-43E15.1 |
| NRG1-IT1 |
| IGKV2-29 |
| RP11-363E6.3 |
| RP11-574O7.1 |
| RP3-388N13.3 |
| RP11-369E15.3 |
| RP11-675F6.4 |
| RP11-30J20.1 |
| RP11-705O24.1 |
| PCDHGB7 |
| CD8BP |
| RP11-219B4.3 |
| KB-1043D8.6 |
| PCDHGA3 |
| RP11-320N21.2 |
| RP11-662G23.1 |
| RP11-369E15.1 |
| RP11-594N15.2 |
| GLYATL1P4 |
| RP11-347E10.1 |
| CTD-2562J17.7 |
| RP11-33I11.2 |
| RP11-405K6.1 |
| OR2AT1P |
| RP11-84A19.3 |
| RP11-429J17.5 |
| BBOX1-AS1 |
| MAGEL2 |
| OR7E126P |
| NAV2-AS4 |
| CTD-2589M5.5 |
| AC068858.1 |
| RTL1 |
| RP11-7I15.4 |
| FAM8A2P |
| CTD-2140G10.2 |
| RP11-672A2.4 |
| RP11-680E19.1 |
| RP11-574M7.1 |
| RP11-728F11.3 |
| RP11-676F20.1 |
| RP11-350N15.3 |
| RP11-94P11.4 |
| RP11-326C3.2 |
| RP11-347H15.5 |
| SAA2-SAA4 |
| GRM5-AS1 |
| MIR100HG |
| RP11-406D1.2 |
| RP11-21L23.3 |
| RP11-839D17.3 |
| RP11-626H12.1 |
| RP11-350N15.4 |
| RP4-541C22.5 |
| CTD-3065J16.9 |
| CTD-2210P24.3 |
| AC025016.1 |
| WTAPP1 |
| OR8G5 |
| RP5-901A4.1 |
| RP11-428C19.4 |
| RP11-655M14.13 |
| RP11-708L7.6 |
| CTD-2337I7.1 |
| CTD-3064C13.1 |
| UBTFL10 |
| CTD-2562J17.2 |
| OR8G3P |
| CARD18 |
| RP11-415I12.2 |
| RP4-809F18.1 |
| RP11-564D11.3 |
| RP11-283I3.1 |
| RP11-766N7.3 |
| RP11-283G6.5 |
| LINC01479 |
| RP11-157G21.2 |
| AP000593.7 |
| RP11-167N4.2 |
| RP11-662I13.2 |
| RP11-43N5.1 |
| RP11-1038A11.1 |
| ARAP1-AS1 |
| RP11-728G15.1 |
| RP5-1154L15.2 |
| RP11-959F10.4 |
| RP11-982M15.6 |
| DYX1C1 |
| A2MP1 |
| RP11-319E16.1 |
| LINC00944 |
| RP11-76C10.5 |
| RP11-881M11.4 |
| ZNF486 |
| RP11-771K4.1 |
| NTAN1P3 |
| RP11-1038A11.3 |
| RP11-118B22.4 |
| RP11-705C15.4 |
| SALL3 |
| AC156455.1 |
| OR13A1 |
| RP11-424C20.2 |
| RP11-7M8.2 |
| RP11-405A12.1 |
| RP11-283G6.3 |
| RP11-707G14.8 |
| RP11-474D1.2 |
| GPR123-AS1 |
| KHDC1L |
| RP11-993B23.3 |
| RP11-545J16.1 |
| U47924.27 |
| RP11-25I15.3 |
| CLLU1 |
| RP11-626P14.2 |
| RP11-328C8.4 |
| DYNLL1P4 |
| C12orf79 |
| PRELID2P1 |
| RP11-511B23.1 |
| RP11-161H23.8 |
| RP11-1016B18.1 |
| RP11-469H8.6 |
| RP3-473L9.4 |
| RP11-968A15.2 |
| OVCH1-AS1 |
| RP11-185N2.1 |
| LINC01481 |
| RP11-1103G16.1 |
| RP11-474B16.1 |
| RP11-579D7.4 |
| RP11-620J15.3 |
| RP11-818F20.5 |
| RP11-1220K2.2 |
| RP11-295G12.1 |
| NOVA1-AS1 |
| CASC18 |
| RP11-46I1.2 |
| CTD-2591A6.2 |
| RP11-587P21.2 |
| RP11-493L12.5 |
| RP11-248E9.6 |
| RP11-161H23.10 |
| RP11-210M15.1 |
| RP11-386G11.10 |
| RP11-148E17.1 |
| CTD-2384A14.1 |
| CTD-2021H9.3 |
| RP11-30H9.1 |
| RP11-384J4.2 |
| RP11-493L12.6 |
| RP11-61A14.1 |
| RP11-314D7.2 |
| MKRN9P |
| RP11-315E17.1 |
| RP11-588H23.3 |
| RP11-1105G2.4 |
| RP11-248E9.7 |
| CLEC5A |
| RP1-46F2.3 |
| LINC00592 |
| RP11-314D7.1 |
| RP11-690J15.1 |
| CTD-2314B22.1 |
| RP11-274M17.1 |
| RP11-161H23.9 |
| RP11-536G4.2 |
| RP11-711D18.2 |
| RP11-895M11.2 |
| RP11-574F21.3 |
| RP11-187E13.1 |
| LINC00640 |
| DIO3OS |
| ASB9P1 |
| RP4-755D9.1 |
| RP11-108M12.3 |
| RP11-492D6.3 |
| RP11-857B24.1 |
| CTD-2298J14.2 |
| LINC01397 |
| LINC01269 |
| RP11-218E20.3 |
| RP11-406A9.2 |
| LINC01579 |
| RP11-110A12.2 |
| HIF1A-AS1 |
| LINC01580 |
| RP11-408B11.2 |
| RP11-76E17.4 |
| CTD-2566J3.1 |
| CTD-2014B16.3 |
| DUXA |
| EGLN3-AS1 |
| RP11-644F5.15 |
| RP11-1029J19.4 |
| RP11-1070N10.5 |
| CTD-3006G17.2 |
| LINC00519 |
| TMEM179 |
| RP11-398E10.1 |
| RPPH1 |
| RP11-561B11.6 |
| PTBP1P |
| TRAV30 |
| RP11-603B24.2 |
| CTD-3098H1.2 |
| RP11-187O7.3 |
| RP11-99L13.2 |
| LINC00648 |
| RP11-219E7.3 |
| LINC00924 |
| RP6-65G23.3 |
| CHEK2P2 |
| RP11-1029J19.2 |
| RP5-991G20.2 |
| RP11-1008C21.1 |
| RP11-684B21.1 |
| RP11-643M14.1 |
| RP11-60L3.1 |
| RP11-519G16.5 |
| RP11-519G16.3 |
| RP11-736N17.4 |
| RP11-346D14.1 |
| RP11-758N13.3 |
| RP11-265N7.2 |
| RP11-566K19.5 |
| CERS3-AS1 |
| RP11-276M12.1 |
| CPEB1-AS1 |
| RP11-227D13.4 |
| RP11-753A21.1 |
| CTD-2147F2.1 |
| RP11-210M15.2 |
| RP11-352D13.5 |
| RP11-685G9.2 |
| LINC01583 |
| RP11-499F3.1 |
| RP11-557C18.4 |
| RP11-253M7.6 |
| CTD-2147F2.2 |
| RP11-356M20.1 |
| RP11-499F3.2 |
| CTD-2184D3.5 |
| RP11-138H8.6 |
| FKSG62 |
| RP11-505E24.2 |
| RP11-20G13.2 |
| RP11-304L19.12 |
| CTD-2118P12.1 |
| RP11-23E19.2 |
| SLC22A31 |
| AC002519.8 |
| LYPD8 |
| RP11-63E9.1 |
| RP11-467L24.1 |
| AC097374.2 |
| CTA-363E6.1 |
| RP11-830F9.5 |
| RP11-293B20.2 |
| RP11-244B22.3 |
| RP6-91H8.2 |
| RP11-2E11.5 |
| CTA-363E6.2 |
| CTD-2336H13.2 |
| RARRES2P8 |
| RP11-1035H13.2 |
| RP11-218F4.1 |
| HOXB7 |
| RP11-488I20.9 |
| RP11-715J22.3 |
| AOC4P |
| RP11-326A19.4 |
| LA16c-312E8.2 |
| RP11-80F22.7 |
| RP11-146F11.5 |
| RP11-481J2.2 |
| RP11-229P13.25 |
| RP11-83N9.5 |
| RP1-239B22.5 |
| FRRS1L |
| RP11-274H24.1 |
| AP001063.1 |
| RP11-44F21.5 |
| CTD-2535I10.1 |
| RP11-973H7.1 |
| RP13-735L24.1 |
| RP5-912I13.1 |
| ATP2A1-AS1 |
| RP11-219B4.7 |
| RP11-55K13.1 |
| RP13-192B19.2 |
| RP11-432I5.2 |
| CTC-457E21.1 |
| HERC2P5 |
| LA16c-385E7.1 |
| RP11-368L12.1 |
| RP11-69H7.2 |
| RP11-326L17.1 |
| AC026471.6 |
| RP11-120K18.2 |
| RP11-184E9.2 |
| RP11-445O3.3 |
| CTD-2008P7.1 |
| RP11-80F22.15 |
| RP11-394I13.1 |
| LINC01229 |
| RP11-211G23.2 |
| LINC00565 |
| RP11-214L13.1 |
| RP11-483P21.6 |
| RP11-488I20.8 |
| RP11-150D5.2 |
| RP11-534L20.5 |
| LINC01572 |
| RARRES2P6 |
| RP11-417E7.2 |
| WFDC21P |
| RP11-14K3.4 |
| RP11-545A16.4 |
| RP11-7K24.3 |
| RP3-523K23.2 |
| RP11-496D24.2 |
| RP4-798A10.7 |
| CTD-2015G9.2 |
| RNF126P1 |
| AC004449.6 |
| RP11-443K8.1 |
| AC140912.1 |
| ANKRD26P1 |
| RP11-389G6.3 |
| RP11-279O17.1 |
| CTD-2568A17.1 |
| AC006538.1 |
| PDCD6IPP2 |
| RP4-616B8.4 |
| CTD-2083E4.7 |
| LINC00559 |
| RP11-757F18.5 |
| RP11-96H17.1 |
| RP11-527L4.2 |
| RP11-524C21.2 |
| AC002310.17 |
| HERC2P8 |
| RP11-575H3.1 |
| RP11-414J4.2 |
| CSDAP1 |
| RP11-352D13.6 |
| RP11-309H21.3 |
| GOLGA6L7P |
| GS1-204I12.4 |
| RP4-668J24.2 |
| LINC00922 |
| RP11-103J17.2 |
| RP11-650L12.2 |
| WI2-89031B12.1 |
| RP4-555D20.2 |
| TCF24 |
| RP1-118J21.25 |
| RP11-244B22.11 |
| MIA |
| RP11-63A1.1 |
| GFY |
| RP11-676J12.7 |
| SNORD3B-2 |
| CTD-2033A16.3 |
| RP11-353N14.4 |
| RP11-160E2.6 |
| U95743.1 |
| MMP12 |
| CCER2 |
| SPON1 |
| RP11-485G7.6 |
| RP11-353N14.1 |
| C19orf84 |
| TMPOP2 |
| RP11-485G7.5 |
| RP11-1055B8.2 |
| LA16c-325D7.1 |
| CTC-508F8.1 |
| RP5-890E16.2 |
| RP11-973F15.2 |
| RP11-963H4.3 |
| FAM72C |
| RP11-78F17.1 |
| HID1-AS1 |
| AF186192.6 |
| MSMB |
| RP11-57A1.1 |
| RP11-169F17.1 |
| RP11-161I6.2 |
| GDF2 |
| AC139530.1 |
| CTD-3010D24.3 |
| SNORD3A |
| RP11-739N10.1 |
| RNU4ATAC |
| ANXA8L1 |
| MIR3125 |
| RN7SL208P |
| MYH4 |
| RP3-388N13.5 |
| AC003006.1 |
| RP11-556O9.2 |
| RP13-516M14.2 |
| KRT18P8 |
| RP11-63N3.1 |
| RP11-260A9.6 |
| RP11-311F12.2 |
| MIR1273F |
| RP11-465I4.3 |
| RP11-1109M24.5 |
| SNORD3B-1 |
| ANXA8 |
| RP11-775G23.1 |
| IGLJCOR18 |
| RP11-723G8.2 |
| CTD-2510F5.4 |
| FCGR1C |
| MAFG-AS1 |
| RP13-516M14.4 |
| RP11-94B19.7 |
| RP11-883A18.3 |
| ZNF488 |
| CXADRP3 |
| LINC01029 |
| LINC00668 |
| GACAT2 |
| CTD-2515C13.2 |
| RP11-94B19.2 |
| RP11-703M24.5 |
| FBXO36P1 |
| RP11-640I15.1 |
| RP11-41O4.2 |
| RP11-484N16.1 |
| RP11-344E13.4 |
| RP11-874J12.4 |
| RP11-329L6.2 |
| RP11-91I8.3 |
| RP11-149I2.4 |
| CTD-2515C13.1 |
| RP11-650P15.1 |
| AL161645.1 |
| RP11-1007I13.4 |
| RP11-856M7.6 |
| DSG1-AS1 |
| RP11-94B19.6 |
| CTD-2200P10.1 |
| RP6-114E22.1 |
| RP11-744K17.2 |
| ZNF793-AS1 |
| RP11-806H10.4 |
| AC079466.1 |
| CTD-2171N6.1 |
| LINC01532 |
| RP11-800A18.4 |
| RP11-384O8.1 |
| AC006126.4 |
| RP11-456O19.2 |
| PTGES3L |
| AC005256.1 |
| RP11-127I20.5 |
| RP11-666A8.9 |
| RP11-861E21.1 |
| CTB-129P6.11 |
| CTD-2357A8.3 |
| RP11-449J21.5 |
| RP11-411B10.2 |
| RP11-15A1.2 |
| LCN6 |
| AC007773.2 |
| RP11-209M4.1 |
| CTD-2162K18.4 |
| RP11-397A16.1 |
| RP13-890H12.2 |
| RPL23AP77 |
| KC6 |
| CTD-2008L17.1 |
| RP11-244M2.1 |
| CTC-260E6.6 |
| CTC-296K1.4 |
| CTC-265F19.2 |
| RP11-636O21.1 |
| CTD-2319I12.2 |
| CTD-2265O21.3 |
| AC010641.1 |
| CTC-296K1.3 |
| BNIP3P16 |
| RP1-193H18.3 |
| RP11-973H7.4 |
| RP11-820I16.2 |
| CTB-55O6.4 |
| LDLRAD4-AS1 |
| AC002116.7 |
| RP11-729L2.2 |
| AC024592.9 |
| LINC01539 |
| CTD-2540B15.6 |
| RP11-64C12.3 |
| RP4-604K5.3 |
| HMGB2P1 |
| RUNDC3A-AS1 |
| C19orf83 |
| RP11-2N1.2 |
| AC004221.2 |
| RP11-154H12.3 |
| SMIM22 |
| CTD-2291D10.4 |
| SNX6P1 |
| RP11-255H23.4 |
| CTD-2620I22.3 |
| MAGEA9 |
| BNIP3P30 |
| RP11-388K12.3 |
| AC093063.2 |
| GABRQ |
| SLC6A14 |
| CTD-2561J22.5 |
| FKBP4P6 |
| RP11-420K14.2 |
| BNIP3P27 |
| SSX2B |
| CTD-2587H19.2 |
| IFNL3P1 |
| AC006262.5 |
| CTB-52I2.3 |
| AC068499.10 |
| LINC00664 |
| RP11-247A12.7 |
| VN1R87P |
| RP1-102K2.8 |
| CITF22-62D4.1 |
| RP11-138H11.1 |
| PNMA6B |
| CSAG2 |
| CSAG3 |
| CT45A1 |
| RP5-907D15.4 |
| CTD-2192J16.26 |
| MRPS17P1 |
| ERVV-2 |
| CTC-457E21.9 |
| AC005932.1 |
| CTC-513N18.6 |
| CTD-3187F8.14 |
| AC003682.17 |
| CTC-246B18.10 |
| CTD-2105E13.15 |
| CTB-92J24.3 |
| VN1R85P |
| CTD-2542C24.3 |
| SPIB |
| LINC01224 |
| CYP4F23P |
| ERVV-1 |
| CT45A10 |
| AC005197.2 |
| CTD-2542C24.2 |
| SSX4B |
| CTB-180A7.3 |
| RP4-621N11.2 |
| RP11-193M21.1 |
| RP3-333A15.2 |
| RP11-797A18.6 |
| SPACA6P-AS |
| RP3-426I6.5 |
| RP11-932O9.10 |
| RP11-96H17.3 |
| RP11-276H19.2 |
| RP11-178L8.7 |
| RP11-685G9.4 |
| RP11-1070N10.7 |
| RP4-761J14.9 |
| LA16c-380H5.3 |
| LA16c-380H5.4 |
| MTRNR2L11 |
| RP11-572O17.1 |
| RP11-2L8.2 |
| SMC2-AS1 |
| RP11-109M17.2 |
| RP11-92C4.6 |
| RP11-796E10.1 |
| CTC-451A6.5 |
| CTD-2134P3.2 |
| RP11-116B13.1 |
| bP-21201H5.1 |
| IGHV3-30 |
| RP11-359E10.1 |
| RP11-105N14.1 |
| RP11-344N17.15 |
| RP11-385F7.1 |
| DPRXP6 |
| C17orf50 |
| LINC00221 |
| LL22NC03-N64E9.1 |
| CTA-293F17.1 |
| RP11-642C5.1 |
| RP11-430G17.3 |
| RP11-138A9.1 |
| RP11-478H13.4 |
| RP11-465O11.1 |
| RP11-350D17.3 |
| RP11-382D12.2 |
| RP11-603B24.6 |
| BNIP3P17 |
| RP11-230C9.2 |
| RP11-435O5.4 |
| RP11-141C7.3 |
| RP11-1259L22.2 |
| MROH7-TTC4 |
| RP1-111B22.3 |
| RP11-269M20.3 |
| CTD-2201E18.5 |
| RP1-63M2.5 |
| RP4-789D17.5 |
| RP11-44N12.5 |
| RP11-443B20.1 |
| RP11-439M11.1 |
| RP11-245G13.2 |
| RP11-28H5.2 |
| RP4-736L20.3 |
| RP11-42O15.3 |
| RP11-337N6.1 |
| RP11-471M2.3 |
| CTB-40H15.4 |
| RP11-284F21.9 |
| RP11-332J15.4 |
| RP11-168F9.2 |
| RP4-665J23.4 |
| RP4-555D20.4 |
| RP11-177G23.2 |
| CTD-2544H17.2 |
| CTD-2035E11.5 |
| RP11-350N15.6 |
| RP11-481J13.1 |
| RP11-157F20.3 |
| RP11-22L13.1 |
| KB-1958F4.2 |
| LINC00551 |
| TPTE2P2 |
| RP11-573G6.10 |
| RP11-44N11.2 |
| RP11-666A8.12 |
| RP11-108M9.6 |
| RP11-54O7.16 |
| RP11-689C9.1 |
| RP1-86C11.7 |
| RP11-284J1.1 |
| RP11-54O7.17 |
| RP11-29P20.1 |
| RP11-254F7.4 |
| RP11-462G22.2 |
| RP11-461M2.2 |
| AFAP1-AS1 |
| RP11-362F19.3 |
| RP11-190C22.8 |
| RP11-190A12.8 |
| RP11-145M9.5 |
| RP11-534C12.1 |
| RP4-753F5.1 |
| RP11-142A22.4 |
| RP11-799N11.1 |
| RP11-357H14.17 |
| RP11-725P16.2 |
| RP11-674N23.4 |
| RP11-141C7.4 |
| RP11-368I23.3 |
| RP11-445P19.3 |
| RP4-593H12.1 |
| RP11-561I11.4 |
| LL22NC03-N14H11.1 |
| RP11-309L24.4 |
| CTA-246H3.12 |
| CTD-2308L22.1 |
| RP11-118K6.3 |
| RP11-336K24.12 |
| RP11-77E14.2 |
| RP11-334G22.1 |
| RP11-216L13.19 |
| AP000569.9 |
| RP11-165A20.3 |
| RP11-416N2.4 |
| LYPD4 |
| RP11-350J20.12 |
| RP11-525A16.4 |
| RP11-108L7.15 |
| RP11-368I23.4 |
| RP11-339B21.10 |
| AC006946.16 |
| RP11-107N15.1 |
| CTA-384D8.34 |
| RP11-445N20.3 |
| RP11-38M8.1 |
| RP11-314B1.2 |
| TM4SF19-TCTEX1D2 |
| RP11-757A13.1 |
| RP11-480C22.1 |
| AC006946.17 |
| RP11-1399P15.1 |
| RP11-313P22.1 |
| RP11-126K1.9 |
| RP11-1136G4.2 |
| ABC7-481722F1.1 |
| CH17-360D5.3 |
| RP11-81H14.1 |
| AL078471.5 |
| RP11-77K12.10 |
| RP11-973D8.5 |
| FAM27E3 |
| RP11-407N8.6 |
| RP11-324E6.10 |
| RP11-670E13.6 |
| RP11-115D19.3 |
| RP11-566K19.6 |
| XXbac-BPG294E21.9 |
| RP11-19G24.1 |
| RP11-463J17.2 |
| RP11-104O19.4 |
| RP11-94I2.4 |
| TPTE |
| RP11-386J22.3 |
| RP11-972P1.10 |
| RP11-128A17.2 |
| RP11-323P17.2 |
| RP1-62D2.4 |
| RP11-423G4.10 |
| RP11-122K13.15 |
| RP11-71L14.4 |
| RP11-86K22.2 |
| RP5-1057I20.6 |
| KRTAP7-1 |
| LA16c-358B7.4 |
| CTD-2129N1.1 |
| LINC01297 |
| RP11-320G10.1 |
| RP11-49I11.4 |
| RP11-65I12.1 |
| RP11-12A20.7 |
| RP5-864K19.6 |
| UG0898H09 |
| HIST1H2AH |
| RP11-61O11.1 |
| LA16c-329F2.2 |
| RP11-32D4.1 |
| RP11-427J23.1 |
| RP11-54H7.4 |
| RP11-370I10.10 |
| FGD5P1 |
| RP5-907C10.4 |
| RP11-568J23.8 |
| FCGBP |
| RP11-126O1.6 |
| RP11-334C17.6 |
| CTC-1337H24.4 |
| RP5-1009E24.8 |
| XKR5 |
| FLJ16779 |
| RP5-967N21.11 |
| RP11-111E14.2 |
| LINC00162 |
| RP11-90L1.8 |
| PRSS3P2 |
| RP11-1055B8.9 |
| UHRF1 |
| FLJ36000 |
| MIR6835 |
| LLNLR-268E12.1 |
| LINC00540 |
| AC007163.1 |
| WI2-2118C23.2 |
| RP11-323F24.3 |
| AP001505.10 |
| RP11-115D19.2 |
| RP11-381O6.1 |
| RP11-461A8.5 |
| RP11-381E24.4 |
| CTD-2105E13.16 |
| CH17-360D5.2 |
| RP5-875H18.9 |
| RP1-81D8.5 |
| HIST1H4E |
| AC019129.2 |
| HYDIN2 |
| RP3-453C12.15 |
| HIST1H4D |
| SPANXA2-OT1 |
| RP4-550H1.7 |
| RP11-445F12.1 |
| RP4-794I6.4 |
| RP11-843B15.4 |
| RP11-462D18.4 |
| CH17-408M7.1 |
| CTC-492K19.7 |
| CTD-3035K23.6 |
| CTD-2095E4.5 |
| RP11-545P7.9 |
| RP4-616B8.6 |
| NEFL |
| AC005363.11 |
| RNVU1-20 |
| CDKN2B-AS |
| AP003900.6 |
| CH17-13I23.3 |
| RP11-693J15.6 |
| SRD5A2 |
| RP5-851M4.1 |
| RP11-167B3.3 |
| RDM1 |
| RP5-984P4.6 |
| RP11-283G6.6 |
| SSTR3 |
| RP13-766D20.4 |
| HIST1H3C |
| CTD-2311B13.9 |
| ZNF229 |
| RP11-626G11.6 |
| RP11-255P5.3 |
| HIST1H2AB |
| RP5-903E17.2 |
| AC068831.16 |
| RP11-108K14.12 |
| CHMP1B2P |
| RP11-146E13.5 |
| TBC1D3E |
| RP13-608F4.5 |
| CTC-543D15.8 |
| CTD-2318O12.1 |
| HIST1H4A |
| RP11-159D12.11 |
| RP11-282A11.3 |
| HIST1H4B |
| RP5-1059L7.1 |
| LA16c-352F7.1 |
| RP11-498C9.4 |
| FP325331.1 |
| CTD-2342N23.1 |
| BANCR |
| RP11-313L6.2 |
| RP5-849H19.3 |
| LL22NC03-22D1.1 |
| RP11-1193F23.1 |
| RP11-649A18.3 |
| RP11-81K2.2 |
| CTC-260E6.3 |
| CTC-270D5.1 |
| KB-1742H10.3 |
| RP11-477I4.4 |
| RP11-345K9.2 |
| RP11-749I16.3 |
| RP5-991G20.6 |
| bP-21264C1.1 |
| GPR1-AS |
| RP11-2K6.1 |
| RP11-158L12.4 |
| RP11-491F9.3 |
| RP11-226M10.3 |
| RP1-257C22.2 |
| KB-176G8.1 |
| AC003973.3 |
| RP11-84N19.1 |
| RP11-635L1.2 |
| bP-2171C21.4 |
| RP11-91I8.2 |
| CTC-251I16.1 |
| RP11-676B18.1 |
| FAM230C |
| CTB-96E2.6 |
| AL845472.1 |
| RP4-570O12.3 |
| bP-2189O9.3 |
| RP11-235C23.6 |
| RP11-254F19.4 |
| RP11-177G23.1 |
| RP11-70L8.5 |
| RP11-185E8.2 |
| RP11-31H5.2 |
| RP11-485M7.2 |
| CTA-280A3.2 |
| bP-21264C1.3 |
| LINC00552 |
| RP11-60A14.1 |
| RP11-555K2.2 |
| RP11-20F18.1 |
| C8orf87 |
| CTD-2026A21.1 |
| RP11-286N22.16 |
| RP11-483E17.1 |
| FLJ42393 |
| AC021451.1 |
| RP11-145E17.3 |
| RP11-76C10.6 |
| CTD-2123J17.2 |
| CTD-2095E4.3 |
| RP11-296E3.2 |
| RP11-324H9.1 |
| CTA-796E4.5 |
| CTD-2525P14.5 |
| RP11-555K2.1 |
| CTC-260E6.4 |
| MGC39584 |
| RP11-158L12.6 |
| AC055733.1 |
| AC006548.28 |
| RP11-244B22.10 |
| RP11-483I13.6 |
| CTB-193M12.5 |
| RP11-560F18.1 |
| RP11-556H2.1 |
| RP11-643A5.3 |
| CITF22-92A6.2 |
| CH507-528H12.1 |
| ASAP1-IT2 |
| LINC01242 |
| LINC01452 |
| FOXCUT |
| LINC00628 |
| RP11-900F13.3 |
| BLACAT1 |
