# Supplementary material for: Comprehensive multiomics analysis of cuproptosis-related gene characteristics in hepatocellular carcinoma
Source: Front Genet. 2022 Sep 6;13:942387. doi: 10.3389/fgene.2022.942387 (PMC9486098; doi:10.3389/fgene.2022.942387)
Supplement: Supplementary file 9 [file Table5.DOCX]

Table S5. Comparison of clinicopathological features before and after interpolation.

|  | Before-interpolation (No.) | After-interpolation (No.) | P.value |
| --- | --- | --- | --- |
| Variables | 370 | 370 |  |
| OS (mean(sd)) | 26.35 (23.89) | 26.35 (23.89) | 1 |
| Status (mean(sd)) | 0.35 (0.48) | 0.35 (0.48) | 1 |
| Age (mean(sd)) | 0.52 (0.5) | 0.52 (0.5) | 1 |
| Stage (mean(sd)) | 0.26 (0.44) | 0.27 (0.44) | 0.76 |
| T stage (mean(sd)) | 0.26 (0.44) | 0.26 (0.44) | 0.97 |
| Histologic Grade (mean(sd)) | 0.36 (0.48) | 0.36 (0.48) | 0.99 |
| Hepatitis (mean(sd)) | 0.44 (0.5) | 0.44 (0.5) | 0.98 |
| Surgical Margin (mean(sd)) | 0.11 (0.31) | 0.11 (0.32) | 0.89 |
| Gender (mean(sd)) | 0.67 (0.47) | 0.67 (0.47) | 1 |
| Vascular Invasion (mean(sd)) | 0.34 (0.48) | 0.38 (0.49) | 0.31 |

Group information: Status (“Alive” = 0, “Dead” = 1), Age (“≤ 60y” = 0, “> 60y” = 1), Stage (“Stage I-II” = 0, “Stage III-IV” = 1), T stage (“T1-T2” = 0, “T3-T4” = 1), Histological grade (“G1-G2” = 0, “G3-G4” =1), Hepatitis (“Hepatitis (-)” = 0, “Hepatitis (+)” = 1), Surgical margin (“R0” = 0, “R1-R2” = 1), Gender (“female” = 0, “male” = 1) and Vascular invasion (“None” = 0, “Macro-Micro” =1 ).
