# Supplementary material for: Comprehensive multiomics analysis of cuproptosis-related gene characteristics in hepatocellular carcinoma
Source: Front Genet. 2022 Sep 6;13:942387. doi: 10.3389/fgene.2022.942387 (PMC9486098; doi:10.3389/fgene.2022.942387)
Supplement: Supplementary file 10 [file Table3.DOCX]

Table S3. The results of univariate and multivariate Cox analyses of the 168 prognostic DEGs.

|  | Univariate Cox analysis | | | Multivariate Cox analysis | | |
| --- | --- | --- | --- | --- | --- | --- |
| Gene | HR | CI95 | P | HR | CI95 | P |
| AC006372.5 | 1.9 | 1.28-2.83 | 0.002 | 0.64 | 0.18-2.29 | 0.492 |
| AC008427.2 | 12.22 | 3.28-45.47 | 0 | 1.16 | 0.05-29.44 | 0.928 |
| AC012512.1 | 2.93 | 1.9-4.5 | 0 | 6.79 | 1.64-28.13 | 0.008 |
| AC018641.7 | 3.06 | 2.16-4.35 | 0 | 2.62 | 0.64-10.81 | 0.182 |
| AC068858.1 | 1.05 | 0.41-2.73 | 0.913 | NA | NA | NA |
| AC087499.5 | 2.48 | 1.18-5.2 | 0.016 | 0.74 | 0.09-6.5 | 0.789 |
| AC090954.5 | 1.86 | 1.17-2.94 | 0.008 | 0.96 | 0.26-3.5 | 0.95 |
| AC111155.1 | 1.96 | 1.15-3.34 | 0.013 | 2.01 | 0.72-5.61 | 0.185 |
| AC114776.1 | 5.34 | 1.73-16.46 | 0.004 | 0.83 | 0.04-16.76 | 0.905 |
| ANXA2R | 1.4 | 1.02-1.91 | 0.035 | 0.52 | 0.24-1.12 | 0.096 |
| AP000619.5 | 4.35 | 1.68-11.26 | 0.002 | 0.04 | 0-3.15 | 0.149 |
| AP001601.2 | 1.2 | 0.76-1.91 | 0.428 | NA | NA | NA |
| ASL | 0.94 | 0.75-1.18 | 0.618 | NA | NA | NA |
| ATP8A2P1 | 1.54 | 1.04-2.29 | 0.031 | 0.72 | 0.22-2.31 | 0.579 |
| B3GALT2 | 1.2 | 0.9-1.6 | 0.208 | NA | NA | NA |
| BCL2L15 | 1.47 | 0.9-2.41 | 0.121 | NA | NA | NA |
| BTNL10 | 1.65 | 0.78-3.49 | 0.188 | NA | NA | NA |
| BTNL9 | 0.63 | 0.48-0.84 | 0.002 | 1.3 | 0.71-2.37 | 0.392 |
| C2orf83 | 26.01 | 3.72-181.62 | 0.001 | 223.85 | 0.77-65242.8 | 0.062 |
| CABYR | 1.32 | 1.14-1.53 | 0 | 1.2 | 0.83-1.74 | 0.328 |
| CACNA1B | 3.9 | 2.05-7.42 | 0 | 0.9 | 0.04-18.32 | 0.945 |
| CACNG4 | 1.08 | 0.92-1.27 | 0.332 | NA | NA | NA |
| CCNB1 | 1.63 | 1.34-1.98 | 0 | 0.62 | 0.23-1.73 | 0.365 |
| CCT7P2 | 2.34 | 1.22-4.52 | 0.011 | 0.03 | 0-0.38 | 0.006 |
| CD7 | 1.19 | 1.01-1.41 | 0.04 | 0.71 | 0.45-1.1 | 0.123 |
| CDH10 | 1.78 | 1.34-2.37 | 0 | 2.46 | 1.01-5.99 | 0.048 |
| CLDN6 | 1.68 | 1.25-2.25 | 0.001 | 2.69 | 1.36-5.33 | 0.004 |
| CNFN | 1.06 | 0.89-1.27 | 0.494 | NA | NA | NA |
| COL11A2 | 1.02 | 0.68-1.54 | 0.907 | NA | NA | NA |
| CRH | 2.22 | 1.47-3.36 | 0 | 1.21 | 0.03-42.6 | 0.917 |
| CST8 | 2.19 | 1.03-4.67 | 0.042 | 1.72 | 0.18-16.4 | 0.638 |
| CTB.1I21.1 | 1.99 | 1.3-3.05 | 0.002 | 1 | 0.31-3.24 | 0.995 |
| CTC.508F8.1 | 2.38 | 0.91-6.26 | 0.078 | NA | NA | NA |
| CTD.2147F2.2 | 3.03 | 0.53-17.26 | 0.212 | NA | NA | NA |
| CTD.2311B13.9 | 26.18 | 4.72-145.18 | 0 | 2.85 | 0-1904.95 | 0.752 |
| CTD.2510F5.4 | 1.55 | 1.29-1.87 | 0 | 1.02 | 0.44-2.35 | 0.967 |
| CTXN2 | 14.37 | 0.78-265.62 | 0.073 | NA | NA | NA |
| DCAF8L1 | 1.73 | 1.31-2.29 | 0 | 1.56 | 0.67-3.64 | 0.301 |
| DIRAS2 | 1.81 | 1.37-2.39 | 0 | 1.96 | 0.91-4.23 | 0.088 |
| DRGX | 1.44 | 1.03-2 | 0.031 | 1.72 | 0.81-3.69 | 0.159 |
| DYNLL1P4 | 1.79 | 0.79-4.07 | 0.164 | NA | NA | NA |
| ECEL1 | 1.02 | 0.88-1.19 | 0.79 | NA | NA | NA |
| EPO | 1.33 | 1.18-1.51 | 0 | 1.62 | 1.15-2.29 | 0.006 |
| EYA1 | 1.57 | 1.01-2.44 | 0.044 | 0.6 | 0.14-2.64 | 0.499 |
| FABP5 | 1.48 | 1.2-1.84 | 0 | 0.81 | 0.45-1.46 | 0.488 |
| FAM163B | 0.83 | 0.64-1.08 | 0.162 | NA | NA | NA |
| FAM19A5 | 0.99 | 0.86-1.14 | 0.888 | NA | NA | NA |
| FAM27C | 1.69 | 1.21-2.36 | 0.002 | 1.61 | 0.59-4.38 | 0.352 |
| FCN3 | 0.82 | 0.69-0.97 | 0.024 | 0.66 | 0.47-0.91 | 0.011 |
| FOXG1 | 1.34 | 0.75-2.39 | 0.322 | NA | NA | NA |
| FOXH1 | 1.41 | 1.05-1.89 | 0.022 | 1.76 | 0.7-4.4 | 0.227 |
| FOXI2 | 2.39 | 0.22-26.46 | 0.478 | NA | NA | NA |
| FP325331.1 | 12.07 | 3.38-43.07 | 0 | 0.63 | 0.02-16.66 | 0.782 |
| G6PD | 1.6 | 1.38-1.86 | 0 | 0.75 | 0.35-1.6 | 0.455 |
| GAGE1 | 1.53 | 1.1-2.14 | 0.012 | 0.3 | 0.07-1.36 | 0.119 |
| GAGE2A | 1.5 | 1.27-1.78 | 0 | 1.1 | 0.56-2.18 | 0.778 |
| GALNT3 | 1.05 | 0.78-1.42 | 0.755 | NA | NA | NA |
| GNAO1 | 0.96 | 0.77-1.2 | 0.74 | NA | NA | NA |
| GNGT1 | 2.09 | 1.33-3.27 | 0.001 | 0.16 | 0.03-0.8 | 0.026 |
| GPR123.AS1 | 14.89 | 1.15-191.88 | 0.038 | 248.23 | 0.07-908343.04 | 0.188 |
| GRAPL | 4.75 | 0.14-165.76 | 0.39 | NA | NA | NA |
| HLA.V | 1.53 | 0.95-2.45 | 0.078 | NA | NA | NA |
| HOXA7 | 3.05 | 1.32-7.03 | 0.009 | 6.47 | 1.25-33.45 | 0.026 |
| HS3ST5 | 3.18 | 1.53-6.61 | 0.002 | 4.5 | 0.52-39.18 | 0.173 |
| IFIT1 | 0.92 | 0.76-1.12 | 0.403 | NA | NA | NA |
| IGKC | 0.95 | 0.88-1.03 | 0.238 | NA | NA | NA |
| IL20RA | 1.31 | 1.03-1.68 | 0.028 | 0.92 | 0.5-1.71 | 0.801 |
| ITGAM | 1.31 | 1.05-1.62 | 0.015 | 0.39 | 0.19-0.84 | 0.015 |
| KB.1980E6.2 | 18.99 | 3.84-94 | 0 | 131.04 | 0.14-122326 | 0.162 |
| KBTBD11 | 0.8 | 0.62-1.02 | 0.073 | NA | NA | NA |
| KIF24 | 1.63 | 1.09-2.42 | 0.016 | 0.02 | 0-0.08 | 0 |
| KIF2C | 1.71 | 1.42-2.07 | 0 | 1.79 | 0.46-6.91 | 0.399 |
| KISS1R | 1.49 | 1.11-1.98 | 0.007 | 0.67 | 0.31-1.46 | 0.319 |
| LA16c.312E8.2 | 10.73 | 3.63-31.71 | 0 | 7.22 | 0.58-90.37 | 0.125 |
| LCN15 | 1.24 | 0.84-1.85 | 0.276 | NA | NA | NA |
| LHX5 | 9.96 | 1.29-76.64 | 0.027 | 0.41 | 0-3858.94 | 0.848 |
| LINC00462 | 3.01 | 1.87-4.85 | 0 | 3.02 | 0.5-18.41 | 0.23 |
| LINC00501 | 4.61 | 2.64-8.05 | 0 | 0.39 | 0.06-2.74 | 0.347 |
| LINC00559 | 225.65 | 2.52-20231.24 | 0.018 | 18.26 | 0-656909.72 | 0.587 |
| LINC01296 | 12.32 | 4.62-32.82 | 0 | 1.2 | 0.08-17.26 | 0.894 |
| LIPM | 1.22 | 0.84-1.77 | 0.298 | NA | NA | NA |
| MAGEB10 | 2.97 | 1.29-6.85 | 0.011 | 0.44 | 0.02-10.95 | 0.615 |
| MCM7 | 1.52 | 1.21-1.9 | 0 | 1.4 | 0.62-3.2 | 0.419 |
| MDGA1 | 1.33 | 1.02-1.74 | 0.037 | 1.61 | 0.85-3.05 | 0.141 |
| MEP1AP4 | 10.67 | 2.35-48.54 | 0.002 | 30.83 | 1.82-522.12 | 0.018 |
| MIR100HG | 0.56 | 0.33-0.94 | 0.027 | 0.63 | 0.23-1.72 | 0.371 |
| MMP3 | 1.64 | 1.2-2.22 | 0.002 | 1.34 | 0.72-2.49 | 0.362 |
| MSC | 1.24 | 1.11-1.39 | 0 | 1.45 | 1.06-1.99 | 0.02 |
| MYLK2 | 2.29 | 1.48-3.56 | 0 | 1.06 | 0.1-10.87 | 0.962 |
| NBPF4 | 21.3 | 7.11-63.79 | 0 | 0.73 | 0.01-68.07 | 0.894 |
| NCAPD2P1 | 1.6 | 1.2-2.13 | 0.002 | 1.31 | 0.59-2.92 | 0.502 |
| NCAPG | 1.72 | 1.39-2.13 | 0 | 0.5 | 0.12-2.08 | 0.339 |
| NLRP5 | 3.39 | 1.56-7.4 | 0.002 | 3.1 | 0.13-71.44 | 0.48 |
| NPTX2 | 1.14 | 1.01-1.28 | 0.033 | 0.96 | 0.72-1.27 | 0.759 |
| NR0B1 | 1.57 | 1.32-1.88 | 0 | 0.87 | 0.48-1.58 | 0.643 |
| NYX | 19.72 | 1.09-356.81 | 0.044 | 0.5 | 0-3928.88 | 0.88 |
| P2RY6 | 1.71 | 1.35-2.17 | 0 | 1.43 | 0.62-3.33 | 0.405 |
| PDCD1 | 1 | 0.81-1.24 | 0.994 | NA | NA | NA |
| PDE6A | 42.69 | 9.64-189.01 | 0 | 36.32 | 0.37-3591.67 | 0.125 |
| PEG3 | 1.12 | 0.97-1.28 | 0.122 | NA | NA | NA |
| PFN2 | 1.42 | 1.22-1.65 | 0 | 1.52 | 1.05-2.22 | 0.028 |
| PGM5P4 | 0.45 | 0.11-1.84 | 0.267 | NA | NA | NA |
| PKIB | 1.37 | 1.18-1.6 | 0 | 1.11 | 0.73-1.69 | 0.63 |
| PLG | 0.87 | 0.78-0.98 | 0.02 | 0.82 | 0.52-1.3 | 0.393 |
| PNMA6B | 1.31 | 0.98-1.76 | 0.071 | NA | NA | NA |
| PRRT3.AS1 | 1.53 | 1.24-1.89 | 0 | 0.78 | 0.47-1.3 | 0.342 |
| PSMC3IP | 2 | 1.48-2.72 | 0 | 1.75 | 0.45-6.86 | 0.419 |
| RAB27B | 1.19 | 0.92-1.54 | 0.18 | NA | NA | NA |
| RASL11A | 1.03 | 0.84-1.25 | 0.777 | NA | NA | NA |
| RGS20 | 3.15 | 1.8-5.52 | 0 | 0.66 | 0.09-4.67 | 0.678 |
| RHEBL1 | 2.22 | 1.54-3.19 | 0 | 2.28 | 0.71-7.35 | 0.167 |
| RIMKLA | 2.16 | 1.38-3.37 | 0.001 | 0.82 | 0.23-2.98 | 0.769 |
| RN7SL368P | 4.59 | 1.95-10.83 | 0 | 26.94 | 2.66-272.91 | 0.005 |
| RNA5SP154 | 1.23 | 0.83-1.82 | 0.304 | NA | NA | NA |
| RNF125 | 0.81 | 0.62-1.05 | 0.107 | NA | NA | NA |
| RP1.140K8.2 | 3.62 | 1.75-7.52 | 0.001 | 2.85 | 0.53-15.22 | 0.22 |
| RP1.63M2.5 | 3.6 | 1.44-8.98 | 0.006 | 7.18 | 1.14-45.39 | 0.036 |
| RP11.159H10.3 | 4.78 | 1.74-13.18 | 0.002 | 7.31 | 0.71-75.12 | 0.094 |
| RP11.178L8.7 | 1.27 | 1.01-1.61 | 0.044 | 0.69 | 0.35-1.39 | 0.305 |
| RP11.204C23.1 | 1.96 | 1.24-3.09 | 0.004 | 3.01 | 0.52-17.52 | 0.221 |
| RP11.22L13.1 | 6.79 | 2.6-17.73 | 0 | 26.19 | 0.83-827.62 | 0.064 |
| RP11.235G24.1 | 0.99 | 0.62-1.58 | 0.961 | NA | NA | NA |
| RP11.265N7.2 | 1.65 | 0.5-5.45 | 0.414 | NA | NA | NA |
| RP11.313L6.2 | 2.92 | 1.27-6.72 | 0.011 | 3.22 | 0.32-32.66 | 0.322 |
| RP11.366H4.1 | 1.97 | 1.32-2.92 | 0.001 | 1.88 | 0.51-6.9 | 0.342 |
| RP11.383J24.1 | 1.61 | 1.33-1.96 | 0 | 1.06 | 0.56-2.03 | 0.852 |
| RP11.417E7.2 | 1 | 0.66-1.51 | 0.986 | NA | NA | NA |
| RP11.427J23.1 | 1.09 | 0.66-1.78 | 0.745 | NA | NA | NA |
| RP11.428P16.2 | 31.76 | 5.91-170.6 | 0 | 0.37 | 0-262.14 | 0.764 |
| RP11.451G4.3 | 2.4 | 1.19-4.81 | 0.014 | 1.05 | 0.14-8.13 | 0.96 |
| RP11.473A10.2 | 2.79 | 1.61-4.85 | 0 | 1.14 | 0.13-9.98 | 0.906 |
| RP11.478H13.4 | 1.44 | 1.09-1.91 | 0.01 | 0.9 | 0.37-2.16 | 0.806 |
| RP11.60L3.1 | 5.01 | 1.03-24.42 | 0.046 | 0.54 | 0.01-35.21 | 0.775 |
| RP11.705O24.1 | 36.77 | 8.75-154.44 | 0 | 33.93 | 0.42-2770.85 | 0.117 |
| RP11.76C10.5 | 1.47 | 1.11-1.95 | 0.007 | 1.46 | 0.63-3.4 | 0.383 |
| RP11.7I15.4 | 1.35 | 0.96-1.89 | 0.084 | NA | NA | NA |
| RP11.874J12.4 | 2.36 | 1.76-3.16 | 0 | 0.89 | 0.32-2.49 | 0.822 |
| RP11.88I21.1 | 1.78 | 1.25-2.54 | 0.001 | 2.93 | 0.52-16.41 | 0.222 |
| RP11.96H17.1 | 3.22 | 1.47-7.08 | 0.004 | 4.61 | 0.33-65.26 | 0.259 |
| RP11.99L13.2 | 1.51 | 0.95-2.41 | 0.083 | NA | NA | NA |
| RP13.16H11.2 | 1.63 | 1.24-2.13 | 0 | 1.33 | 0.38-4.6 | 0.652 |
| RP4.659I19.1 | 1.56 | 0.95-2.55 | 0.077 | NA | NA | NA |
| RP5.1096J16.1 | 1.73 | 1.3-2.3 | 0 | 0.99 | 0.38-2.61 | 0.989 |
| RPSAP64 | 0.98 | 0.36-2.69 | 0.967 | NA | NA | NA |
| SBSPON | 1.13 | 0.89-1.43 | 0.323 | NA | NA | NA |
| SEPT14 | 1.79 | 1.36-2.37 | 0 | 0.2 | 0.05-0.9 | 0.036 |
| SLC22A31 | 1.02 | 0.92-1.14 | 0.647 | NA | NA | NA |
| SLC2A2 | 0.83 | 0.74-0.92 | 0.001 | 1.18 | 0.79-1.77 | 0.412 |
| SLC6A2 | 0.99 | 0.85-1.15 | 0.886 | NA | NA | NA |
| SNORA77 | 2.35 | 0.44-12.56 | 0.317 | NA | NA | NA |
| SPINK4 | 1.43 | 1.16-1.75 | 0.001 | 1.28 | 0.7-2.35 | 0.415 |
| SUMO1P1 | 1.65 | 1.05-2.59 | 0.029 | 0.44 | 0.04-4.5 | 0.491 |
| TAS2R2P | 4.36 | 1.86-10.24 | 0.001 | 0.36 | 0-26.69 | 0.639 |
| TCF24 | 3.37 | 1.45-7.86 | 0.005 | 0.5 | 0.01-18.4 | 0.707 |
| TEX15 | 2.93 | 1.85-4.65 | 0 | 7.21 | 1.47-35.26 | 0.015 |
| TGFB2 | 1.21 | 0.97-1.52 | 0.089 | NA | NA | NA |
| TM4SF1 | 1.18 | 0.99-1.41 | 0.067 | NA | NA | NA |
| TMEM154 | 0.86 | 0.57-1.29 | 0.467 | NA | NA | NA |
| TMTC2 | 1.41 | 0.97-2.05 | 0.073 | NA | NA | NA |
| TRIM16 | 1.33 | 1.14-1.56 | 0 | 0.6 | 0.26-1.36 | 0.22 |
| TRIM16L | 1.29 | 1.11-1.5 | 0.001 | 1.33 | 0.66-2.68 | 0.425 |
| TTK | 1.87 | 1.49-2.34 | 0 | 4.76 | 1.25-18.1 | 0.022 |
| UAP1L1 | 1.48 | 1.24-1.77 | 0 | 1.11 | 0.62-1.98 | 0.731 |
| VSIG1 | 1.21 | 1-1.45 | 0.045 | 0.97 | 0.63-1.47 | 0.869 |
| XCL1 | 1.15 | 0.88-1.5 | 0.31 | NA | NA | NA |
| XXbac.BPG308K3.5 | 2.17 | 1.04-4.53 | 0.04 | 2.02 | 0.42-9.76 | 0.384 |
| YJEFN3 | 1.48 | 1.11-1.97 | 0.007 | 0.3 | 0.13-0.67 | 0.003 |
| ZFP42 | 2.3 | 1.1-4.84 | 0.028 | 0 | 0-10.14 | 0.167 |
