# Supplementary material for: Comprehensive multiomics analysis of cuproptosis-related gene characteristics in hepatocellular carcinoma
Source: Front Genet. 2022 Sep 6;13:942387. doi: 10.3389/fgene.2022.942387 (PMC9486098; doi:10.3389/fgene.2022.942387)
Supplement: Supplementary file 11 [file Table4.DOCX]

Table S4. Results of correlation analyses between 19 prognostic genes and drugs.

| Gene | Drug | cor | P value |
| --- | --- | --- | --- |
| GNGT1 | SR16157 | 0.52182 | 1.91E-05 |
| PFN2 | Imexon | -0.49561 | 5.66E-05 |
| PFN2 | Cyclophosphamide | -0.49327 | 6.22E-05 |
| SEPT14 | Isotretinoin | 0.491264 | 6.73E-05 |
| SEPT14 | Fluphenazine | 0.487577 | 7.77E-05 |
| PFN2 | Nilotinib | -0.47964 | 0.000105 |
| ITGAM | Gemcitabine | 0.478987 | 0.000108 |
| EPO | Dexamethasone Decadron | 0.475249 | 0.000124 |
| SEPT14 | Irofulven | -0.4736 | 0.000132 |
| FCN3 | Hydrastinine HCl | 0.472866 | 0.000136 |
| PFN2 | Irofulven | 0.465801 | 0.000176 |
| SEPT14 | Imiquimod | 0.441457 | 0.000414 |
| GNGT1 | Epothilone B | -0.43739 | 0.000475 |
| SEPT14 | Megestrol acetate | 0.435413 | 0.000507 |
| PFN2 | LDK-378 | -0.43073 | 0.000592 |
| GNGT1 | Fulvestrant | 0.428276 | 0.000641 |
| PFN2 | Crizotinib | -0.42056 | 0.000821 |
| CDH10 | Mitomycin | 0.420166 | 0.000831 |
| PFN2 | Imatinib | -0.41862 | 0.000872 |
| MSC | Zoledronate | 0.416114 | 0.000944 |
| CLDN6 | bisacodyl, active ingredient of viraplex | 0.412979 | 0.001041 |
| YJEFN3 | Nelarabine | 0.411459 | 0.001091 |
| TEX15 | Carboplatin | 0.408656 | 0.001188 |
| PFN2 | Bafetinib | -0.40043 | 0.001523 |
| PFN2 | Palbociclib | -0.39611 | 0.00173 |
| PFN2 | By-Product of CUDC-305 | -0.39597 | 0.001738 |
| ITGAM | Hydroxyurea | 0.393712 | 0.001857 |
| PFN2 | Carmustine | -0.3913 | 0.001991 |
| MSC | Alvespimycin | -0.39032 | 0.002048 |
| MSC | Idelalisib | 0.389653 | 0.002088 |
| YJEFN3 | Elliptinium Acetate | 0.385074 | 0.002381 |
| TEX15 | Buthionine sulphoximine | 0.384439 | 0.002424 |
| ITGAM | Cytarabine | 0.383587 | 0.002483 |
| CDH10 | Cisplatin | 0.381277 | 0.00265 |
| GNGT1 | AFP464 | 0.371703 | 0.003454 |
| ITGAM | Fludarabine | 0.368638 | 0.003753 |
| TTK | Nelarabine | 0.36796 | 0.003822 |
| EPO | Fluphenazine | 0.363625 | 0.004292 |
| CDH10 | Valrubicin | 0.359286 | 0.004813 |
| TEX15 | Digoxin | 0.358632 | 0.004896 |
| MSC | By-Product of CUDC-305 | -0.35852 | 0.00491 |
| YJEFN3 | Raltitrexed | 0.357954 | 0.004984 |
| TEX15 | 3-Bromopyruvate (acid) | 0.352647 | 0.005718 |
| ITGAM | Triapine | 0.350847 | 0.005988 |
| PFN2 | Dimethylaminoparthenolide | -0.35037 | 0.006062 |
| FCN3 | Buthionine sulphoximine | 0.3488 | 0.006308 |
| PFN2 | Raloxifene | -0.34825 | 0.006397 |
| FCN3 | Parthenolide | 0.346725 | 0.006648 |
| KIF24 | AFP464 | -0.34664 | 0.006663 |
| TEX15 | Cisplatin | 0.344188 | 0.007086 |
| ITGAM | Digoxin | 0.343802 | 0.007154 |
| PFN2 | kahalide f | 0.341558 | 0.007565 |
| PFN2 | Simvastatin | 0.34135 | 0.007604 |
| ITGAM | Cladribine | 0.341154 | 0.007641 |
| MSC | Pazopanib | 0.340803 | 0.007708 |
| PFN2 | Dexrazoxane | -0.34013 | 0.007838 |
| TEX15 | Parthenolide | 0.339994 | 0.007864 |
| YJEFN3 | Dexamethasone Decadron | 0.339871 | 0.007887 |
| MSC | Palbociclib | -0.33867 | 0.008125 |
| YJEFN3 | Triethylenemelamine | 0.338027 | 0.008254 |
| ITGAM | Cisplatin | 0.337615 | 0.008337 |
| YJEFN3 | Irinotecan | 0.336929 | 0.008478 |
| ITGAM | Triethylenemelamine | 0.336098 | 0.008652 |
| TTK | Mithramycin | -0.3351 | 0.008865 |
| YJEFN3 | Decitabine | 0.334581 | 0.008978 |
| MSC | Pyrazoloacridine | -0.33342 | 0.009233 |
| GNGT1 | Elesclomol | 0.332913 | 0.009347 |
| PFN2 | Fluphenazine | -0.33291 | 0.009348 |
| ITGAM | 5-fluoro deoxy uridine 10mer | 0.331919 | 0.009574 |
| SEPT14 | Denileukin Diftitox Ontak | 0.331784 | 0.009605 |
| EPO | Oxaliplatin | 0.331769 | 0.009608 |
| MSC | Belinostat | -0.33136 | 0.009704 |
| YJEFN3 | Thiotepa | 0.330166 | 0.009985 |
| TEX15 | Dimethylaminoparthenolide | 0.329999 | 0.010025 |
| TTK | Chelerythrine | 0.328776 | 0.010322 |
| PFN2 | Dromostanolone Propionate | -0.32808 | 0.010494 |
| MSC | AT-13387 | -0.32788 | 0.010543 |
| YJEFN3 | Pipobroman | 0.326547 | 0.010883 |
| FCN3 | E-7820 | 0.325875 | 0.011057 |
| EPO | LMP-400 | 0.325004 | 0.011286 |
| ITGAM | Thiotepa | 0.324584 | 0.011398 |
| CLDN6 | Salinomycin | 0.324077 | 0.011535 |
| PFN2 | Fostamatinib | -0.32404 | 0.011546 |
| EPO | 7-Hydroxystaurosporine | 0.323581 | 0.01167 |
| GNGT1 | Acetalax | 0.323102 | 0.011801 |
| HOXA7 | Arsenic trioxide | -0.31965 | 0.012787 |
| PFN2 | Tamoxifen | -0.31484 | 0.014281 |
| EPO | Teniposide | 0.314589 | 0.014364 |
| ITGAM | Vinorelbine | -0.31416 | 0.014504 |
| KIF24 | Lificguat | -0.31384 | 0.014609 |
| FCN3 | BN-2629 | 0.313432 | 0.014746 |
| KIF24 | Aminoflavone | -0.3131 | 0.014856 |
| GNGT1 | Olaparib | 0.31216 | 0.015177 |
| EPO | Trametinib | -0.31152 | 0.015398 |
| TEX15 | kahalide f | -0.31106 | 0.015559 |
| FCN3 | Triciribine phosphate | -0.31006 | 0.015911 |
| FCN3 | Nelfinavir | 0.309848 | 0.015988 |
| YJEFN3 | Idarubicin | 0.309101 | 0.016257 |
| YJEFN3 | Uracil mustard | 0.308598 | 0.016441 |
| ITGAM | RH1 | 0.308113 | 0.01662 |
| EPO | Etoposide | 0.306671 | 0.017162 |
| TTK | Actinomycin D | -0.30579 | 0.017498 |
| MSC | Dexrazoxane | -0.30578 | 0.017506 |
| PFN2 | Dacarbazine | -0.305 | 0.017809 |
| PFN2 | Obatoclax | -0.30474 | 0.017911 |
| CDH10 | Triethylenemelamine | 0.30473 | 0.017915 |
| ITGAM | Clofarabine | 0.304674 | 0.017937 |
| EPO | Raloxifene | 0.304326 | 0.018075 |
| PFN2 | Alectinib | -0.30417 | 0.018135 |
| CDH10 | Elliptinium Acetate | 0.304059 | 0.018181 |
| GNGT1 | Rapamycin | 0.303181 | 0.018535 |
| MSC | LDK-378 | -0.30286 | 0.018666 |
| YJEFN3 | Teniposide | 0.302452 | 0.018834 |
| YJEFN3 | E-7820 | -0.3017 | 0.019146 |
| GNGT1 | Sonidegib | 0.3012 | 0.019356 |
| CDH10 | Olaparib | 0.301124 | 0.019388 |
| HOXA7 | Midostaurin | 0.300572 | 0.019623 |
| GNGT1 | Raloxifene | 0.299916 | 0.019904 |
| ITGAM | Raltitrexed | 0.299915 | 0.019905 |
| YJEFN3 | Mitoxantrone | 0.299447 | 0.020108 |
| EPO | XK-469 | 0.299204 | 0.020214 |
| TTK | Elesclomol | -0.29861 | 0.020476 |
| YJEFN3 | Mitomycin | 0.298368 | 0.020583 |
| GNGT1 | bisacodyl, active ingredient of viraplex | 0.296388 | 0.02148 |
| PFN2 | Denileukin Diftitox Ontak | -0.2963 | 0.021518 |
| EPO | Imiquimod | 0.296111 | 0.021607 |
| TEX15 | Testolactone | 0.295981 | 0.021668 |
| FCN3 | Oxaliplatin | 0.295691 | 0.021803 |
| YJEFN3 | LMP-400 | 0.29543 | 0.021925 |
| YJEFN3 | Cisplatin | 0.295312 | 0.021981 |
| CDH10 | Thiotepa | 0.294711 | 0.022264 |
| FCN3 | Ixabepilone | 0.293577 | 0.022809 |
| PFN2 | Isotretinoin | -0.29268 | 0.023245 |
| SEPT14 | Elesclomol | 0.291753 | 0.023708 |
| CDH10 | Idarubicin | 0.290957 | 0.02411 |
| FCN3 | Homoharringtonine | 0.289659 | 0.024778 |
| MSC | Tyrothricin | -0.28929 | 0.024971 |
| TEX15 | Fostamatinib | 0.289159 | 0.025039 |
| PFN2 | Oxaliplatin | -0.2891 | 0.025069 |
| KIF24 | Nelarabine | 0.288379 | 0.025451 |
| HOXA7 | Hydrastinine HCl | -0.28815 | 0.025573 |
| EPO | Selumetinib | -0.28661 | 0.026405 |
| EPO | Valrubicin | 0.286005 | 0.026741 |
| CLDN6 | O-6-Benzylguanine | -0.28527 | 0.02715 |
| ITGAM | Chlorambucil | 0.285241 | 0.027167 |
| EPO | Chlorambucil | 0.285032 | 0.027285 |
| YJEFN3 | Trametinib | -0.28458 | 0.027544 |
| FCN3 | BEN | 0.28378 | 0.027999 |
| PFN2 | Hydroxyurea | -0.28298 | 0.028462 |
| YJEFN3 | Cobimetinib (isomer 1) | -0.28189 | 0.029108 |
| TEX15 | Hydroxyurea | 0.281457 | 0.029366 |
| ITGAM | Tamoxifen | -0.28118 | 0.029533 |
| TEX15 | Sonidegib | 0.280592 | 0.029888 |
| EPO | Raltitrexed | 0.279842 | 0.030348 |
| PFN2 | Perifosine | -0.27905 | 0.030839 |
| TEX15 | XL-147 | 0.278433 | 0.031227 |
| ITGAM | Uracil mustard | 0.278131 | 0.031419 |
| TEX15 | Triapine | 0.277914 | 0.031557 |
| ITGAM | Actinomycin D | -0.27712 | 0.032068 |
| EPO | Cobimetinib (isomer 1) | -0.27698 | 0.032156 |
| MSC | RH1 | -0.27696 | 0.032169 |
| HOXA7 | 6-Mercaptopurine | -0.27636 | 0.032561 |
| PFN2 | Bleomycin | 0.276287 | 0.032608 |
| HOXA7 | Pipamperone | -0.2762 | 0.032662 |
| MSC | Pralatrexate | -0.27606 | 0.032755 |
| CDH10 | Bosutinib | -0.27525 | 0.033292 |
| TTK | Depsipeptide | -0.27457 | 0.033749 |
| EPO | Pipobroman | 0.274241 | 0.033971 |
| KIF24 | Chelerythrine | 0.274225 | 0.033982 |
| MSC | Vemurafenib | 0.274058 | 0.034095 |
| FCN3 | Fenretinide | 0.273851 | 0.034236 |
| CDH10 | Mitoxantrone | 0.273807 | 0.034266 |
| TTK | Fludarabine | 0.27376 | 0.034298 |
| TEX15 | Chlorambucil | 0.273234 | 0.034659 |
| CLDN6 | Acetalax | 0.273026 | 0.034803 |
| YJEFN3 | Chlorambucil | 0.272997 | 0.034823 |
| ITGAM | Bleomycin | 0.272836 | 0.034934 |
| YJEFN3 | Pimozide | -0.27275 | 0.034996 |
| ITGAM | LMP-400 | 0.2722 | 0.035378 |
| PFN2 | 6-Mercaptopurine | -0.27185 | 0.035621 |
| EPO | Mitoxantrone | 0.27165 | 0.035765 |
| TEX15 | Cladribine | 0.271647 | 0.035767 |
| TEX15 | Fenretinide | 0.271249 | 0.03605 |
| TEX15 | Gemcitabine | 0.27087 | 0.036321 |
| MSC | Nitrogen mustard | -0.27076 | 0.0364 |
| EPO | Elesclomol | 0.270532 | 0.036563 |
| CDH10 | Epirubicin | 0.270266 | 0.036755 |
| PFN2 | Arsenic trioxide | -0.26956 | 0.037272 |
| ITGAM | Idarubicin | 0.268994 | 0.037685 |
| YJEFN3 | Cytarabine | 0.268858 | 0.037786 |
| CDH10 | Teniposide | 0.268821 | 0.037813 |
| ITGAM | Carfilzomib | -0.26857 | 0.037997 |
| PFN2 | Hypothemycin | -0.26857 | 0.038003 |
| TTK | Doxorubicin | -0.26856 | 0.038006 |
| SEPT14 | Idelalisib | -0.26842 | 0.03811 |
| YJEFN3 | Etoposide | 0.267967 | 0.03845 |
| TTK | Homoharringtonine | -0.26785 | 0.03854 |
| SEPT14 | Celecoxib | 0.267297 | 0.038956 |
| TEX15 | tfdu | 0.266658 | 0.039444 |
| PFN2 | Lomustine | -0.26665 | 0.039447 |
| HOXA7 | Nandrolone phenpropionate | 0.266399 | 0.039643 |
| EPO | Epirubicin | 0.265679 | 0.0402 |
| FCN3 | Tegafur | 0.264969 | 0.040757 |
| CLDN6 | Ponatinib | -0.26486 | 0.040847 |
| EPO | Nitrogen mustard | 0.264782 | 0.040904 |
| SEPT14 | AT-13387 | -0.26476 | 0.040923 |
| YJEFN3 | Gemcitabine | 0.264599 | 0.04105 |
| EPO | Vincristine | 0.264522 | 0.04111 |
| GNGT1 | kahalide f | 0.263795 | 0.041691 |
| MSC | Streptozocin | 0.263634 | 0.04182 |
| TTK | PX-316 | 0.262558 | 0.042694 |
| EPO | BN-2629 | 0.262101 | 0.04307 |
| SEPT14 | Entinostat | 0.262084 | 0.043084 |
| YJEFN3 | Temsirolimus | 0.26201 | 0.043145 |
| ITGAM | LMP776 | 0.261923 | 0.043217 |
| TEX15 | Salinomycin | 0.260824 | 0.044134 |
| TEX15 | BEN | 0.260778 | 0.044172 |
| TEX15 | Nitrogen mustard | 0.260222 | 0.044642 |
| EPO | Thiotepa | 0.260215 | 0.044648 |
| EPO | RH1 | 0.260066 | 0.044775 |
| TTK | Elliptinium Acetate | -0.25976 | 0.045038 |
| PFN2 | Parthenolide | -0.25973 | 0.045066 |
| TTK | Epirubicin | -0.25958 | 0.045187 |
| YJEFN3 | Selumetinib | -0.25939 | 0.04535 |
| FCN3 | Lomustine | 0.258812 | 0.045853 |
| TEX15 | Lapachone | 0.25784 | 0.046704 |
| PFN2 | 8-Chloro-adenosine | -0.25741 | 0.047082 |
| CDH10 | Bleomycin | 0.257364 | 0.047125 |
| CDH10 | Gemcitabine | 0.257102 | 0.047358 |
| CDH10 | Topotecan | 0.256875 | 0.047561 |
| CDH10 | Carboplatin | 0.256686 | 0.04773 |
| EPO | Triethylenemelamine | 0.256649 | 0.047763 |
| FCN3 | XL-147 | 0.256128 | 0.048233 |
| CDH10 | Irinotecan | 0.25527 | 0.049014 |
| TTK | Isotretinoin | -0.25525 | 0.049035 |
| PFN2 | Ibrutinib | 0.254375 | 0.049841 |
