# Supplementary material for: Comprehensive multiomics analysis of cuproptosis-related gene characteristics in hepatocellular carcinoma
Source: Front Genet. 2022 Sep 6;13:942387. doi: 10.3389/fgene.2022.942387 (PMC9486098; doi:10.3389/fgene.2022.942387)
Supplement: Supplementary file 12 [file Table6.DOCX]

Table S6. Univariate and multivariate Cox analyses of the clinicopathological features and risk score in the TCGA-HCC cohort.

|  | Univariate Cox analysis | | | Multivariate Cox analysis | | |
| --- | --- | --- | --- | --- | --- | --- |
|  | HR | CI95 | P.Value | HR | CI95 | P.Value |
| Age | 1.25 | 0.88-1.77 | 0.214 | NA | NA | NA |
| Gender | 0.82 | 0.57-1.16 | 0.26 | NA | NA | NA |
| Hepatitis | 0.49 | 0.33-0.71 | 0 | 0.61 | 0.4-0.91 | 0.016* |
| Histologic Grade | 1.15 | 0.81-1.64 | 0.446 | NA | NA | NA |
| Stage | 2.45 | 1.72-3.47 | 0 | 0.87 | 0.12-6.35 | 0.891 |
| Surgical Margin | 2.24 | 1.37-3.66 | 0.001 | 1.21 | 0.72-2.01 | 0.469 |
| T stage | 2.52 | 1.77-3.58 | 0 | 1.91 | 0.26-14.04 | 0.527 |
| Vascular Invasion | 1.79 | 1.27-2.54 | 0.001 | 1.1 | 0.75-1.61 | 0.635 |
| Risk score | 2.72 | 2.25-3.28 | 0 | 2.45 | 2.03-2.97 | 0*** |

*P< 0.05, **P< 0.01, ***P<0.001.
